# Targeting wild-type Erythrocyte receptors for Plasmodium *falciparum* and *vivax* Merozoites *by* Zinc Finger Nucleases *In- silico*: Towards a Genetic Vaccine against Malaria

**DOI:** 10.1186/1479-0556-10-8

**Published:** 2012-08-31

**Authors:** Henry Kajumbula, Wilson Byarugaba, Misaki Wayengera

**Affiliations:** 1 Dept of Medical Microbiology, School of Biomedical Science, College of Health Sciences, Makerere University, P O Box 7072, Kampala, Uganda; 2 Dept of Biochemistry, Kampala International University Western Campus, P O Box 61, Ishaka, Bushenyi, Uganda; 3 Unit of Genetics & Genomics, Dept of Pathology, School of Biomedical Science, College of Health Sciences, Makerere University, P O Box 7072, Kampala, Uganda

**Keywords:** Malaria, *Plasmodium*, P. *falciparum*, P. *vivax*, Merozoites, RBC-receptors, Darc, Glycophorin A, Zinc finger nucleases, Host-pathway, Abrogation, Genetic vaccine

## Abstract

**Background:**

Malaria causes immense human morbidity and mortality globally. The plasmodium species *vivax* and *falciparum* cause over 75 % clinical malaria cases. Until now, gene-based strategies against malaria have only been applied to plasmodium species and their mosquito-vector. Merozoites of these two respective plasmodium *species* target and invade red blood cells (RBCs) by using the duffy antigen receptor for chemokines (DARC), and Sialic Acid (SLC4A1) residues of the O-linked glycans of Glycophorin A. RBCs of naturally selected duffy-negative blacks are resistant to P.*vivax* tropism. We hypothesized that artificial aberration of the host-pathway by target mutagenesis of either RBC –receptors, may abolish or reduce susceptibility of the host to malaria. As a first step towards the experimental actualization of these concepts, we aimed to identify zinc finger arrays (ZFAs) for constructing ZFNs that target genes of either wild-type host-RBC- receptors.

**Methods:**

*In-Silico* Gene & Genome Informatics

**Results:**

Using the genomic contextual nucleotide-sequences of homo-*sapiens* darc and glycophorin-*a*, and the ZFN-consortia software- CoDA-ZiFiT-ZFA and CoDA-ZiFiT-ZFN: we identified 163 and over 1,000 single zinc finger *arrays* (sZFAs) that bind sequences within the genes for the two respective RBC-receptors. S*econd,* 2 and 18 paired zinc finger arrays (pZFAs) that are precursors for zinc finger nucleases (ZFNs) capable of cleaving the genes for darc and glycophorin-*a* were respectively assembled. *Third,* a mega-BLAST evaluation of the genome-wide cleavage specificity of this set of ZFNs was done, revealing alternate homologous nucleotide targets in the human genome other than darc or glycophorin A.

**Conclusions:**

ZFNs engineered with these ZFA-precursors--with further optimization to enhance their specificity to only darc and glycophorin-*a*, could be used in constructing an experimental gene-based-malaria vaccine. Alternatively, meganucleases and transcription activator-like (TAL) nucleases that target conserved stretches of darc and glycophorin-*a* DNA may serve the purpose of abrogating invasion of RBCs by *falciparam* and *vivax* plasmodia species.

## Background

Malaria is an infectious cause of immense human-morbidity and mortality world-over [1]. About 250 million cases of malaria are reported annually. Despite presence of effective Artemisinin-based combination chemotherapy for treating clinical malaria, the disease still claims over 1 million lives annually, most-children under the ages of 5 years [2,3]. Attempts to eradicate malaria through controlling the binomics of its vector-the female *anopheles* mosquito through use of insecticides are contravened by fears of toxicity and potential risk of evolution of resistance to DDT, the would be ideal agent [4]. As a result, malaria continues to cause not just individual morbidity and mortality, but significant economic losses. Up-to 1.3 % decline in gross domestic product (GDP) is experienced within countries with high levels of transmission. Overall, within malaria endemic regions of the tropics and sub-tropics, clinical malaria is responsible for up to: 40 % of public health expenditures, 30 % to 50 % of inpatient hospital admissions, and 60 % of outpatient health clinic visits [1-4].

Malaria is caused by species of protozoa belonging to the genus *Plasmodium*[1,5]*.* There are well over 100 different species of *plasmodia* and the parasite is capable of infecting many animal species such as reptiles, birds, and various mammals. Nonetheless, only five Plasmodium *species:* P. *falciparum,* P. *vivax*, P. *malariae,* P. *ovale,* and P. *knowlesi* have been recognized to infect and cause clinical-malaria in humans [6]. Plasmodium *knowlesi,* a species that was previously only known to naturally infect macaques, has in recent years been recognized to cause zoonotic malaria in humans [5,7]. The Plasmodia species *falciparum* and *vivax* are the most common, causing over 70 % of all cases of clinical malaria globally [7].

Considering that *falciparum* is the most deadly plasmodia species, majority efforts to devise a malaria preventive vaccine have focused on it [7]. A safe and effective P.*falciparum* targeting subunit malaria vaccine however remains to be demonstrated [8,9]. Indeed, some have argued that the complexity of the malaria parasite precludes the successful development of a sub-unit vaccine, thereby resorting to use of whole-live-attenuated P. *falciparum* as vaccine-candidates [10,11].

The life-cycle of *plasmodia* has both a mosquito- and host-based sub-division. The hallmark of clinical malaria in man is, *however,* defined by invasion of red blood cells (RBCs or erythrocytes) by the parasites [12,13]. P. *vivax* and -*falciparum*, each utilize unique receptors present on the surface membrane of erythrocytes for their invasion. *On one hand*, the duffy antigen/receptor for chemokine (DARC) is the receptor for merozoites of Plasmodium *vivax* and Plasmodium *knowlesi;* and for chemokines [14-17]. A single T to C substitution at nucleotide −46 in the exon of the DARC gene (darc) is common among Duffy-negative blacks with a silent FY*B allele. The same leads to impairment of the promoter activity in erythroid cells by disrupting a binding site for the GATA1 erythroid transcription factor [18], thereby resulting into RBC-resistance to invasion by P.*vivax* merozoites. *On the other hand*, sialic acid (SLC4A1) residues of the O-linked glycans of the major intrinsic membrane protein of erythrocytes, Glycophorin A, are the major receptors for P.*falciparum* invasion of RBCs [19,20].

Given the prevailing challenges to the development of an effective malaria vaccine, we hypothesized that target mutagenesis of the well characterized host RBC-receptors for P. *falciparum* and P. *vivax*, may reduce global incidence of malaria. Mercereau-Puijalon & Ménard [21] have recently reported work to suggest that absolute dependence on the presence of Duffy on the red cell for P. *vivax* infection and development into the red cell is not true, since in some parts of the world, P. *vivax* infects and causes disease in Duffy-negative people. Elsewhere, targeted gene disruption studies of PfRh-1 and −2 genes of P. *falciparum* ligands for SLC4A1-residues by Triglia T et al.*,*[22] and Sahar T et al.*,*[23] have previously yielded mutants incapable of sialic acid-dependent invasion of human erythrocytes. As is the case for evidence to challenge DARC as the only erythrocyte-receptor for P.v*ivax* merozoites [21], therefore, those P.*falciparum* parasites that are mutated in PfRH- 1 and 2 proteins are known to invade Sialic acids defective-RBCs normally, by using ligand-receptor interactions pathways that are independent of SLC4A1-residues, and are neuraminidase-resistant [22,23]. Arguably, such plasmodia mutants capable of using alternate receptors to invade RBCs are likely to be still rare, and their selective adaptability poor.

Zinc finger *nucleases* - ZFNs - which are artificial, hybrid restriction enzymes [reviewed in ref. 24, 25], have recently become a powerful tool for primary edition of host genomes as a strategy to halt pathogen infectivity [24,25]. Perez E et al*.*[24], Holt N et al. [25], and Wilen CB et al. [26] have previously demonstrated the establishment of HIV-1 resistance in CD4+ T cells through generation of a double-strand break (DSB) at predetermined sites in the CCR5 coding region upstream of the natural CCR5D32 mutation using engineered ZFNs targeting human CCR5. As an initial step towards the experimentation of a similar approach against malaria, we aimed to identify zinc finger arrays (ZFAs) that are precursors of zinc finger nucleases (ZFNs) to be used for mutating wild-type host-RBC- receptors for merozoites of the most prevalent malaria parasites: P.-*vivax* and - *falciparum*.

## Methods

The overall design of this study was Bioinformatics (*In-Silico* gene/genome informatics). There was no requirement for approval from the authors institutional review boards (IRB).

### A. Identity of unpaired zinc finger arrays (sZFAs) binding darc/glycophorin-*a*

#### Materials and software

Genomic contextual- DNA-sequences of the homo-*sapiens* darc [GenBank IDs: 2532; genomic loci 1q21-q22] & glycophorin-*a* [GenBank IDs: Gene ID: 2993; and genomic 4q31.21] genes; along with the ZFN-consortia software- CoDA-ZiFiT-ZFA.

#### Interventions

The 2.49 kb-darc and 31.45 kb glycophorin-*a* genomic contextual*-* nucleotide sequences were separately fed into the CoDA-ZiFiT-ZFA user interface. Analysis was done according to the prescribed user protocols [25-27]. The CoDA-ZiFiT-ZFA software was used at the preset default settings, with specification for 3 ZF-array size (recognizing 9 nucleotides).

### B. Assembly of paired zinc finger arrays (pZFAs) binding darc/glycophorin-*a*

#### Materials and software

Genomic contextual- DNA-sequences of the homo-*sapiens* darc [GenBank IDs: 2532; genomic loci 1q21-q22] & glycophorin-*a* [GenBank IDs: Gene ID: 2993; and genomic 4q31.21] genes; along with the ZFN-consortia software- CoDA-ZiFiT-ZFN [25-27].

#### Interventions

The 2.49 kb-darc and 31.45 kb glycophorin-*a* long genomic contextual*-* nucleotide sequences were separately fed into the CoDA-ZiFiT-ZFN user interface. Analyses were conducted as per prescribed user protocols [25-27]. The CoDA-ZiFiT-ZFN software was used at the preset default settings, with specification for 5, 7, and 6 ‘spacer’ regions between the specificity sites of the pair of zinc finger array-precursors of ZFN.

### C. Mega-BLAST search for potential off-target sequences in the human genome

#### Materials and software

Target-cleavage sites for the model-ZFNs shown in Table 1, and the human genome build 37.3 along with its associated BLAST-N software.

**Table 1:** **Two paired ZFAs (pZFAs) required to engineer ZFNs that cleave within the human genomic context of the darc and glycophorin *a *genes**

**Zinc Finger Nuclease (ZFN)**	**Left Fn α-Helix; triplet**	**Right Fn α-Helix; triplet**
**-darc**		
ZFN-unknown-SP-5-1	F1; SPSKLVR; (GCG)	F1; SKKSLTR; (GCC)
1745 tGCCCTCTTCAGCAT**TGTGGTGCC**c 1769	F2; RQDNLGR; (GAG)	F2; EAHHLSR; (GGT)
1745 a**CGGGAGAAG**TCGTAACACCACGGg 1769	F3; QRNNLGR; (GAA)	F3; QPHGLAH; (TGT)
**-glycophorin-*a***		
ZFN-ASSEMBLY-SP-7-4	F1; RSSHLKM; (AGG)	F1; RRVDLL; (GCC)
8812	F2; QRSDLTR; (GCT)	F2; RQDNLGR; (GGT)
tCCTAGCTACTTGGGAG**GCTGAGGCA**g 8838		
8812 a**GGATCGATG**AACCCTCCGACTCCGTc 8838	F3; QSGTLTR; (GTA)	F3; VSNTLTR; (GCT)

#### Interventions

Each of the ZFN’s target nucleotide sequences were separately fed into the blast-n interface at default and off-target sequences identified as homologous sequences (>100 % identity).

### D. Software and Database Availability

The ZFN consortium CoDA-ZiFiT-ZFA/ZFN software and algorithms used are available at the following url: http://www.zincfingers.org/scientific-background.htm

The NCBI human genome build 37.3 database hosting the complete darc and glycophorin-*a* genomic contextual DNA sequences used, is available at the following url: http://www.ncbi.nlm.nih.gov/genome/guide/human/

## Results

### Identity of unpaired zinc finger arrays to bind darc and glycophorin-a sequences

**Figure 1:**
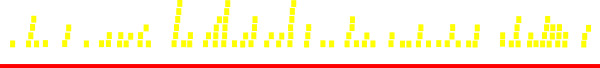
**Schematics of target DNA-binding by ZFAs along the darc gene.** This figure graphically illustrates the sites and frequency of single ZFAs that bind to nucleotide sequences within the context human darc gene. Note that there are ZFAs capable of sequentially binding at almost all positions within the darc gene.

We identified 165 and close to 1,000 unpaired or single zinc finger arrays (sZFAs) that bind the sequential nucleotide sequences constituting the genes for the two erythrocyte receptors for the merozoites of P. *vivax* and - *falciparum* (see Additional file 1 for darc binding sZFAs, Additional file 2 for glycophorin-*a* binding sZFAs, respectively). In principle, these zinc fingers are protein-motifs that have two beta strands and an alpha helix. The three sets of strands are stabilized through coordination of a zinc ion mediated by pairs of conserved cysteine and histidine residues [27]. Within the context of this complex, residues −1 to 6 of the alpha-helix of the ZFAs are responsible for the recognition of triplets of DNA sequences through the formation of base-specific contacts in the major groove of the double-stranded target DNA. These so-called ‘recognition residues’ are listed in N- to C-terminal direction [28,29]. As a consequence, the recognition sequences of the ZFA bind target DNA sites in a reverse pattern (amino acids 1–6 of the ‘recognition’ alpha helix bind onto consecutive nucleotides in DNA in the *3'* to *5'* direction) [30]. An illustration of the distribution of the target-DNA-binding sites for the unpaired or single (sZFA) identified in our study, along the gene-context of the homo-*sapien* darc gene is shown in Figure 1. Details of the pattern of the ZFA-binding to DNA of sialic acids within glycophorin-*a* are not shown.

### Assembly of paired zinc finger array (pZFA)-precursors of nucleases to cleave darc and glycophoin-a sequences

**Figure 2:**
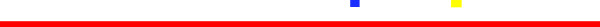
**Schematics of cleavage-sites by ZFNs along the darc gene.** This figure graphically illustrates the sites and frequency of ZFN cleavage of the darc gene. Two ZFN cleaving at positions approximately 0.5 and 0.75 within the context the darc gene are shown, although others could be re-engineered by the methods described in the text [22,23,28]. Hits (blue, green, and gold bars) represent targets along the gene (red bars). ZFN hits in the graphic are color-coded based on spacer size (5 bp = Blue; 6 bp = Green; 7 bp = Gold).

**Figure 3:**
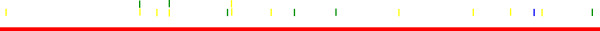
**Schematics cleavage-sites by ZFNs along the glycophorin-*a* gene.** This figure graphically illustrates the sites and frequency of ZFN cleavage within the human glycophorin-***a*** gene. Note the presence of ZFNs cleaving at positions approximately 0.02 and 0.92 within the context of the glycophorin-***a*** gene. These are suited for the complete excision of this gene from the genomes of transduced erythroid precursor cells (through non-homologous end-joining), although many more ZFNs with specific-disruptive potential to only specified areas of the same gene, may be constructed. Hits (blue, green, and gold bars) represent targets along the gene (red bars). ZFN hits in the graphic are color-coded based on spacer size (5 bp = Blue; 6 bp = Green; 7 bp = Gold).

We assembled another set of *2* and *18* paired zinc finger arrays (pZFA) that are candidate precursors for engineering nucleases (ZFNs) that cleave within the context of nucleotide sequences of the genes encoding the same erythrocyte receptors- DARC and Glycophorin A respectively (see file Additional 3 for darc specific ZFNs, and Additional file 4 for some of the glycophorin A specific ZFNs). The consensus amino-acids sequences of the DNA binding domains of the the paired ZFAs (left and right-helix) which constitute nucleases cleaving within darc and glycophorin-a nucleotide sequences are shown in table I. The actual ZFNs can artificially be constructed by recombinantly linking the coding-nucleotide sequences of any pair of ZFAs to the DNA-cleavage domain of the Flavobacterium *okeanokoites* type II class endonuclease, F_N_ (Fok I) as previously simulated *in-vivo* by Kim, et al. [30]. The resulting ZFNs bind as dimers to their target DNA sites *in-vivo*, with dimerization of the ZFNs-monomers being mediated by the FokI cleavage domain through cleavage of a five or six base pair ‘spacer’ sequence that separates the two inverted target ‘half sites’ [27-29,31,32]. It should be noted, that the DNA-binding specificities of zinc finger domains can be re-engineered using one of various methods, enabling customized ZFNs to be constructed that target nearly any gene sequence [27]. The distribution of the target cleavage sites of the model-ZFNs along the length of the genomic contextual sequences of the darc and gyclophorin-*a* genes are respectively shown in Figures 2 and 3.

### c). Mega-Blast search for potential off-target homologs in the human genome

In order to further examine the specificity of the model-ZFNs in (b) above for darc and glycophorin-a, we conducted a mega-BLAST survey for genes within the NCBI human build 37.3 genome whose corresponding nucleotide-sequences are homologous to the target sequences of the two model-ZFNs targeting darc and glycophorin-*a* shown in Table 1 (detailed results available as query IDs: IcI |56721, and IcI |45777). Several genes with the predicament of yielding off-target cleavages were identified. It is important to note that, such potential off-target genes are likely to be more, given that Pattanayak V et al. [33] and Gabriel R et al. [34] have recently demonstrated that off-target cleavage specificities are best revealed by in-vitro rather than *in-silico* selection methods. These data uniquely underline the need for further optimization of the set of ZFNs identified in our study prior to in-vitro and in-vivo experimental trail. Such optimization should include but not necessary be limited to: (i) in-vitro optimization using a bacteria-one hybrid (B1H) or yeast-one-hybrid (Y1H) system [35] and (ii) modifications to the cleavage domain in order to generate a hybrid capable of functionally interrogating the ZFN dimer interface so as to prevent homodimerization, while still enhancing the efficiency of cleavage [36].

## Discussion

We explore ZFA-precursors of ZFNs that may be used for target mutagenesis and abrogation of RBC-receptors for merozoites of two major malaria causing plasmodium. The complexity of the malaria causing parasite-plasmodium, which precludes the development of an effective sub-unit malaria vaccine, has led to the evolution of gene-based strategies that aim to modify and attenuate either plasmodium [10,11] or the mosquito-vector [4]. Basing on the knowledge base that--merozoites of the two most prevalent Plasmodium *speciesfalciparum* and *vivax* respectively, by using- the Duffy Antigen Receptor for Chemokines (DARC), and Sialic Acid (SLC4A1) residues of the O-linked glycans of Glycophorin A specifically target and invade red blood cells (RBCs), we hypothesized that artificial aberration of either host-pathway by target mutagenesis of the respective RBC –receptors, may abolish or reduce susceptibility of the host to malaria [14-23]. As shown in Figure 1 and supporting files 1 and 2, single or unpaired ZFAs (sZFAs) that bind to sequential nucleotide sequences within the genomic context of the darc and gycophorin-*a* genes were identified. Another set of paired-ZFAs (pZFAs) to use for engineering ZFNs that target and cleave within genes encoding either plasmodium RBC-receptors was assembled (see Figures 2 and 3, and supporting files 3 and 4). On mega-blast evaluation, however, several homologous off target sequences (which may be accessed online as NCBI-human genome-BLAST query/result IDs: IcI |56721, and IcI |45) were uncovered within the human genome. Arguably, multiple ways to practically reduce ZFN cleavage at off-target sites are available. For instance, (i) chosing a target that has minimal homology with other sites in the genome, (iii) avoiding the use of high affinity zinc-finger DNA –binding domains, (iii) and using the lowest concentration of ZFN to perform cleavage of the target gene, are some. As already noted above in section c of our results, improvements in design are also possible by (i) in-vitro optimization using a bacteria-one hybrid (B1H) or yeast-one-hybrid (Y1H) system [35] and (ii) modifications to the cleavage domain in order to generate a hybrid capable of functionally interrogating the ZFN dimer interface so as to prevent homodimerization, while still enhancing the efficiency of cleavage [36]. Alternatively, however, transcription activator-like effector nucleases (TALENs) and or mega-endonucleases are alternative designer nucleases that recognize longer DNA sequence and potentially reduce the off-target cleavage [37]. The prevailing limitations in the in-vitro and or in-situ capacity of our laboratory, *non-the-less,* stood in the way of experimental trial of such necessary optimizations and improvements.

We propose that, using the paired zinc finger arrays (pZFA), ZFNs that cleave within the genomic context of the darc and glycophorin *a* gene sequences may be engineered using the protocol previously described by Kim, et al. [30]. Once this is achieved, the issuing-ZFNs could be experimentally usable to abolish the respective host-pathways for RBC infectivity by the most two prevalent malaria parasites. Such a strategy—when translated to the clinic may drastically reduce the global malaria incidence. The feasibility of this approach is supported by existing evidence pointing to resistance of RBCs of naturally selected duffy-negative blacks to P.*vivax* tropism [14-18,21]. However, as is further discussed below regarding mutants capable of invading RBCs using alternative pathways, keen readers will observe that the ground for making such an argument is not solid. In principle, recombinant strategies for uniquely expressing a diploid copy (really pair) of the above ZFN-genotypes within- erythroid precursor cells or reticulocytes rather than other host cells, should offer us a host-targeted gene-therapy and preventive vaccine against malaria. For example, recombinant vectors based on the human Parvovirus B19 that naturally target erythroid progenitor cells, or lentiviral vectors whose tropism has been adapted in-vivo to erythroid progenitor cell-lines may suffice [38-42]. Progenitor cell-lines of RBCs form the right target for this type of genetic vaccine, because of two major reasons. *First,* immature RBCs have usually not yet lost their nuclei, while adult RBCs lose their nuclei in the process of specialization to enhance the volume occupied by the oxygen carrying molecule, hemoglobin. *Second,* adult RBCs are sequestered every three to four months, being replaced by new ones arising from the stem cells in bone marrow. The complexity and expensive-nature of these propositions relative to the available knowledge-base and capacity on ground within high-malaria endemic areas of the world are challenges to note. It is our hope that these challenges will not abstruse the scientific quest for the necessary pre-clinical data to support the safety and efficacy of the same within cell-line or animal models. Perhaps such data may help gunner support for the eventual clinical trial of this approach. At this point, it is important to re-emphasize the prior findings by Mercereau-Puijalon and Ménard [21] challenging absolute dependence on the presence of Duffy on the red cell for P. *vivax* infection and development into the red cell is not true. Similarly, P.*falciparum* parasites that are mutated in PfRH- 1 and 2 proteins have equally been described [19,20]. The global prevalence and distribution of such mutant *plasmodia* is however not known [21]. In addition, the P.*falciparum* cited to be mutated in PfRH- 1 and 2 proteins were only laboratory clones rather than naturally selected mutants— a fact that makes the natural evolution of such mutants to remain uncertain [19,20]. Theoretically, one could also argue that mutants in these major receptors may be selectively disadvantaged over the wild-type *plasmodia.* Therefore, while there is scanty data pointing to the existence of *plasmodia* capable of invading RBCs independent of these two major receptors, their influence on the efficacy of our proposed gene-therapeutic approach remains uncertain. Specifically, our proposed gene-vaccination strategy against malaria can only be said to be unsustainable in the face of evolutionary-dynamics of *plasmodia* versus host wherein plasmodia keep adapting to use alternate RBC-receptors. Nevertheless, the favorability of this pattern of natural selection and fitness of the issuing mutants over wild-type *plasmodia,* remains to be examined. Overall, given the conflicting reports, it is ideal that actual experiments—which are beyond the existing capacity in our laboratory, are conducted to test whether ZFNs against DARC and glycophoin-*a* indeed prevent P. *vivax* and P. *falciparum* infection.

## Conclusion

We argue that ZFNs engineered with these ZFA-precursors—given the appropriate optimization in-vitro to enhance their specificity to only darc and sialic acids, could potentially be applied to the development of an experimental gene-based-Malaria vaccine. Alternatively, meganucleases and transcription activator-like (TAL) nucleases that recognize longer stretches of darc and glycophorin-*a* DNA may serve the specific purpose of abrogating invasion of RBCs by *falciparam* and *vivax* plasmodia species.

In-vivo disruption of these genes within only RBCs should be effectible using *either* erythroid progenitor or reticulocyte specific recombinant parvovirus *or* lentiviral vectors that deliver and transduce a diploid copy of the optimized DARC and Sialic acid specific ZFNs.

## Competing interests

WM, KH, and WB are affiliated to Restrizymes Biotherapeutics (U) Ltd.

## Authors’ contributions

WM conceived the idea for this article, designed and undertook the experiments. KH & WB provided oversight on the experimental activities by WM. WM, KH & BW wrote and revised the initial draft ms. All authors read and approved the final manuscript.

## Supplementary Material

Additional file 1 ** A list of the ZFAs binding to sequences of the human darc-gene.** This file offers a detailed list of the *163* ZFAs that bind sequences of the human darc-gene. Click here for file

Additional file 2 ** A list of 168 ZFAs binding to sequences within the human glycophorin-*a *gene.** This file details the recognition domains of the 168 ZFAs that bind to nucleotide sites within the first 6, 873 bp of the human glycophorin-*a* gene. Overall, there were ZFAs capable of binding to sequences across the entire 31,451 bp of the human glycophorin-*a* gene. Click here for file

Additional file 3 ** A list of the 1–6 recognition domains of the alpha-helix of one of the paired ZFAs for engineering ZFNs that cleave the human darc-gene.** This file offers a detailed list of the 1–6 recognition domains of the alpha-helix of one of the paired ZFAs for engineering ZFNs that cleave the human darc-gene. Click here for file

Additional file 4 ** A list of the 1–6 recognition domains of the alpha-helix of one of the paired ZFAs for engineering ZFNs that cleave the human glycophorin-*a *gene.** This file offers details of the 1–6 recognition domians (denoted F1, F2, F3/F3, F2, F1) of the alpha-helix for the 18 assembled paired ZFAs for engineering ZFNs that cleave within the human glycophorin-*a* gene. Click here for file
