# Supplementary material for: Targeting wild-type Erythrocyte receptors for Plasmodium falciparum and vivax Merozoites by Zinc Finger Nucleases In- silico: Towards a Genetic Vaccine against Malaria
Source: Genet Vaccines Ther. 2012 Aug 31;10:8. doi: 10.1186/1479-0556-10-8 (PMC3500210; doi:10.1186/1479-0556-10-8)
Supplement: Additional file 1 — A list of the ZFAs binding to sequences of the human darc-gene. This file offers a detailed list of the 163 ZFAs that bind sequences of the human darc-gene. [file 1479-0556-10-8-S1.pdf]

- **Zinc Finger Site Type:** Array
  Zinc Finger Engineering Method: CoDA
  Sequence Name: Gene ID: 2532, HUMAN DARC **Showing 2.49kb region from base 159173803 to 159176290.**

  Sequence Length:2,490
  Nucleotide Sequence
  :nCCCCCAGTAGAAATATGAATAGAAATCACCCTGTGGGCAATGGT
  CCCATTTTAAAATATGCTGTCCCATTGTCCCCTAGAGCCTACTTTAA
  CTTGTCAGACCATGTATTCCACTTCATATGCAAGAGGCATGCACTG
  AGCCCATAGGTGGCTAGGCAAACACCCAATAGCTCCCTGAAATGG
  CTTCATTATGCAGCCTCGACAGCCACCCCAACCCTCCCACTCTCAC
  ACTGAAACACCCAGACCTAGAGATAGCTAGACACACCCAGACACC
  CGCCAAGCCCCTCACATACAGATATGTGCACAATGATACACAGCAA
  ACGTACACAGAGTTCAGTACACACAAAGAGCTCACGCCCACGTGC
  ACACACCCCTCAGTTGGGACAGAGTTGACCACCACCACCTTTCTC
  CCAAACACATGGCTTTTGAACTGCCTTTCCTTGGATCCAGTTCAAG
  GGGATGGAGGAGCAGTGAGAGTCAGCCGCCCTTCCACTCCAATTT
  CCCAGCACCTCCCTTATCTCTGCCTCACAAGTCACCCAGCCCCCCT
  CTCTTCCTTCCTTGTGCTTGAAGAATCTCTCCTTGCTGGAAAGCCC
  CCTGTTTTCTCAATCTCCCTTTCCACTTCGGTAAAATCTCTACTTGC
  TGGAAAGCCCCCTGTTTTCTCAATCTCCCTTTCCACTTCGGTAAAAT
  GCCCACTTTCTGGTCCCCACCTTTTTCCTGAGTGTAGTCCCAACCA
  GCCAAATCCAACCTCAAAACAGGAAGACCCAAGGCCAGTGACCCC
  CATAGGCCTGAGGCTTGTGCAGGCAGTGGGCGTGGGGTAAGGCT
  TCCTGATGCCCCCTGTCCCTGCCCAGAACCTGATGGCCCTCATTA
  GTCCTTGGCTCTTATCTTGGAAGCACAGGCGCTGACAGCCGTCCC
  AGCCCTTCTGTCTGCGGGCCTGAACCAAACGGTGCCATGGGGAAC
  TGTCTGCACAGGGTGAGTATGGGGCCAGGCCCCAGAGTCCCTTAT
  CCCTATGCCCCTCATTTCCCGTGCTGTTTGCCCCTCAGTCTTTATAT
  CTCTTCCTTTTCCTCCTCATCTTTTCTCCCTTCCTGCTTTTTTCCTCT
  TCCTTCAAAGTCTTTTTCCTTCTCTCCTTCCTATGCTAGCCTCCTAG
  CTCCCTCTTGTGTCCCTCCCTTTGCCTTTGAGTCAGTTCCATCCTG
  GTCTCTTGGTGCCTTTTCCTTCTGACCTTGCACTGCTCCTCCAGCC
  CCAGCTGCCCTGGCTTCCCCAGGACTGTTCCTGCTCCGGCTCTTC
  AGGCTCCCTGCTTTGTCCTTTTCCACTGTCCGCACTGCATCTGACT
  CCTGCAGAGACCTTGTTCTCCCACCCGACCTTCCTCTCTGTCCTCC
  CCTCCCACCTGCCCCTCAATTCCCAGGAGACTCTTCCGGTGTAACT
  CTGATGGCCTCCTCTGGGTATGTCCTCCAGGCGGAGCTCTCCCCC
  TCAACTGAGAACTCAAGTCAGCTGGACTTCGAAGATGTATGGAATT

CTTCCTATGGTGTGAATGATTCCTTCCCAGATGGAGACTATGGTGC
CAACCTGGAAGCAGCTGCCCCCTGCCACTCCTGTAACCTGCTGGA
TGACTCTGCACTGCCCTTCTTCATCCTCACCAGTGTCCTGGGTATC
CTAGCTAGCAGCACTGTCCTCTTCATGCTTTTCAGACCTCTCTTCC
GCTGGCAGCTCTGCCCTGGCTGGCCTGTCCTGGCACAGCTGGCT
GTGGGCAGTGCCCTCTTCAGCATTGTGGTGCCCGTCTTGGCCCCA
GGGCTAGGTAGCACTCGCAGCTCTGCCCTGTGTAGCCTGGGCTAC
TGTGTCTGGTATGGCTCAGCCTTTGCCCAGGCTTTGCTGCTAGGG
TGCCATGCCTCCCTGGGCCACAGACTGGGTGCAGGCCAGGTCCC
AGGCCTCACCCTGGGGCTCACTGTGGGAATTTGGGGAGTGGCTG
CCCTACTGACACTGCCTGTCACCCTGGCCAGTGGTGCTTCTGGTG
GACTCTGCACCCTGATATACAGCACGGAGCTGAAGGCTTTGCAGG
CCACACACACTGTAGCCTGTCTTGCCATCTTTGTCTTGTTGCCATT
GGGTTTGTTTGGAGCCAAGGGGCTGAAGAAGGCATTGGGTATGGG
GCCAGGCCCCTGGATGAATATCCTGTGGGCCTGGTTTATTTTCTGG
TGGCCTCATGGGGTGGTTCTAGGACTGGATTTCCTGGTGAGGTCC
AAGCTGTTGCTGTTGTCAACATGTCTGGCCCAGCAGGCTCTGGAC
CTGCTGCTGAACCTGGCAGAAGCCCTGGCAATTTTGCACTGTGTG
GCTACGCCCCTGCTCCTCGCCCTATTCTGCCACCAGGCCACCCGC
ACCCTCTTGCCCTCTCTGCCCCTCCCTGAAGGATGGTCTTCTCATC
TGGACACCCTTGGAAGCAAATCCTAGTTCTCTTCCCACCTGTCAAC
CTGAATTAAAGTCTACACTGCCTTTGTGAAn

Selected Module Sets:
Selected Module Count: 3
Ignore Asp Overlap: True

The results below are zinc finger arrays that can be constructed using CoDA. Note that other methods (including modular assembly and OPEN) can also potentially be used to target the input sequence of interest.”

Sort By: Position

⊞ ZFA-unknown-1
117 tGAAGTGGAAt 107
117 aCTTCACCTTa 107

FINGER	HELIX	TRIPLET	REFERENCE NUMBER	SOURCE
F1	HKPNLHR	GAA	-	CoDA
F2	RREVLEN	GTG	-	CoDA

F3	QTVNLDR	GAA	-	CoDA

ZF DNA Sequence

Bos taurus (cow) Build 3.1

Blast GAAGTGGAA

ZFA-unknown-2
124 aGAGGCATGCa 134
124 tCTCCGTACGt 134

FINGER	HELIX	TRIPLET	REFERENCE NUMBER	SOURCE
F1	RNRNLVL	TGC	-	CoDA
F2	QSTTLKR	GCA	-	CoDA
F3	RGDNLNR	GAG	-	CoDA

ZF DNA Sequence

Bos taurus (cow) Build 3.1

Blast GAGGCATGC

ZFA-unknown-3
146 gGTGGCTAGGc 156
146 cCACCGATCCg 156

FINGER	HELIX	TRIPLET	REFERENCE NUMBER	SOURCE
F1	RSSHLKM	AGG	-	CoDA
F2	QRSDLTR	GCT	-	CoDA
F3	RPDALPR	GTG	-	CoDA

ZF DNA Sequence

Bos taurus (cow) Build 3.1

Blast GTGGCTAGG

ZFA-unknown-4
151 cTAGGCAAACa 161
151 gATCCGTTTGt 161

FINGER	HELIX	TRIPLET	REFERENCE NUMBER	SOURCE
F1	GHTALRN	AAC	-	CoDA

F2	QSTTLKR	GCA	-	CoDA
F3	RRDGLAG	TAG	-	CoDA

ZF DNA Sequence

Bos taurus (cow) Build 3.1

Blast TAGGCAAAC

ZFA-unknown-5
164 gGGTGTTTGCc 154
164 cCCACAAACGg 154

FINGER	HELIX	TRIPLET	REFERENCE NUMBER	SOURCE
F1	RMRNLII	TGC	-	CoDA
F2	HKSSLTR	GTT	-	CoDA
F3	HGHRLKT	GGT	-	CoDA

ZF DNA Sequence

Bos taurus (cow) Build 3.1

Blast GGTGTTTGC

ZFA-unknown-6
200 cGAGGCTGCAt 190
200 gCTCCGACGTa 190

FINGER	HELIX	TRIPLET	REFERENCE NUMBER	SOURCE
F1	HNGTLKR	GCA	-	CoDA
F2	QRSDLTR	GCT	-	CoDA
F3	RQDNLQR	GAG	-	CoDA

ZF DNA Sequence

Bos taurus (cow) Build 3.1

Blast GAGGCTGCA

ZFA-unknown-7
203 tGTCGAGGCTg 193
203 aCAGCTCCGAc 193

FINGER	HELIX	TRIPLET	REFERENCE NUMBER	SOURCE

F1	NKQALDR	GCT	-	CoDA
F2	RQDNLGR	GAG	-	CoDA
F3	DRTPLNR	GTC	-	CoDA

ZF DNA Sequence

Bos taurus (cow) Build 3.1

Blast GTCGAGGCT

ZFA-unknown-8
206 gGCTGTCGAGg 196
206 cCGACAGCTCc 196

FINGER	HELIX	TRIPLET	REFERENCE NUMBER	SOURCE
F1	RTHNLKR	GAG	-	CoDA
F2	DHSSLKR	GTC	-	CoDA
F3	VSNSLAR	GCT	-	CoDA

ZF DNA Sequence

Bos taurus (cow) Build 3.1

Blast GCTGTCGAG

ZFA-unknown-9
209 gGTGGCTGTCg 199
209 cCACCGACAGc 199

FINGER	HELIX	TRIPLET	REFERENCE NUMBER	SOURCE
F1	TRAVLRR	GTC	-	CoDA
F2	QRSDLTR	GCT	-	CoDA
F3	RPDALPR	GTG	-	CoDA

ZF DNA Sequence

Bos taurus (cow) Build 3.1

Blast GTGGCTGTC

ZFA-unknown-10
212 tGGGGTGGCTg 202
212 aCCCCACCGAc 202

FINGER	HELIX	TRIPLET	REFERENCE NUMBER	SOURCE
F1	TKPILVR	GCT	-	CoDA
F2	RREVLEN	GTG	-	CoDA
F3	RQGHLKR	GGG	-	CoDA

ZF DNA Sequence

Bos taurus (cow) Build 3.1

Blast GGGGTGGCT

ZFA-unknown-11
213 tTGGGGTGGCt 203
213 aACCCCACCGa 203

FINGER	HELIX	TRIPLET	REFERENCE NUMBER	SOURCE
F1	VPSKLKR	GGC	-	CoDA
F2	EAHHLSR	GGT	-	CoDA
F3	RSDHLSL	TGG	-	CoDA

ZF DNA Sequence

Bos taurus (cow) Build 3.1

Blast TGGGGTGGC

ZFA-unknown-12
215 gGTTGGGGTGg 205
215 cCAACCCCACc 205

FINGER	HELIX	TRIPLET	REFERENCE NUMBER	SOURCE
F1	SRFTLGR	GTG	-	CoDA
F2	RREHLVR	GGG	-	CoDA
F3	INHSLRR	GTT	-	CoDA

ZF DNA Sequence

Bos taurus (cow) Build 3.1

Blast GTTGGGGTG

ZFA-unknown-13
222 gTGGGAGGGTt 212

222 cACCCTCCCAa 212

FINGER	HELIX	TRIPLET	REFERENCE NUMBER	SOURCE
F1	TRQKLET	GGT	-	CoDA
F2	RQDNLGR	GAG	-	CoDA
F3	RMDHLAG	TGG	-	CoDA

ZF DNA Sequence

Bos taurus (cow) Build 3.1

Blast TGGGAGGGT

ZFA-unknown-14
223 aGTGGGAGGGt 213
223 tCACCCTCCCa 213

FINGER	HELIX	TRIPLET	REFERENCE NUMBER	SOURCE
F1	KKDHLHR	GGG	-	CoDA
F2	QSAHLKR	GGA	-	CoDA
F3	RNTALQH	GTG	-	CoDA

ZF DNA Sequence

Bos taurus (cow) Build 3.1

Blast GTGGGAGGG

ZFA-unknown-15
245 gGTCTGGGTGt 235
245 cCAGACCCACa 235

FINGER	HELIX	TRIPLET	REFERENCE NUMBER	SOURCE
F1	RNFILQR	GTG	-	CoDA
F2	RREHLTI	TGG	-	CoDA
F3	DRSSLRR	GTC	-	CoDA

ZF DNA Sequence

Bos taurus (cow) Build 3.1

Blast GTCTGGGTG

ZFA-unknown-16
264 gTGTGTCTAGc 254
264 cACACAGATCg 254

FINGER	HELIX	TRIPLET	REFERENCE NUMBER	SOURCE
F1	RGTNLRT	TAG	-	CoDA
F2	DHSSLKR	GTC	-	CoDA
F3	QPHGLAH	TGT	-	CoDA

ZF DNA Sequence

Bos taurus (cow) Build 3.1

Blast TGTGTCTAG

ZFA-unknown-17
268 cTGGGTGTGTc 258
268 gACCCACACAg 258

FINGER	HELIX	TRIPLET	REFERENCE NUMBER	SOURCE
F1	RRQALEY	TGT	-	CoDA
F2	RREVLEN	GTG	-	CoDA
F3	RRDHLSL	TGG	-	CoDA

ZF DNA Sequence

Bos taurus (cow) Build 3.1

Blast TGGGTGTGT

ZFA-unknown-18
271 tGTCTGGGTGt 261
271 aCAGACCCACa 261

FINGER	HELIX	TRIPLET	REFERENCE NUMBER	SOURCE
F1	RNFILQR	GTG	-	CoDA
F2	RREHLTI	TGG	-	CoDA
F3	DRSSLRR	GTC	-	CoDA

ZF DNA Sequence

Bos taurus (cow) Build 3.1

Blast GTCTGGGTG

ZFA-unknown-19
277 gGCGGGTGTCt 267
277 cCGCCCACAGa 267

FINGER	HELIX	TRIPLET	REFERENCE NUMBER	SOURCE
F1	TRAVLRR	GTC	-	CoDA
F2	EAHHLSR	GGT	-	CoDA
F3	RLDMLAR	GCG	-	CoDA

ZF DNA Sequence

Bos taurus (cow) Build 3.1

Blast GCGGGTGTC

ZFA-unknown-20
278 tGGCGGGTGTc 268
278 aCCGCCCACAg 268

FINGER	HELIX	TRIPLET	REFERENCE NUMBER	SOURCE
F1	RKQHLTL	TGT	-	CoDA
F2	RREHLVR	GGG	-	CoDA
F3	ESGHLKR	GGC	-	CoDA

ZF DNA Sequence

Bos taurus (cow) Build 3.1

Blast GGCGGGTGT

ZFA-unknown-21
290 aTGTGAGGGGc 280
290 tACACTCCCCg 280

FINGER	HELIX	TRIPLET	REFERENCE NUMBER	SOURCE
F1	RNTHLAR	GGG	-	CoDA
F2	RQDNLGR	GAG	-	CoDA
F3	QQHGLRH	TGT	-	CoDA

ZF DNA Sequence

Bos taurus (cow) Build 3.1

Blast TGTGAGGGG

ZFA-unknown-22
361 cGTGGGCGTGa 351
361 gCACCCGCACt 351

FINGER	HELIX	TRIPLET	REFERENCE NUMBER	SOURCE
F1	SRFTLGR	GTG	-	CoDA
F2	LKEHLTR	GGC	-	CoDA
F3	RKDALHV	GTG	-	CoDA

ZF DNA Sequence

Bos taurus (cow) Build 3.1

Blast GTGGGCGTG

ZFA-unknown-23
374 aGGGGTGTGTg 364
374 tCCCCACACAc 364

FINGER	HELIX	TRIPLET	REFERENCE NUMBER	SOURCE
F1	RRQALEY	TGT	-	CoDA
F2	RREVLEN	GTG	-	CoDA
F3	RQGHLKR	GGG	-	CoDA

ZF DNA Sequence

Bos taurus (cow) Build 3.1

Blast GGGGTGTGT

ZFA-unknown-24
376 tGAGGGGTGTg 366
376 aCTCCCCACAc 366

FINGER	HELIX	TRIPLET	REFERENCE NUMBER	SOURCE
F1	RKQHLTL	TGT	-	CoDA
F2	RREHLVR	GGG	-	CoDA
F3	RRDNLLR	GAG	-	CoDA

ZF DNA Sequence

Bos taurus (cow) Build 3.1

Blast GAGGGGTGT

ZFA-unknown-25
400 gGTGGTGGTCa 390
400 cCACCACCAGt 390

FINGER	HELIX	TRIPLET	REFERENCE NUMBER	SOURCE
F1	TMAVLRR	GTC	-	CoDA
F2	RREVLEN	GTG	-	CoDA
F3	RKDALHV	GTG	-	CoDA

ZF DNA Sequence

Bos taurus (cow) Build 3.1

Blast GTGGTGGTC

ZFA-unknown-26
401 tGGTGGTGGTc 391
401 aCCACCACCAg 391

FINGER	HELIX	TRIPLET	REFERENCE NUMBER	SOURCE
F1	MKHHLAR	GGT	-	CoDA
F2	EAHHLSR	GGT	-	CoDA
F3	IRHHLKR	GGT	-	CoDA

ZF DNA Sequence

Bos taurus (cow) Build 3.1

Blast GGTGGTGGT

ZFA-unknown-27
403 gGTGGTGGTGg 393
403 cCACCACCACc 393

FINGER	HELIX	TRIPLET	REFERENCE NUMBER	SOURCE
F1	RNFVLAR	GTG	-	CoDA
F2	RREVLEN	GTG	-	CoDA
F3	RKDALHV	GTG	-	CoDA

ZF DNA Sequence

Bos taurus (cow) Build 3.1

Blast GTGGTGGTG

ZFA-unknown-28
404 aGGTGGTGGTg 394
404 tCCACCACCAc 394

FINGER	HELIX	TRIPLET	REFERENCE NUMBER	SOURCE
F1	MKHHLAR	GGT	-	CoDA
F2	EAHHLSR	GGT	-	CoDA
F3	IRHHLKR	GGT	-	CoDA

ZF DNA Sequence

Bos taurus (cow) Build 3.1

Blast GGTGGTGGT

ZFA-unknown-29
411 gGGAGAAAGGt 401
411 cCCTCTTTCCa 401

FINGER	HELIX	TRIPLET	REFERENCE NUMBER	SOURCE
F1	RMAHLHA	AGG	-	CoDA
F2	QQTNLTR	GAA	-	CoDA
F3	QTTHLSR	GGA	-	CoDA

ZF DNA Sequence

Bos taurus (cow) Build 3.1

Blast GGAGAAAGG

ZFA-unknown-30
454 aGGGGATGGAg 464
454 tCCCCTACCTc 464

FINGER	HELIX	TRIPLET	REFERENCE NUMBER	SOURCE
F1	DKTKLRV	GGA	-	CoDA
F2	VRHNLTR	GAT	-	CoDA
F3	RGDKLGP	GGG	-	CoDA

ZF DNA Sequence

Bos taurus (cow) Build 3.1

Blast GGGGATGGA

ZFA-unknown-31
457 gGATGGAGGAg 467
457 cCTACCTCCTc 467

FINGER	HELIX	TRIPLET	REFERENCE NUMBER	SOURCE
F1	RTDRLIR	GGA	-	CoDA
F2	QSAHLKR	GGA	-	CoDA
F3	ISHNLAR	GAT	-	CoDA

ZF DNA Sequence

Bos taurus (cow) Build 3.1

Blast GATGGAGGA

ZFA-unknown-32
460 tGGAGGAGCAg 470
460 aCCTCCTCGTc 470

FINGER	HELIX	TRIPLET	REFERENCE NUMBER	SOURCE
F1	KNTRLSV	GCA	-	CoDA
F2	QSAHLKR	GGA	-	CoDA
F3	QMSHLKR	GGA	-	CoDA

ZF DNA Sequence

Bos taurus (cow) Build 3.1

Blast GGAGGAGCA

ZFA-unknown-33
463 aGGAGCAGTGa 473
463 tCCTCGTCACt 473

FINGER	HELIX	TRIPLET	REFERENCE NUMBER	SOURCE
F1	RSHILTN	GTG	-	CoDA
F2	QSTTLKR	GCA	-	CoDA

F3	QKPHLSR	GGA	-	CoDA

ZF DNA Sequence

Bos taurus (cow) Build 3.1

Blast GGAGCAGTG

⊞ ZFA-unknown-34
485 gGCGGCTGACt 475
485 cCGCCGACTGa 475

FINGER	HELIX	TRIPLET	REFERENCE NUMBER	SOURCE
F1	DLSNLKR	GAC	-	CoDA
F2	QRSDLTR	GCT	-	CoDA
F3	RLDMLAR	GCG	-	CoDA

ZF DNA Sequence

Bos taurus (cow) Build 3.1

Blast GCGGCTGAC

⊞ ZFA-unknown-35
493 aGTGGAAGGGc 483
493 tCACCTTCCCg 483

FINGER	HELIX	TRIPLET	REFERENCE NUMBER	SOURCE
F1	KRERLDR	GGG	-	CoDA
F2	QQTNLTR	GAA	-	CoDA
F3	RNVALGN	GTG	-	CoDA

ZF DNA Sequence

Bos taurus (cow) Build 3.1

Blast GTGGAAGGG

⊞ ZFA-unknown-36
496 tGGAGTGGAAg 486
496 aCCTCACCTTc 486

FINGER	HELIX	TRIPLET	REFERENCE NUMBER	SOURCE
F1	HKPNLHR	GAA	-	CoDA

F2	RREVLEN	GTG	-	CoDA
F3	QKPHLSR	GGA	-	CoDA

ZF DNA Sequence

Bos taurus (cow) Build 3.1

Blast GGAGTGGAA

ZFA-unknown-37
510 aGGTGCTGGGa 500
510 tCCACGACCCt 500

FINGER	HELIX	TRIPLET	REFERENCE NUMBER	SOURCE
F1	KKDHLHR	GGG	-	CoDA
F2	QRSDLTR	GCT	-	CoDA
F3	HGHRLKT	GGT	-	CoDA

ZF DNA Sequence

Bos taurus (cow) Build 3.1

Blast GGTGCTGGG

ZFA-unknown-38
513 gGGAGGTGCTg 503
513 cCCTCCACGAc 503

FINGER	HELIX	TRIPLET	REFERENCE NUMBER	SOURCE
F1	LRQTLAR	GCT	-	CoDA
F2	EAHHLSR	GGT	-	CoDA
F3	QNSHLRR	GGA	-	CoDA

ZF DNA Sequence

Bos taurus (cow) Build 3.1

Blast GGAGGTGCT

ZFA-unknown-39
527 tGAGGCAGAGa 517
527 aCTCCGTCTCt 517

FINGER	HELIX	TRIPLET	REFERENCE NUMBER	SOURCE

F1	KHSNLAR	GAG	-	CoDA
F2	QSTTLKR	GCA	-	CoDA
F3	RGDNLNR	GAG	-	CoDA

ZF DNA Sequence

Bos taurus (cow) Build 3.1

Blast GAGGCAGAG

ZFA-unknown-40
530 tTGTGAGGCAg 520
530 aACACTCCGTc 520

FINGER	HELIX	TRIPLET	REFERENCE NUMBER	SOURCE
F1	RRVDLLR	GCA	-	CoDA
F2	RQDNLGR	GAG	-	CoDA
F3	QQHGLRH	TGT	-	CoDA

ZF DNA Sequence

Bos taurus (cow) Build 3.1

Blast TGTGAGGCA

ZFA-unknown-41
542 gGGCTGGGTGa 532
542 cCCGACCCACt 532

FINGER	HELIX	TRIPLET	REFERENCE NUMBER	SOURCE
F1	RNFILQR	GTG	-	CoDA
F2	RREHLTI	TGG	-	CoDA
F3	EKSHLTR	GGC	-	CoDA

ZF DNA Sequence

Bos taurus (cow) Build 3.1

Blast GGCTGGGTG

ZFA-unknown-42
544 gGGGGCTGGGt 534
544 cCCCCGACCCa 534

FINGER	HELIX	TRIPLET	REFERENCE NUMBER	SOURCE
F1	KKDHLHR	GGG	-	CoDA
F2	QRSDLTR	GCT	-	CoDA
F3	RTEHLAR	GGG	-	CoDA

ZF DNA Sequence

Bos taurus (cow) Build 3.1

Blast GGGGCTGGG

ZFA-unknown-43
548 aGAGGGGGGCt 538
548 tCTCCCCCCGa 538

FINGER	HELIX	TRIPLET	REFERENCE NUMBER	SOURCE
F1	TNSKLTR	GGC	-	CoDA
F2	RREHLVR	GGG	-	CoDA
F3	RRDNLLR	GAG	-	CoDA

ZF DNA Sequence

Bos taurus (cow) Build 3.1

Blast GAGGGGGGC

ZFA-unknown-44
619 aGTGGAAAGGg 609
619 tCACCTTTCCc 609

FINGER	HELIX	TRIPLET	REFERENCE NUMBER	SOURCE
F1	RMAHLHA	AGG	-	CoDA
F2	QQTNLTR	GAA	-	CoDA
F3	RNVALGN	GTG	-	CoDA

ZF DNA Sequence

Bos taurus (cow) Build 3.1

Blast GTGGAAAGG

ZFA-unknown-45
622 cGAAGTGGAAa 612

622 gCTTCACCTTt 612

FINGER	HELIX	TRIPLET	REFERENCE NUMBER	SOURCE
F1	HKPNLHR	GAA	-	CoDA
F2	RREVLEN	GTG	-	CoDA
F3	QTVNLDR	GAA	-	CoDA

ZF DNA Sequence

Bos taurus (cow) Build 3.1

Blast GAAGTGGAA

ZFA-unknown-46
676 aGTGGAAAGGg 666
676 tCACCTTTCCc 666

FINGER	HELIX	TRIPLET	REFERENCE NUMBER	SOURCE
F1	RMAHLHA	AGG	-	CoDA
F2	QQTNLTR	GAA	-	CoDA
F3	RNVALGN	GTG	-	CoDA

ZF DNA Sequence

Bos taurus (cow) Build 3.1

Blast GTGGAAAGG

ZFA-unknown-47
679 cGAAGTGGAAa 669
679 gCTTCACCTTt 669

FINGER	HELIX	TRIPLET	REFERENCE NUMBER	SOURCE
F1	HKPNLHR	GAA	-	CoDA
F2	RREVLEN	GTG	-	CoDA
F3	QTVNLDR	GAA	-	CoDA

ZF DNA Sequence

Bos taurus (cow) Build 3.1

Blast GAAGTGGAA

ZFA-unknown-48
708 aGGTGGGGACc 698
708 tCCACCCCTGg 698

FINGER	HELIX	TRIPLET	REFERENCE NUMBER	SOURCE
F1	EEANLRR	GAC	-	CoDA
F2	RREHLVR	GGG	-	CoDA
F3	VDHHLRR	GGT	-	CoDA

ZF DNA Sequence

Bos taurus (cow) Build 3.1

Blast GGTGGGGAC

ZFA-unknown-49
748 tGAGGTTGGAt 738
748 aCTCCAACCTa 738

FINGER	HELIX	TRIPLET	REFERENCE NUMBER	SOURCE
F1	RSTHLRV	GGA	-	CoDA
F2	HKSSLTR	GTT	-	CoDA
F3	RHDQLTR	GAG	-	CoDA

ZF DNA Sequence

Bos taurus (cow) Build 3.1

Blast GAGGTTGGA

ZFA-unknown-50
786 tGAGGCTTGTg 796
786 aCTCCGAACAc 796

FINGER	HELIX	TRIPLET	REFERENCE NUMBER	SOURCE
F1	KRQHLEY	TGT	-	CoDA
F2	QRSDLTR	GCT	-	CoDA
F3	RQDNLQR	GAG	-	CoDA

ZF DNA Sequence

Bos taurus (cow) Build 3.1

Blast GAGGCTTGT

ZFA-unknown-51
792 tTGTGCAGGCa 802
792 aACACGTCCGt 802

FINGER	HELIX	TRIPLET	REFERENCE NUMBER	SOURCE
F1	VPSKLLR	GGC	-	CoDA
F2	QSTTLKR	GCA	-	CoDA
F3	QAHGLTA	TGT	-	CoDA

ZF DNA Sequence

Bos taurus (cow) Build 3.1

Blast TGTGCAGGC

ZFA-unknown-52
799 gGCAGTGGGCg 809
799 cCGTCACCCGc 809

FINGER	HELIX	TRIPLET	REFERENCE NUMBER	SOURCE
F1	SPSKLVR	GGC	-	CoDA
F2	RREVLEN	GTG	-	CoDA
F3	QGGTLRR	GCA	-	CoDA

ZF DNA Sequence

Bos taurus (cow) Build 3.1

Blast GCAGTGGGC

ZFA-unknown-53
802 aGTGGGCGTGg 812
802 tCACCCGCACc 812

FINGER	HELIX	TRIPLET	REFERENCE NUMBER	SOURCE
F1	SRFTLGR	GTG	-	CoDA
F2	LKEHLTR	GGC	-	CoDA
F3	RKDALHV	GTG	-	CoDA

ZF DNA Sequence

Bos taurus (cow) Build 3.1

Blast GTGGGCGTG

ZFA-unknown-54
810 tGGGGTAAGGc 820
810 aCCCCATTCCg 820

FINGER	HELIX	TRIPLET	REFERENCE NUMBER	SOURCE
F1	RRAHLRQ	AGG	-	CoDA
F2	QRSSLVR	GTA	-	CoDA
F3	RTEHLAR	GGG	-	CoDA

ZF DNA Sequence

Bos taurus (cow) Build 3.1

Blast GGGGTAAGG

ZFA-unknown-55
847 cTGGGCAGGGa 837
847 gACCCGTCCCt 837

FINGER	HELIX	TRIPLET	REFERENCE NUMBER	SOURCE
F1	RRAHLQN	GGG	-	CoDA
F2	QSTTLKR	GCA	-	CoDA
F3	RSDHLSL	TGG	-	CoDA

ZF DNA Sequence

Bos taurus (cow) Build 3.1

Blast TGGGCAGGG

ZFA-unknown-56
893 aGGCGCTGACa 903
893 tCCGCGACTGt 903

FINGER	HELIX	TRIPLET	REFERENCE NUMBER	SOURCE
F1	DLSNLKR	GAC	-	CoDA
F2	QRSDLTR	GCT	-	CoDA
F3	ESGHLRR	GGC	-	CoDA

ZF DNA Sequence

Bos taurus (cow) Build 3.1

Blast GGCGCTGAC

ZFA-unknown-57
913 cTGGGACGGCt 903
913 gACCCTGCCGa 903

FINGER	HELIX	TRIPLET	REFERENCE NUMBER	SOURCE
F1	APSKLDR	GGC	-	CoDA
F2	DRGNLTR	GAC	-	CoDA
F3	RSDHLSL	TGG	-	CoDA

ZF DNA Sequence

Bos taurus (cow) Build 3.1

Blast TGGGACGGC

ZFA-unknown-58
915 gGCTGGGACGg 905
915 cCGACCCTGCc 905

FINGER	HELIX	TRIPLET	REFERENCE NUMBER	SOURCE
F1	KNNDLTR	ACG	-	CoDA
F2	RREHLVR	GGG	-	CoDA
F3	VSNSLAR	GCT	-	CoDA

ZF DNA Sequence

Bos taurus (cow) Build 3.1

Blast GCTGGGACG

ZFA-unknown-59
967 cTGTGCAGACa 957
967 gACACGTCTGt 957

FINGER	HELIX	TRIPLET	REFERENCE NUMBER	SOURCE
F1	EEVNLRR	GAC	-	CoDA
F2	QSTTLKR	GCA	-	CoDA
F3	QAHGLTA	TGT	-	CoDA

ZF DNA Sequence

Bos taurus (cow) Build 3.1

Blast TGTGCAGAC

ZFA-unknown-60
1013 aGGGGCATAGg 1003
1013 tCCCCGTATCc 1003

FINGER	HELIX	TRIPLET	REFERENCE NUMBER	SOURCE
F1	RSHNLKL	TAG	-	CoDA
F2	QSTTLKR	GCA	-	CoDA
F3	RTEHLAR	GGG	-	CoDA

ZF DNA Sequence

Bos taurus (cow) Build 3.1

Blast GGGGCATAG

ZFA-unknown-61
1023 tGCTGTTTGCc 1033
1023 aCGACAAACGg 1033

FINGER	HELIX	TRIPLET	REFERENCE NUMBER	SOURCE
F1	RMRNLII	TGC	-	CoDA
F2	HKSSLTR	GTT	-	CoDA
F3	VSNTLTR	GCT	-	CoDA

ZF DNA Sequence

Bos taurus (cow) Build 3.1

Blast GCTGTTTGC

ZFA-unknown-62
1036 aGGGGCAAACa 1026
1036 tCCCCGTTTGt 1026

FINGER	HELIX	TRIPLET	REFERENCE NUMBER	SOURCE
F1	GHTALRN	AAC	-	CoDA
F2	QSTTLKR	GCA	-	CoDA
F3	RTEHLAR	GGG	-	CoDA

ZF DNA Sequence

Bos taurus (cow) Build 3.1

Blast GGGGCAAAC

ZFA-unknown-63
1067 tGAGGAGGAAa 1057
1067 aCTCCTCCTTt 1057

FINGER	HELIX	TRIPLET	REFERENCE NUMBER	SOURCE
F1	QASNLLR	GAA	-	CoDA
F2	RQDNLGR	GAG	-	CoDA
F3	RVDNLPR	GAG	-	CoDA

ZF DNA Sequence

Bos taurus (cow) Build 3.1

Blast GAGGAGGAA

ZFA-unknown-64
1070 aGATGAGGAGg 1060
1070 tCTACTCCTCc 1060

FINGER	HELIX	TRIPLET	REFERENCE NUMBER	SOURCE
F1	RQMNLDR	GAG	-	CoDA
F2	RQDNLGR	GAG	-	CoDA
F3	LNSNLAR	GAT	-	CoDA

ZF DNA Sequence

Bos taurus (cow) Build 3.1

Blast GATGAGGAG

ZFA-unknown-65
1086 aGCAGGAAGGg 1076
1086 tCGTCCTTCCc 1076

FINGER	HELIX	TRIPLET	REFERENCE NUMBER	SOURCE
F1	RPHHLDA	AGG	-	CoDA
F2	QSAHLKR	GGA	-	CoDA

F3	QDVSLVR	GCA	-	CoDA

ZF DNA Sequence

Bos taurus (cow) Build 3.1

Blast GCAGGAAGG

ZFA-unknown-66
1099 gGAAGAGGAAa 1089
1099 cCTTCTCCTTt 1089

FINGER	HELIX	TRIPLET	REFERENCE NUMBER	SOURCE
F1	QASNLLR	GAA	-	CoDA
F2	RQDNLGR	GAG	-	CoDA
F3	QRNNLGR	GAA	-	CoDA

ZF DNA Sequence

Bos taurus (cow) Build 3.1

Blast GAAGAGGAA

ZFA-unknown-67
1127 gGAAGGAGAGa 1117
1127 cCTTCCTCTCt 1117

FINGER	HELIX	TRIPLET	REFERENCE NUMBER	SOURCE
F1	KHSNLTR	GAG	-	CoDA
F2	QSAHLKR	GGA	-	CoDA
F3	LGENLRR	GAA	-	CoDA

ZF DNA Sequence

Bos taurus (cow) Build 3.1

Blast GAAGGAGAG

ZFA-unknown-68
1130 aTAGGAAGGAg 1120
1130 tATCCTTCCTc 1120

FINGER	HELIX	TRIPLET	REFERENCE NUMBER	SOURCE
F1	RMERLDR	GGA	-	CoDA

F2	QQTNLTR	GAA	-	CoDA
F3	RRDHLSL	TAG	-	CoDA

ZF DNA Sequence

Bos taurus (cow) Build 3.1

Blast TAGGAAGGA

ZFA-unknown-69
1143 cTAGGAGGCTa 1133
1143 gATCCTCCGAt 1133

FINGER	HELIX	TRIPLET	REFERENCE NUMBER	SOURCE
F1	NKQALDR	GCT	-	CoDA
F2	RQDNLGR	GAG	-	CoDA
F3	RPESLRP	TAG	-	CoDA

ZF DNA Sequence

Bos taurus (cow) Build 3.1

Blast TAGGAGGCT

ZFA-unknown-70
1151 aGAGGGAGCTa 1141
1151 tCTCCCTCGAt 1141

FINGER	HELIX	TRIPLET	REFERENCE NUMBER	SOURCE
F1	TTQALRR	GCT	-	CoDA
F2	QSAHLKR	GGA	-	CoDA
F3	VHWNLMR	GAG	-	CoDA

ZF DNA Sequence

Bos taurus (cow) Build 3.1

Blast GAGGGAGCT

ZFA-unknown-71
1229 aGGAGCAGTGc 1219
1229 tCCTCGTCACg 1219

FINGER	HELIX	TRIPLET	REFERENCE NUMBER	SOURCE

F1	RSHILTN	GTG	-	CoDA
F2	QSTTLKR	GCA	-	CoDA
F3	QKPHLSR	GGA	-	CoDA

ZF DNA Sequence

Bos taurus (cow) Build 3.1

Blast GGAGCAGTG

⊞ ZFA-unknown-72
1232 tGGAGGAGCAg 1222
1232 aCCTCCTCGTc 1222

FINGER	HELIX	TRIPLET	REFERENCE NUMBER	SOURCE
F1	KNTRLSV	GCA	-	CoDA
F2	QSAHLKR	GGA	-	CoDA
F3	QMSHLKR	GGA	-	CoDA

ZF DNA Sequence

Bos taurus (cow) Build 3.1

Blast GGAGGAGCA

⊞ ZFA-unknown-73
1235 gGCTGGAGGAg 1225
1235 cCGACCTCCTc 1225

FINGER	HELIX	TRIPLET	REFERENCE NUMBER	SOURCE
F1	RTDRLIR	GGA	-	CoDA
F2	QSAHLKR	GGA	-	CoDA
F3	LKHDLRR	GCT	-	CoDA

ZF DNA Sequence

Bos taurus (cow) Build 3.1

Blast GCTGGAGGA

⊞ ZFA-unknown-74
1238 tGGGGCTGGAg 1228
1238 aCCCCGACCTc 1228

FINGER	HELIX	TRIPLET	REFERENCE NUMBER	SOURCE
F1	RPAKLVL	GGA	-	CoDA
F2	QRSDLTR	GCT	-	CoDA
F3	RTEHLAR	GGG	-	CoDA

ZF DNA Sequence

Bos taurus (cow) Build 3.1

Blast GGGGCTGGA

ZFA-unknown-75
1241 aGCTGGGGCTg 1231
1241 tCGACCCCGAc 1231

FINGER	HELIX	TRIPLET	REFERENCE NUMBER	SOURCE
F1	THSMLAR	GCT	-	CoDA
F2	RREHLVR	GGG	-	CoDA
F3	VSNSLAR	GCT	-	CoDA

ZF DNA Sequence

Bos taurus (cow) Build 3.1

Blast GCTGGGGCT

ZFA-unknown-76
1244 gGCAGCTGGGg 1234
1244 cCGTCGACCCc 1234

FINGER	HELIX	TRIPLET	REFERENCE NUMBER	SOURCE
F1	KKDHLHR	GGG	-	CoDA
F2	QRSDLTR	GCT	-	CoDA
F3	QGGTLRR	GCA	-	CoDA

ZF DNA Sequence

Bos taurus (cow) Build 3.1

Blast GCAGCTGGG

ZFA-unknown-77
1256 tGGGGAAGCCa 1246

1256 aCCCCTTCGGt 1246

FINGER	HELIX	TRIPLET	REFERENCE NUMBER	SOURCE
F1	DSPTLRR	GCC	-	CoDA
F2	QQTNLTR	GAA	-	CoDA
F3	RIDKLGG	GGG	-	CoDA

ZF DNA Sequence

Bos taurus (cow) Build 3.1

Blast GGGGAAGCC

ZFA-unknown-78
1273 cGGAGCAGGAa 1263
1273 gCCTCGTCCTt 1263

FINGER	HELIX	TRIPLET	REFERENCE NUMBER	SOURCE
F1	RTDRLIR	GGA	-	CoDA
F2	QSTTLKR	GCA	-	CoDA
F3	QKPHLSR	GGA	-	CoDA

ZF DNA Sequence

Bos taurus (cow) Build 3.1

Blast GGAGCAGGA

ZFA-unknown-79
1276 aGCCGGAGCAg 1266
1276 tCGGCCTCGTc 1266

FINGER	HELIX	TRIPLET	REFERENCE NUMBER	SOURCE
F1	KNTRLSV	GCA	-	CoDA
F2	QSAHLKR	GGA	-	CoDA
F3	DPSNLRR	GCC	-	CoDA

ZF DNA Sequence

Bos taurus (cow) Build 3.1

Blast GCCGGAGCA

ZFA-unknown-80
1322 aGATGCAGTGc 1312
1322 tCTACGTCACg 1312

FINGER	HELIX	TRIPLET	REFERENCE NUMBER	SOURCE
F1	RSHILTN	GTG	-	CoDA
F2	QSTTLKR	GCA	-	CoDA
F3	LNSNLAR	GAT	-	CoDA

ZF DNA Sequence

Bos taurus (cow) Build 3.1

Blast GATGCAGTG

ZFA-unknown-81
1332 tGCAGGAGTCa 1322
1332 aCGTCCTCAGt 1322

FINGER	HELIX	TRIPLET	REFERENCE NUMBER	SOURCE
F1	TSTLLKR	GTC	-	CoDA
F2	QSAHLKR	GGA	-	CoDA
F3	QDVSLVR	GCA	-	CoDA

ZF DNA Sequence

Bos taurus (cow) Build 3.1

Blast GCAGGAGTC

ZFA-unknown-82
1350 gTGGGAGAACa 1340
1350 cACCCTCTTGt 1340

FINGER	HELIX	TRIPLET	REFERENCE NUMBER	SOURCE
F1	GHTALRN	AAC	-	CoDA
F2	RQDNLGR	GAG	-	CoDA
F3	RMDHLAG	TGG	-	CoDA

ZF DNA Sequence

Bos taurus (cow) Build 3.1

Blast TGGGAGAAC

ZFA-unknown-83
1351 gGTGGGAGAAc 1341
1351 cCACCCTCTTg 1341

FINGER	HELIX	TRIPLET	REFERENCE NUMBER	SOURCE
F1	QRSNLAR	GAA	-	CoDA
F2	QSAHLKR	GGA	-	CoDA
F3	RNTALQH	GTG	-	CoDA

ZF DNA Sequence

Bos taurus (cow) Build 3.1

Blast GTGGGAGAA

ZFA-unknown-84
1355 gTCGGGTGGGa 1345
1355 cAGCCCACCCt 1345

FINGER	HELIX	TRIPLET	REFERENCE NUMBER	SOURCE
F1	KGDHLRR	GGG	-	CoDA
F2	EAHHLSR	GGT	-	CoDA
F3	RADGLQL	TCG	-	CoDA

ZF DNA Sequence

Bos taurus (cow) Build 3.1

Blast TCGGGTGGG

ZFA-unknown-85
1363 aGAGGAAGGTc 1353
1363 tCTCCTTCCAg 1353

FINGER	HELIX	TRIPLET	REFERENCE NUMBER	SOURCE
F1	TKQRLEV	GGT	-	CoDA
F2	QQTNLTR	GAA	-	CoDA
F3	RRDNLNR	GAG	-	CoDA

ZF DNA Sequence

Bos taurus (cow) Build 3.1

Blast GAGGAAGGT

ZFA-unknown-86
1375 aGGGGAGGACa 1365
1375 tCCCCTCCTGt 1365

FINGER	HELIX	TRIPLET	REFERENCE NUMBER	SOURCE
F1	DEANLRR	GAC	-	CoDA
F2	RQDNLGR	GAG	-	CoDA
F3	RIDKLGG	GGG	-	CoDA

ZF DNA Sequence

Bos taurus (cow) Build 3.1

Blast GGGGAGGAC

ZFA-unknown-87
1378 gGGAGGGGAGg 1368
1378 cCCTCCCCTCc 1368

FINGER	HELIX	TRIPLET	REFERENCE NUMBER	SOURCE
F1	RNTNLTR	GAG	-	CoDA
F2	RREHLVR	GGG	-	CoDA
F3	QTTHLRR	GGA	-	CoDA

ZF DNA Sequence

Bos taurus (cow) Build 3.1

Blast GGAGGGGAG

ZFA-unknown-88
1380 gTGGGAGGGGa 1370
1380 cACCCTCCCCt 1370

FINGER	HELIX	TRIPLET	REFERENCE NUMBER	SOURCE
F1	RNTHLAR	GGG	-	CoDA
F2	RQDNLGR	GAG	-	CoDA
F3	RMDHLAG	TGG	-	CoDA

ZF DNA Sequence

Bos taurus (cow) Build 3.1

Blast TGGGAGGGG

ZFA-unknown-89
1381 gGTGGGAGGGg 1371
1381 cCACCCTCCCc 1371

FINGER	HELIX	TRIPLET	REFERENCE NUMBER	SOURCE
F1	KKDHLHR	GGG	-	CoDA
F2	QSAHLKR	GGA	-	CoDA
F3	RNTALQH	GTG	-	CoDA

ZF DNA Sequence

Bos taurus (cow) Build 3.1

Blast GTGGGAGGG

ZFA-unknown-90
1385 gGCAGGTGGGa 1375
1385 cCGTCCACCCt 1375

FINGER	HELIX	TRIPLET	REFERENCE NUMBER	SOURCE
F1	KGDHLRR	GGG	-	CoDA
F2	EAHHLSR	GGT	-	CoDA
F3	QNGTLTR	GCA	-	CoDA

ZF DNA Sequence

Bos taurus (cow) Build 3.1

Blast GCAGGTGGG

ZFA-unknown-91
1388 aGGGGCAGGTg 1378
1388 tCCCCGTCCAc 1378

FINGER	HELIX	TRIPLET	REFERENCE NUMBER	SOURCE
F1	TTTKLAI	GGT	-	CoDA
F2	QSTTLKR	GCA	-	CoDA
F3	RTEHLAR	GGG	-	CoDA

ZF DNA Sequence

Bos taurus (cow) Build 3.1

Blast GGGGCAGGT

ZFA-unknown-92
1409 gGAAGAGTCTc 1399
1409 cCTTCTCAGAg 1399

FINGER	HELIX	TRIPLET	REFERENCE NUMBER	SOURCE
F1	SKPNLKM	TCT	-	CoDA
F2	RQDNLGR	GAG	-	CoDA
F3	QRNNLGR	GAA	-	CoDA

ZF DNA Sequence

Bos taurus (cow) Build 3.1

Blast GAAGAGTCT

ZFA-unknown-93
1433 aGAGGAGGCCa 1423
1433 tCTCCTCCGGt 1423

FINGER	HELIX	TRIPLET	REFERENCE NUMBER	SOURCE
F1	VRKDLTR	GCC	-	CoDA
F2	RQDNLGR	GAG	-	CoDA
F3	RVDNLPR	GAG	-	CoDA

ZF DNA Sequence

Bos taurus (cow) Build 3.1

Blast GAGGAGGCC

ZFA-unknown-94
1432 cTGGGTATGTc 1442
1432 gACCCATACAg 1442

FINGER	HELIX	TRIPLET	REFERENCE NUMBER	SOURCE
F1	RKQHLQL	TGT	-	CoDA
F2	QRSSLVR	GTA	-	CoDA
F3	RSDHLSL	TGG	-	CoDA

ZF DNA Sequence

Bos taurus (cow) Build 3.1

Blast TGGGTATGT

ZFA-unknown-95
1447 aGGCGGAGCTc 1457
1447 tCCGCCTCGAg 1457

FINGER	HELIX	TRIPLET	REFERENCE NUMBER	SOURCE
F1	TTQALRR	GCT	-	CoDA
F2	QSAHLKR	GGA	-	CoDA
F3	EKSHLKR	GGC	-	CoDA

ZF DNA Sequence

Bos taurus (cow) Build 3.1

Blast GGCGGAGCT

ZFA-unknown-96
1464 aGGGGGAGAGc 1454
1464 tCCCCCTCTCg 1454

FINGER	HELIX	TRIPLET	REFERENCE NUMBER	SOURCE
F1	KHSNLTR	GAG	-	CoDA
F2	QSAHLKR	GGA	-	CoDA
F3	RTEHLAR	GGG	-	CoDA

ZF DNA Sequence

Bos taurus (cow) Build 3.1

Blast GGGGGAGAG

ZFA-unknown-97
1466 tGAGGGGGAGa 1456
1466 aCTCCCCCTCt 1456

FINGER	HELIX	TRIPLET	REFERENCE NUMBER	SOURCE
F1	RNTNLTR	GAG	-	CoDA
F2	RREHLVR	GGG	-	CoDA

F3	RRDNLLR	GAG	-	CoDA

ZF DNA Sequence

Bos taurus (cow) Build 3.1

Blast GAGGGGGAG

ZFA-unknown-98
1469 aGTTGAGGGGg 1459
1469 tCAACTCCCCc 1459

FINGER	HELIX	TRIPLET	REFERENCE NUMBER	SOURCE
F1	RNTHLAR	GGG	-	CoDA
F2	RQDNLGR	GAG	-	CoDA
F3	HHNSLTR	GTT	-	CoDA

ZF DNA Sequence

Bos taurus (cow) Build 3.1

Blast GTTGAGGGG

ZFA-unknown-99
1493 cGAAGATGTAt 1503
1493 gCTTCTACATa 1503

FINGER	HELIX	TRIPLET	REFERENCE NUMBER	SOURCE
F1	QQQALVR	GTA	-	CoDA
F2	VRHNLTR	GAT	-	CoDA
F3	QRNNLGR	GAA	-	CoDA

ZF DNA Sequence

Bos taurus (cow) Build 3.1

Blast GAAGATGTA

ZFA-unknown-100
1517 aTAGGAAGAAt 1507
1517 tATCCTTCTTa 1507

FINGER	HELIX	TRIPLET	REFERENCE NUMBER	SOURCE
F1	QASNLTR	GAA	-	CoDA

F2	QQTNLTR	GAA	-	CoDA
F3	RRDHLSL	TAG	-	CoDA

ZF DNA Sequence

Bos taurus (cow) Build 3.1

Blast TAGGAAGAA

ZFA-unknown-101
1539 cTGGGAAGGAa 1529
1539 gACCCTTCCTt 1529

FINGER	HELIX	TRIPLET	REFERENCE NUMBER	SOURCE
F1	RMERLDR	GGA	-	CoDA
F2	QQTNLTR	GAA	-	CoDA
F3	RSDHLSL	TGG	-	CoDA

ZF DNA Sequence

Bos taurus (cow) Build 3.1

Blast TGGGAAGGA

ZFA-unknown-102
1538 aGATGGAGACt 1548
1538 tCTACCTCTGa 1548

FINGER	HELIX	TRIPLET	REFERENCE NUMBER	SOURCE
F1	DEANLRR	GAC	-	CoDA
F2	QSAHLKR	GGA	-	CoDA
F3	ISHNLAR	GAT	-	CoDA

ZF DNA Sequence

Bos taurus (cow) Build 3.1

Blast GATGGAGAC

ZFA-unknown-103
1562 gGAAGCAGCTg 1572
1562 cCTTCGTCGAc 1572

FINGER	HELIX	TRIPLET	REFERENCE NUMBER	SOURCE

F1	TKQILGR	GCT	-	CoDA
F2	QSTTLKR	GCA	-	CoDA
F3	QRNNLGR	GAA	-	CoDA

ZF DNA Sequence

Bos taurus (cow) Build 3.1

Blast GAAGCAGCT

ZFA-unknown-104
1574 gGCAGCTGCTt 1564
1574 cCGTCGACGAa 1564

FINGER	HELIX	TRIPLET	REFERENCE NUMBER	SOURCE
F1	MKNTLTR	GCT	-	CoDA
F2	QRSDLTR	GCT	-	CoDA
F3	QGGTLRR	GCA	-	CoDA

ZF DNA Sequence

Bos taurus (cow) Build 3.1

Blast GCAGCTGCT

ZFA-unknown-105
1565 aGCAGCTGCCc 1575
1565 tCGTCGACGGg 1575

FINGER	HELIX	TRIPLET	REFERENCE NUMBER	SOURCE
F1	DRRTLDR	GCC	-	CoDA
F2	QRSDLTR	GCT	-	CoDA
F3	QGGTLRR	GCA	-	CoDA

ZF DNA Sequence

Bos taurus (cow) Build 3.1

Blast GCAGCTGCC

ZFA-unknown-106
1577 gGGGGCAGCTg 1567
1577 cCCCCGTCGAc 1567

FINGER	HELIX	TRIPLET	REFERENCE NUMBER	SOURCE
F1	TKQILGR	GCT	-	CoDA
F2	QSTTLKR	GCA	-	CoDA
F3	RTEHLAR	GGG	-	CoDA

ZF DNA Sequence

Bos taurus (cow) Build 3.1

Blast GGGGCAGCT

⊞ ZFA-unknown-107
1581 gGCAGGGGGCa 1571
1581 cCGTCCCCCGt 1571

FINGER	HELIX	TRIPLET	REFERENCE NUMBER	SOURCE
F1	TNSKLTR	GGC	-	CoDA
F2	RREHLVR	GGG	-	CoDA
F3	QTATLKR	GCA	-	CoDA

ZF DNA Sequence

Bos taurus (cow) Build 3.1

Blast GCAGGGGGC

⊞ ZFA-unknown-108
1584 aGTGGCAGGGg 1574
1584 tCACCGTCCCc 1574

FINGER	HELIX	TRIPLET	REFERENCE NUMBER	SOURCE
F1	RRAHLQN	GGG	-	CoDA
F2	QSTTLKR	GCA	-	CoDA
F3	RKDALHV	GTG	-	CoDA

ZF DNA Sequence

Bos taurus (cow) Build 3.1

Blast GTGGCAGGG

⊞ ZFA-unknown-109
1623 tGAAGAAGGGc 1613

1623 aCTTCTTCCCg 1613

FINGER	HELIX	TRIPLET	REFERENCE NUMBER	SOURCE
F1	KRERLDR	GGG	-	CoDA
F2	QQTNLTR	GAA	-	CoDA
F3	QTNNLNR	GAA	-	CoDA

ZF DNA Sequence

Bos taurus (cow) Build 3.1

Blast GAAGAAGGG

ZFA-unknown-110
1626 gGATGAAGAAg 1616
1626 cCTACTTCTTc 1616

FINGER	HELIX	TRIPLET	REFERENCE NUMBER	SOURCE
F1	QASNLTR	GAA	-	CoDA
F2	QQTNLTR	GAA	-	CoDA
F3	VGSNLTR	GAT	-	CoDA

ZF DNA Sequence

Bos taurus (cow) Build 3.1

Blast GATGAAGAA

ZFA-unknown-111
1629 tGAGGATGAAg 1619
1629 aCTCCTACTTc 1619

FINGER	HELIX	TRIPLET	REFERENCE NUMBER	SOURCE
F1	RKPNLLR	GAA	-	CoDA
F2	VRHNLTR	GAT	-	CoDA
F3	REDNLPR	GAG	-	CoDA

ZF DNA Sequence

Bos taurus (cow) Build 3.1

Blast GAGGATGAA

ZFA-unknown-112
1671 tGAAGAGGACa 1661
1671 aCTTCTCCTGt 1661

FINGER	HELIX	TRIPLET	REFERENCE NUMBER	SOURCE
F1	DEANLRR	GAC	-	CoDA
F2	RQDNLGR	GAG	-	CoDA
F3	QRNNLGR	GAA	-	CoDA

ZF DNA Sequence

Bos taurus (cow) Build 3.1

Blast GAAGAGGAC

ZFA-unknown-113
1695 aGCGGAAGAGa 1685
1695 tCGCCTTCTCt 1685

FINGER	HELIX	TRIPLET	REFERENCE NUMBER	SOURCE
F1	KHSNLTR	GAG	-	CoDA
F2	QQTNLTR	GAA	-	CoDA
F3	RLDMLAR	GCG	-	CoDA

ZF DNA Sequence

Bos taurus (cow) Build 3.1

Blast GCGGAAGAG

ZFA-unknown-114
1733 gGCTGTGGGCa 1743
1733 cCGACACCCGt 1743

FINGER	HELIX	TRIPLET	REFERENCE NUMBER	SOURCE
F1	SPSKLVR	GGC	-	CoDA
F2	RREVLEN	GTG	-	CoDA
F3	VGASLKR	GCT	-	CoDA

ZF DNA Sequence

Bos taurus (cow) Build 3.1

Blast GCTGTGGGC

ZFA-unknown-115
1737 gTGGGCAGTGc 1747
1737 cACCCGTCACg 1747

FINGER	HELIX	TRIPLET	REFERENCE NUMBER	SOURCE
F1	RSHILTN	GTG	-	CoDA
F2	QSTTLKR	GCA	-	CoDA
F3	RSDHLSL	TGG	-	CoDA

ZF DNA Sequence

Bos taurus (cow) Build 3.1

Blast TGGGCAGTG

ZFA-unknown-116
1755 tGAAGAGGGCa 1745
1755 aCTTCTCCCGt 1745

FINGER	HELIX	TRIPLET	REFERENCE NUMBER	SOURCE
F1	SPSKLVR	GGC	-	CoDA
F2	RQDNLGR	GAG	-	CoDA
F3	QRNNLGR	GAA	-	CoDA

ZF DNA Sequence

Bos taurus (cow) Build 3.1

Blast GAAGAGGGC

ZFA-unknown-117
1758 tGCTGAAGAGg 1748
1758 aCGACTTCTCc 1748

FINGER	HELIX	TRIPLET	REFERENCE NUMBER	SOURCE
F1	KHSNLTR	GAG	-	CoDA
F2	QQTNLTR	GAA	-	CoDA
F3	VGNSLTR	GCT	-	CoDA

ZF DNA Sequence

Bos taurus (cow) Build 3.1

Blast GCTGAAGAG

ZFA-unknown-118
1759 tTGTGGTGCCc 1769
1759 aACACCACGGg 1769

FINGER	HELIX	TRIPLET	REFERENCE NUMBER	SOURCE
F1	SKKSLTR	GCC	-	CoDA
F2	EAHHLSR	GGT	-	CoDA
F3	QPHGLAH	TGT	-	CoDA

ZF DNA Sequence

Bos taurus (cow) Build 3.1

Blast TGTGGTGCC

ZFA-unknown-119
1785 cTAGGTAGCAc 1795
1785 gATCCATCGTg 1795

FINGER	HELIX	TRIPLET	REFERENCE NUMBER	SOURCE
F1	HNGTLKR	GCA	-	CoDA
F2	QRSSLVR	GTA	-	CoDA
F3	RLDGLAG	TAG	-	CoDA

ZF DNA Sequence

Bos taurus (cow) Build 3.1

Blast TAGGTAGCA

ZFA-unknown-120
1801 cTGCGAGTGCt 1791
1801 gACGCTCACGa 1791

FINGER	HELIX	TRIPLET	REFERENCE NUMBER	SOURCE
F1	RRRNLHL	TGC	-	CoDA
F2	RQDNLGR	GAG	-	CoDA
F3	ANRTLVH	TGC	-	CoDA

ZF DNA Sequence

Bos taurus (cow) Build 3.1

Blast TGCGAGTGC

ZFA-unknown-121
1809 cTGTGTAGCCt 1819
1809 gACACATCGGa 1819

FINGER	HELIX	TRIPLET	REFERENCE NUMBER	SOURCE
F1	DGSTLRR	GCC	-	CoDA
F2	QRSSLVR	GTA	-	CoDA
F3	QPHGLAH	TGT	-	CoDA

ZF DNA Sequence

Bos taurus (cow) Build 3.1

Blast TGTGTAGCC

ZFA-unknown-122
1861 tGCTGCTAGGg 1871
1861 aCGACGATCCc 1871

FINGER	HELIX	TRIPLET	REFERENCE NUMBER	SOURCE
F1	RSSHLKM	AGG	-	CoDA
F2	QRSDLTR	GCT	-	CoDA
F3	LRASLRR	GCT	-	CoDA

ZF DNA Sequence

Bos taurus (cow) Build 3.1

Blast GCTGCTAGG

ZFA-unknown-123
1866 cTAGGGTGCCa 1876
1866 gATCCCACGGt 1876

FINGER	HELIX	TRIPLET	REFERENCE NUMBER	SOURCE
F1	SKKSLTR	GCC	-	CoDA
F2	EAHHLSR	GGT	-	CoDA
F3	RRDNLPK	TAG	-	CoDA

ZF DNA Sequence

Bos taurus (cow) Build 3.1

Blast TAGGGTGCC

ZFA-unknown-124
1898 gGGTGCAGGCc 1908
1898 cCCACGTCCGg 1908

FINGER	HELIX	TRIPLET	REFERENCE NUMBER	SOURCE
F1	VPSKLLR	GGC	-	CoDA
F2	QSTTLKR	GCA	-	CoDA
F3	VDHHLRR	GGT	-	CoDA

ZF DNA Sequence

Bos taurus (cow) Build 3.1

Blast GGTGCAGGC

ZFA-unknown-125
1926 gGGTGAGGCCt 1916
1926 cCCACTCCGGa 1916

FINGER	HELIX	TRIPLET	REFERENCE NUMBER	SOURCE
F1	VRKDLTR	GCC	-	CoDA
F2	RQDNLGR	GAG	-	CoDA
F3	VKHGLGR	GGT	-	CoDA

ZF DNA Sequence

Bos taurus (cow) Build 3.1

Blast GGTGAGGCC

ZFA-unknown-126
1946 tTGGGGAGTGg 1956
1946 aACCCCTCACc 1956

FINGER	HELIX	TRIPLET	REFERENCE NUMBER	SOURCE
F1	RNFILQR	GTG	-	CoDA
F2	QSAHLKR	GGA	-	CoDA
F3	RSDHLSL	TGG	-	CoDA

ZF DNA Sequence

Bos taurus (cow) Build 3.1

Blast TGGGGAGTG

ZFA-unknown-127
1949 gGGAGTGGCTg 1959
1949 cCCTCACCGAc 1959

FINGER	HELIX	TRIPLET	REFERENCE NUMBER	SOURCE
F1	TKPILVR	GCT	-	CoDA
F2	RREVLEN	GTG	-	CoDA
F3	QKPHLSR	GGA	-	CoDA

ZF DNA Sequence

Bos taurus (cow) Build 3.1

Blast GGAGTGGCT

ZFA-unknown-128
1952 aGTGGCTGCCc 1962
1952 tCACCGACGGg 1962

FINGER	HELIX	TRIPLET	REFERENCE NUMBER	SOURCE
F1	DRRTLDR	GCC	-	CoDA
F2	QRSDLTR	GCT	-	CoDA
F3	RPDALPR	GTG	-	CoDA

ZF DNA Sequence

Bos taurus (cow) Build 3.1

Blast GTGGCTGCC

ZFA-unknown-129
1983 gGGTGACAGGc 1973
1983 cCCACTGTCCg 1973

FINGER	HELIX	TRIPLET	REFERENCE NUMBER	SOURCE
F1	RRAHLLN	AGG	-	CoDA
F2	DRGNLTR	GAC	-	CoDA

F3	IRHHLKR	GGT	-	CoDA

ZF DNA Sequence

Bos taurus (cow) Build 3.1

Blast GGTGACAGG

ZFA-unknown-130
2016 gGGTGCAGAGt 2006
2016 cCCACGTCTCa 2006

FINGER	HELIX	TRIPLET	REFERENCE NUMBER	SOURCE
F1	KHSNLAR	GAG	-	CoDA
F2	QSTTLKR	GCA	-	CoDA
F3	VDHHLRR	GGT	-	CoDA

ZF DNA Sequence

Bos taurus (cow) Build 3.1

Blast GGTGCAGAG

ZFA-unknown-131
2032 aGCTGAAGGCt 2042
2032 tCGACTTCCGa 2042

FINGER	HELIX	TRIPLET	REFERENCE NUMBER	SOURCE
F1	TRAKLHI	GGC	-	CoDA
F2	QQTNLTR	GAA	-	CoDA
F3	VGNSLTR	GCT	-	CoDA

ZF DNA Sequence

Bos taurus (cow) Build 3.1

Blast GCTGAAGGC

ZFA-unknown-132
2054 tGTGGCCTGCa 2044
2054 aCACCGGACGt 2044

FINGER	HELIX	TRIPLET	REFERENCE NUMBER	SOURCE
F1	RGRNLEM	TGC	-	CoDA

F2	DSSVLRR	GCC	-	CoDA
F3	RHTSLTR	GTG	-	CoDA

ZF DNA Sequence

Bos taurus (cow) Build 3.1

Blast GTGGCCTGC

ZFA-unknown-133
2057 gTGTGTGGCCt 2047
2057 cACACACCGGa 2047

FINGER	HELIX	TRIPLET	REFERENCE NUMBER	SOURCE
F1	KRRDLDR	GCC	-	CoDA
F2	RREVLEN	GTG	-	CoDA
F3	QRHGLSS	TGT	-	CoDA

ZF DNA Sequence

Bos taurus (cow) Build 3.1

Blast TGTGTGGCC

ZFA-unknown-134
2060 tGTAGCCTGTc 2070
2060 aCATCGGACAg 2070

FINGER	HELIX	TRIPLET	REFERENCE NUMBER	SOURCE
F1	RKQHLTL	TGT	-	CoDA
F2	DSSVLRR	GCC	-	CoDA
F3	QSTSLQR	GTA	-	CoDA

ZF DNA Sequence

Bos taurus (cow) Build 3.1

Blast GTAGCCTGT

ZFA-unknown-135
2094 tTGGGTTTGTt 2104
2094 aACCCAAACAa 2104

FINGER	HELIX	TRIPLET	REFERENCE NUMBER	SOURCE

F1	RRQHLQY	TGT	-	CoDA
F2	HKSSLTR	GTT	-	CoDA
F3	RSDHLSL	TGG	-	CoDA

ZF DNA Sequence

Bos taurus (cow) Build 3.1

Blast TGGGTTTGT

ZFA-unknown-136
2113 aGGGGCTGAAg 2123
2113 tCCCCGACTTc 2123

FINGER	HELIX	TRIPLET	REFERENCE NUMBER	SOURCE
F1	TTTNLRR	GAA	-	CoDA
F2	QRSDLTR	GCT	-	CoDA
F3	RTEHLAR	GGG	-	CoDA

ZF DNA Sequence

Bos taurus (cow) Build 3.1

Blast GGGGCTGAA

ZFA-unknown-137
2116 gGCTGAAGAAg 2126
2116 cCGACTTCTTc 2126

FINGER	HELIX	TRIPLET	REFERENCE NUMBER	SOURCE
F1	QASNLTR	GAA	-	CoDA
F2	QQTNLTR	GAA	-	CoDA
F3	VGNSLTR	GCT	-	CoDA

ZF DNA Sequence

Bos taurus (cow) Build 3.1

Blast GCTGAAGAA

ZFA-unknown-138
2119 tGAAGAAGGCa 2129
2119 aCTTCTTCCGt 2129

FINGER	HELIX	TRIPLET	REFERENCE NUMBER	SOURCE
F1	TRAKLHI	GGC	-	CoDA
F2	QQTNLTR	GAA	-	CoDA
F3	QTNNLNR	GAA	-	CoDA

ZF DNA Sequence

Bos taurus (cow) Build 3.1

Blast GAAGAAGGC

ZFA-unknown-139
2194 aTGGGGTGGTt 2204
2194 tACCCCACCAa 2204

FINGER	HELIX	TRIPLET	REFERENCE NUMBER	SOURCE
F1	MKHHLAR	GGT	-	CoDA
F2	EAHHLSR	GGT	-	CoDA
F3	RSDHLSL	TGG	-	CoDA

ZF DNA Sequence

Bos taurus (cow) Build 3.1

Blast TGGGGTGGT

ZFA-unknown-140
2195 tGGGGTGGTTc 2205
2195 aCCCCACCAAg 2205

FINGER	HELIX	TRIPLET	REFERENCE NUMBER	SOURCE
F1	MNSVLKR	GTT	-	CoDA
F2	RREVLEN	GTG	-	CoDA
F3	RQGHLKR	GGG	-	CoDA

ZF DNA Sequence

Bos taurus (cow) Build 3.1

Blast GGGGTGGTT

ZFA-unknown-141
2221 tGGTGAGGTCc 2231

2221 aCCACTCCAGg 2231

FINGER	HELIX	TRIPLET	REFERENCE NUMBER	SOURCE
F1	NTSLLRR	GTC	-	CoDA
F2	RQDNLGR	GAG	-	CoDA
F3	VKHGLGR	GGT	-	CoDA

ZF DNA Sequence

Bos taurus (cow) Build 3.1

Blast GGTGAGGTC

ZFA-unknown-142
2233 aGCTGTTGCTg 2243
2233 tCGACAACGAc 2243

FINGER	HELIX	TRIPLET	REFERENCE NUMBER	SOURCE
F1	TKQILGR	GCT	-	CoDA
F2	HKSSLTR	GTT	-	CoDA
F3	VSNTLTR	GCT	-	CoDA

ZF DNA Sequence

Bos taurus (cow) Build 3.1

Blast GCTGTTGCT

ZFA-unknown-143
2236 tGTTGCTGTTg 2246
2236 aCAACGACAAc 2246

FINGER	HELIX	TRIPLET	REFERENCE NUMBER	SOURCE
F1	QATLLRR	GTT	-	CoDA
F2	QRSDLTR	GCT	-	CoDA
F3	IRTSLKR	GTT	-	CoDA

ZF DNA Sequence

Bos taurus (cow) Build 3.1

Blast GTTGCTGTT

ZFA-unknown-144
2239 tGCTGTTGTCa 2249
2239 aCGACAACAGt 2249

FINGER	HELIX	TRIPLET	REFERENCE NUMBER	SOURCE
F1	TKKILTV	GTC	-	CoDA
F2	HKSSLTR	GTT	-	CoDA
F3	VSNTLTR	GCT	-	CoDA

ZF DNA Sequence

Bos taurus (cow) Build 3.1

Blast GCTGTTGTC

ZFA-unknown-145
2252 tGTTGACAACa 2242
2252 aCAACTGTTGt 2242

FINGER	HELIX	TRIPLET	REFERENCE NUMBER	SOURCE
F1	GHTALRN	AAC	-	CoDA
F2	DRGNLTR	GAC	-	CoDA
F3	HHNSLTR	GTT	-	CoDA

ZF DNA Sequence

Bos taurus (cow) Build 3.1

Blast GTTGACAAC

ZFA-unknown-146
2284 aGCAGCAGGTc 2274
2284 tCGTCGTCCAg 2274

FINGER	HELIX	TRIPLET	REFERENCE NUMBER	SOURCE
F1	TTTKLAI	GGT	-	CoDA
F2	QSTTLKR	GCA	-	CoDA
F3	QPNTLTR	GCA	-	CoDA

ZF DNA Sequence

Bos taurus (cow) Build 3.1

Blast GCAGCAGGT

ZFA-unknown-147
2278 tGCTGCTGAAc 2288
2278 aCGACGACTTg 2288

FINGER	HELIX	TRIPLET	REFERENCE NUMBER	SOURCE
F1	TTTNLRR	GAA	-	CoDA
F2	QRSDLTR	GCT	-	CoDA
F3	LRASLRR	GCT	-	CoDA

ZF DNA Sequence

Bos taurus (cow) Build 3.1

Blast GCTGCTGAA

ZFA-unknown-148
2291 gGCAGAAGCCc 2301
2291 cCGTCTTCGGg 2301

FINGER	HELIX	TRIPLET	REFERENCE NUMBER	SOURCE
F1	DSPTLRR	GCC	-	CoDA
F2	QQTNLTR	GAA	-	CoDA
F3	QGNTLTR	GCA	-	CoDA

ZF DNA Sequence

Bos taurus (cow) Build 3.1

Blast GCAGAAGCC

ZFA-unknown-149
2315 cTGTGTGGCTa 2325
2315 gACACACCGAt 2325

FINGER	HELIX	TRIPLET	REFERENCE NUMBER	SOURCE
F1	TKPILVR	GCT	-	CoDA
F2	RREVLEN	GTG	-	CoDA
F3	QRHGLSS	TGT	-	CoDA

ZF DNA Sequence

Bos taurus (cow) Build 3.1

Blast TGTGTGGCT

ZFA-unknown-150
2330 gGGCGTAGCCa 2320
2330 cCCGCATCGGt 2320

FINGER	HELIX	TRIPLET	REFERENCE NUMBER	SOURCE
F1	DGSTLRR	GCC	-	CoDA
F2	QRSSLVR	GTA	-	CoDA
F3	ESGHLKR	GGC	-	CoDA

ZF DNA Sequence

Bos taurus (cow) Build 3.1

Blast GGCGTAGCC

ZFA-unknown-151
2335 aGCAGGGGCGt 2325
2335 tCGTCCCCGCa 2325

FINGER	HELIX	TRIPLET	REFERENCE NUMBER	SOURCE
F1	KRHTLTR	GCG	-	CoDA
F2	RREHLVR	GGG	-	CoDA
F3	QTATLKR	GCA	-	CoDA

ZF DNA Sequence

Bos taurus (cow) Build 3.1

Blast GCAGGGGCG

ZFA-unknown-152
2338 aGGAGCAGGGg 2328
2338 tCCTCGTCCCc 2328

FINGER	HELIX	TRIPLET	REFERENCE NUMBER	SOURCE
F1	RRAHLQN	GGG	-	CoDA
F2	QSTTLKR	GCA	-	CoDA
F3	QKPHLSR	GGA	-	CoDA

ZF DNA Sequence

Bos taurus (cow) Build 3.1

Blast GGAGCAGGG

ZFA-unknown-153
2343 gGGCGAGGAGc 2333
2343 cCCGCTCCTCg 2333

FINGER	HELIX	TRIPLET	REFERENCE NUMBER	SOURCE
F1	RQMNLDR	GAG	-	CoDA
F2	RQDNLGR	GAG	-	CoDA
F3	KNHSLNN	GGC	-	CoDA

ZF DNA Sequence

Bos taurus (cow) Build 3.1

Blast GGCGAGGAG

ZFA-unknown-154
2352 gGCAGAATAGg 2342
2352 cCGTCTTATCc 2342

FINGER	HELIX	TRIPLET	REFERENCE NUMBER	SOURCE
F1	RRRNLQI	TAG	-	CoDA
F2	QQTNLTR	GAA	-	CoDA
F3	QGNTLTR	GCA	-	CoDA

ZF DNA Sequence

Bos taurus (cow) Build 3.1

Blast GCAGAATAG

ZFA-unknown-155
2355 gGTGGCAGAAt 2345
2355 cCACCGTCTTa 2345

FINGER	HELIX	TRIPLET	REFERENCE NUMBER	SOURCE
F1	QGSNLAR	GAA	-	CoDA
F2	QSTTLKR	GCA	-	CoDA
F3	RKDALHV	GTG	-	CoDA

ZF DNA Sequence

Bos taurus (cow) Build 3.1

Blast GTGGCAGAA

ZFA-unknown-156
2367 tGCGGGTGGCc 2357
2367 aCGCCCACCGg 2357

FINGER	HELIX	TRIPLET	REFERENCE NUMBER	SOURCE
F1	VPSKLKR	GGC	-	CoDA
F2	EAHHLSR	GGT	-	CoDA
F3	RLDMLAR	GCG	-	CoDA

ZF DNA Sequence

Bos taurus (cow) Build 3.1

Blast GCGGGTGGC

ZFA-unknown-157
2370 gGGTGCGGGTg 2360
2370 cCCACGCCCAc 2360

FINGER	HELIX	TRIPLET	REFERENCE NUMBER	SOURCE
F1	TKQKLQT	GGT	-	CoDA
F2	RTDTLAR	GCG	-	CoDA
F3	HGHRLKT	GGT	-	CoDA

ZF DNA Sequence

Bos taurus (cow) Build 3.1

Blast GGTGCGGGT

ZFA-unknown-158
2373 aGAGGGTGCGg 2363
2373 tCTCCCACGCc 2363

FINGER	HELIX	TRIPLET	REFERENCE NUMBER	SOURCE
F1	RRLTLLR	GCG	-	CoDA
F2	EAHHLSR	GGT	-	CoDA
F3	RGDNLKR	GAG	-	CoDA

ZF DNA Sequence

Bos taurus (cow) Build 3.1

Blast GAGGGTGCG

ZFA-unknown-159
2389 aGGGGCAGAGa 2379
2389 tCCCCGTCTCt 2379

FINGER	HELIX	TRIPLET	REFERENCE NUMBER	SOURCE
F1	KHSNLAR	GAG	-	CoDA
F2	QSTTLKR	GCA	-	CoDA
F3	RTEHLAR	GGG	-	CoDA

ZF DNA Sequence

Bos taurus (cow) Build 3.1

Blast GGGGCAGAG

ZFA-unknown-160
2392 gGGAGGGGCAg 2382
2392 cCCTCCCCGTc 2382

FINGER	HELIX	TRIPLET	REFERENCE NUMBER	SOURCE
F1	DRSQLAR	GCA	-	CoDA
F2	RREHLVR	GGG	-	CoDA
F3	QTTHLRR	GGA	-	CoDA

ZF DNA Sequence

Bos taurus (cow) Build 3.1

Blast GGAGGGGCA

ZFA-unknown-161
2447 gGAAGAGAACt 2437
2447 cCTTCTCTTGa 2437

FINGER	HELIX	TRIPLET	REFERENCE NUMBER	SOURCE
F1	GHTALRN	AAC	-	CoDA
F2	RQDNLGR	GAG	-	CoDA

F3	QRNNLGR	GAA	–	CoDA

ZF DNA Sequence

Bos taurus (cow) Build 3.1

Blast GAAGAGAAC

ZFA-unknown-162
2450 gTGGGAAGAGa 2440
2450 cACCCTTCTCt 2440

FINGER	HELIX	TRIPLET	REFERENCE NUMBER	SOURCE
F1	KHSNLTR	GAG	–	CoDA
F2	QQTNLTR	GAA	–	CoDA
F3	RSDHLSL	TGG	–	CoDA

ZF DNA Sequence

Bos taurus (cow) Build 3.1

Blast TGGGAAGAG

ZFA-unknown-163
2459 gGTTGACAGGt 2449
2459 cCAACTGTCCa 2449

FINGER	HELIX	TRIPLET	REFERENCE NUMBER	SOURCE
F1	RRAHLLN	AGG	–	CoDA
F2	DRGNLTR	GAC	–	CoDA
F3	HHNSLTR	GTT	–	CoDA

ZF DNA Sequence

Bos taurus (cow) Build 3.1

Blast GTTGACAGG
