# Supplementary material for: Targeting wild-type Erythrocyte receptors for Plasmodium falciparum and vivax Merozoites by Zinc Finger Nucleases In- silico: Towards a Genetic Vaccine against Malaria
Source: Genet Vaccines Ther. 2012 Aug 31;10:8. doi: 10.1186/1479-0556-10-8 (PMC3500210; doi:10.1186/1479-0556-10-8)
Supplement: Additional file 2 — A list of 168 ZFAs binding to sequences within the human glycophorin-a gene. This file details the recognition domains of the 168 ZFAs that bind to nucleotide sites within the first 6, 873 bp of the human glycophorin-a gene. Overall, there were ZFAs capable of binding to sequences across the entire 31,451 bp of the human glycophorin-a gene. [file 1479-0556-10-8-S2.pdf]

Zinc Finger Site Type: Array
Zinc Finger Engineering Method: CoDA
Sequence Name : glycophorin A
Sequence Length:31,451
Nucleotide Sequence
:nGCAGGAAGGTGGGCCTGGAAGATAACAGCTAGCAGGCTAAGGTCAGACA
CTGACACTTGCAGTTGTCTTTGGTAGTTTTTTTGCACTAACTTCAGGAACCA
GCTCATGATCTCAGGATGTATGGAAAAATAATCTTTGTATTACTATTGTCAGG
TAAGTGATTTTATTTCATCTTGGTTCTGTTATATTGGGTATGAGATCATAGAA
TAAAATATGAACTACCCTATTTTAGTTCTATCTTATTTAAATCAATAAATGAGT
AGTATTTCCTCTTCCAGTCTGGTGGATGGATTTTACTGGAACTCAGCTACCA
ATGTGGGGGAAATGGCACAAGGGAGCCCAGTATTTATGGCCAAATCCAGTT
TTCTAGTATGAGAAGCTTACTTCAATTCTAAGTCTAGCTAGAATTAAAATAAT
TTTATCAAATGCTATGAGAAATACCTCTCTGTGAATAAATGTATTGCTTTGTTT
GAGTTATAAGGAGATTCATTTCCAAACTAAAGAGTTATTAACGAAGATGTTG
GTAGCTATATGGCTTTTAGTTTTCAAAAGGTATAATTTCCTATTTCTGCCAAA
TGGCGAGAAGCCAAAAGCATGAACACTGAAACCGTGGGGAGTTGTTCGCTT
CTCTGTGGGTCCATTACTAAAGTGTCACATAGGAAGAAAAAAAACAAAAACA
ACTCTTACTGGCTTAGGTATCCTGTGAATTTTAGGAGAAATTTAAATCCATTA
AAATAAAGAAATATCATAGGGTTATTATTAAATTGTATTAATTCAATAATTTGA
ATTTAACTTAGTTTAAATTTAATTATTAATTTAGTGTCTTAAATTAACATGATTT
TGGCCTCTTTCTGAGAATATTATAGTTAAACATCCTCTCAAGTGCAGTGCTTA
TGTGTTAGCAATACTAGTGCCCAGCACACAGCGGGCAGGCAGTTGCTTGAA
ACATTCTGAGTCTATTAGACATTGCTGTATCCCAAGTGAGAGCAAGTATCAA
GGAGCTACTGAGCACTCTGTAGCACACAGGGAGGAGAGATCAGCATTTTCT
AAGATACCCTAGGGGAGGATAAAATAGTGCAATAGTTAAGAGCACAGGCAT
GAGGAACAGACAGAACTGGGTTCAAATCTACTTTTACTTCTCAAGGCTGGG
GAACATTAAGGCAAATTATGTGCCCACATTTTTATGTGTCCTCGTCTTTAAAA
TGCAGGCAGTGTTGGTACTTACCTCATAATAATTGCATAAAGATTAAACAAAA
TATTTAATGGAATACACTTACTGATGCCTGAAACAAAGTAAAATGTTAAGATT
ACTATGCATTTTCTGTGATTAGAATTAACTATCATGATTAAAAAGTATTAATAA
TATATTATTAAAATAAGCAGTAGCTATCAATAGTTACAGACTAGGGAACAAAC
CTACGTATGTGATTGGTGATTTCTGAAAAGTCAGAGAGAAAAGAAAATTACA
GAAAGAAAACAGAAAACAAACATAGCTACTCTAATTTTTTAAGCAGAAAAGTA
TGAAAACATTTAGTTTGAAGAAAAGAAAACAAATGAAAGGGATGTAGTGTAA
TATTTGTATATATATTCATATATTTGAAGTGCTATTACACAGAAAAAAAGATGT
ATTCTTTGTGTTGCTCCATGGGGCAAACCAAACTGGATGTAACTCAAGCAAA
ATTAGACACTGCATACTCTACTGGGGGTGTGCCCAGCATTTGGGAAAACTC
TGTGTGACTTACAAGTGCCCCAAATTTGGAAAGGGTTCCTGGCAAAGAAAT
GATTTTTTTTTTAAATTTCTACAACTACACAAGCAGATAGTGTATTAAAGCCTT
AAATGGCACTTGGTCACTGGGGCAAGATGACCCTGAAAGCTACAATGGTCT
CCAGTACCCAAGCTGTTATCATCTTTGTAGCTTCAGAAACCCTCCAAGGAAA
CTCTCTTGATGTGGCTACTTTATAGTATAACAGAAAGGTGTAAGATCAAGTTT
TTCCCCCATACTGATTAGCTGAAGAGTAAACATGGTGAAGTCTTTTTCTTTTT
CTTTTATGTTGCTATAAAAAAAAAAGATGATTGCCTTGCTTTCTCCAGGAATCT

TAAGAATAAAGCCAATATTTCTAATTCTAAACTTACCAGAGATCTCCTTCCAA
ATGGAGAATCCATTTTTTCTAATATGACTTGATTCCCAGTCCCTGAATTCCTG
CACTCATTTGATGATTCAGTCATTACATGTCAGATTGTGAACCAGACACTGA
GCCCACAGCAGGAAGAAAAATGGGCTCCCATGGAGGATACACGGAGGGTA
GGCGCAGTGGATGATGGGAGGGAACGCAGATAATAAATGGAACAACAACTA
TCTTATTAAAATAAGATAAAAACAGTCAAAACTAATACAAAGCATATAAAACC
AGGTAAGATGATAAACATGAATGCCGAAAGCTGCTTAAGAAAAGGGTAGCA
GGGAGTTATTTTCTGAGTAGATGACATTTATGCTAAATGTGGAACAAGGAGA
CGGAGCCAACCCTGAAAATTCTGGGAAAAGAGGACAGAAGGCAGAGGGAA
GAGCAAGAGCAAAAATTCTGAAACAGCAGGTAAGTTAGTGTTTTCAAGGAAA
AGCTGGAGCTTTTATCTGAAAATCAGATTCTGAAGCTAAGAACCAATTTGAA
AATACAATACAATATCACTTCGACTAGGAAATTATGGCATAAACCAGGAGTC
TCCAAAAGCTTTTTGTGTTTACTTAAAAATTCATACAAAATTTGCATTCTAGGT
CATAATATACTAATTTAATTGGAGGAAACAAAGGCACTGGTATGATATCATCA
TGCCTACTTTATTCATCCGTGTATCCCCAGAATCTAGCACAGTTCCCGATTG
GTATTTATAGTAGCATATTGGTTGAATAAGCAAGGAAGGAGGTGAAGGGAG
GGAGAAGGAGAGAGAAGCAGAGAGGGAGAGGAAGGAAGAAAGAAAAGGAA
AAAGGGAAGGAAAGAAGAGAGGAGGGAGAGAGGGAGGGAGGCAAGAAGG
GAGAAGAGAGAAGGGAAGGGAAGAGACAGGAGGAAGGGGAGGAGGAAAG
GAAAGAGGAAATATTTGTTTTCATCTGGTTAGACACAGTGAGTGCTCCGCAT
AGACAGATCATTATTACCCTGTGCATCTGACTCATACCCCTGCAAGTACATC
AGTCTGAGAAGCACATGTTAAGTGAAGAAACAAGGCATCTCTTTTTTTTTTTT
TTTTCAGGGATCCAAGAAGAGAGCCTTGCTAGCTGCTATTTAATTGGCACAG
GAAAGAGTTACAGGAACTGTATGCCAGGGAATACATGACTATAAATTCTTTA
AAAGCAAAACCTGTGTCTTCGCTTATGTGTCCCACACATTGTCAGCCACATA
GTAGGCAGTCAATATCAACTACTCAAAATGACAAATGACAAATGACCAGAAT
TCTGCGGCAGACTAGTTTAGCCATGAAAAATCATTTAACACCCGTGGGCCTC
AGTTTTCTTGTGCCTATTCAATAAAGCGCCGAGTAGATGGTATCTACAAGCA
TTTTTCAACTGTAAACCCCAATGAATCCCCAAAATTCAGCCTGAGATGAGCT
GGACTAGTTGCCAAACCTATAAATATCTTTAGCATGGTGTGAAATAGGGTTT
TTAGAAAGAAACAGACACCCACTGTGAACTCCTTTGCAGAAAAGGTCTGAAT
AGAGGGGAAAGTAGGGATGGTATCTCAAACTTACTTTGTAGTGATTTTAAAT
TAGGAAATTTAGCTTCACATTCTTGTGATAAATTTCTTTTCACCTTGGTTTCTA
GAAGATTATTCAAAACATCTGTGAGACTATTTGAGAAGTATACTTTTGGGGA
ATTTCCCCAAGTTATCTTTATAGATTATATTTTGACATCAACTGCAAATGTAAT
ATCTTTTACTCAAAAAAAACCCAATCCTACTTACATGGTGCTGACAAAATCAG
GCTGGACCTACATTTTTACATCATAGATTTCCAGCCATTATTATCATATCCAC
ATCTTTAGTAAGTACCTATCTGTGTAGTTTTCTGTGATAAATGAACTAAACTA
AAACTAAAGCAAAAATGTTGAAAAAAAAATTCCAGGTTTATCTCTGAGTGTTGG
GATTGCAAGGTTTTTTTTTCTCATTTTAAATACTTTCTAAATTTTCTGCAAAGA
GAACCATATAATCTAATCAGGACAAGTTTTAATATATTTTAAAAAGTAAACCG
AACAAACACAATCTCTGCTTTCTAAGAAGTCTTTAATTTTTGTACGTTGGTCA
TAGACTATGACTATACAATTTATTTGTGATATGTATTAAGAATTTCTGTCTAAC
CCAAATTATTATATGTAAGCACGGGAAAAATGATGTCATCTTTGTTTGTAGTG
TACAAAGTTCTATAAACAGCTATTTGATCAACTTTGGTATTTCCATCCCTAGA
TTTATATACAGCAGGTTAGGTTCCATACAGAGGCAGGTTCTGAATAATAATA

ACCAACACTGATAATAGCACTTACTTTGTGCCGTGCACTGTTCTAAGCAATTT
ACATACACTTAATTTTTAAAATTGTAGTAAAATACACATAATATAAATTTACCA
TTTGAACCATTTTAAAGTGTACAATGGGTAGCATTTAATGCAGTCAAAATGAT
GCACACCCATCACCATTATGTAGCTCCAGAACATTTTCATCACTCCAAAAGG
AAACCTCTTACCCATTAGCAGCCACTTCCAATTCCTCCAGCCCCTGGAAACC
ACTAATTTGTTTTCTACATCTACAGATATACCCATTGTAGATATTTCATATAAA
TGGAATCATATAATAGGTAGCCTTTTGTGTATGTCCTCTTTCACTTAAAATAA
TGTGTTTAAAGTTCATCCATATTGTAGCATGTATCAGTATTTCATTCCTTTTAT
AATTGTGTTGGTATATCTCATTTTGTTTATCCACCCATCATTTGATTAAAATTT
GGGTTGGCATATCACATTTTGCTTATCGATCCATCATTTGATTAAAATTTGTG
TTGTTTCCACCTTTTGGCTATTGTGAATAGTGCTGCTATAAATATTCCTGTAC
TAGTTTTGTTTGAACCCACTTTTAATACTCAAAGATGTATAGGGGTAGAATTG
CTGGGTCATAGTAATTTTATGTTTAACTTACTAAGGAACTGCTCAACTCTTTT
CCACAGGAGCTGCACCTTTTGACCTTTTCACCAGGGTGTATGAGGTGCCAA
TTTCTCCACAATCTTGCCAGAAATTGTACTTTTTCATTTTTTTAATTATAGCCA
TTTCAGAGGGTATGAAATGGTTTTTCACTGTGGTTTCTTGCATTTTCCTAATA
ACTAATGACGCTGAGAATCTTCTCATGTAATTGTTGGTAACTGCATTTTGCAT
ATCTTTGGAGAAATGTTGGTACTAGTCCTTCACCCATTTTTCAATCTATTTTT
CTTTTTGTGTTGCTAAGTTGTAAGAGTTCTTTCTATGTTCTGGATAAAGAGTC
TTATCAGATATACTATTTGCAAATCTTTTCCTTCATTCTGTAGATTTTTGTTTTT
ACTTTTGATAGTGTCCTTTGATGCACAAATGTTTTTCATTTTCAAGTCCAATTT
ATTTTTTTTTCTTTTGCTGCTTACGCTTTTGATATCATATCTAAAAATAATTGC
CAAATTTAAAGTCATAAAAATTTCTCCCTATGTTTTCTTCTAAGAGTTTTGTAT
TTCTTCTCTTATATTTAGATCTTTGGTTTATTATCAGTTAATTTTTCTATATGAT
GTATGATAAGAGTCCACCTTTATTATTTTGCAGCTGTCCCAGCACCATTTGTT
GAAGAGACTATCCTTTGCCCATTGAATGGTCTTGACACCCTTCTTGAAAGTT
AATTGGCCATGGATATATGAGTTTATTTCTGGAGTCTCAATTCTATCCTAAGA
ATATGTCTGTTCTTGGGGCAAAATCACACAGTTTTTATTGCTGTTACTTGGTT
ATACGTTTTTAATTCATGAAGTGTGATTCACCAAACTTTGTTCTTCAAGATTG
TTTTGCCTATTTAGATCCCTAACAATTTCATAGAAATTTTAGGATTAGGTTTTC
CATTCTTGCAAAAAAATAATTATGTGCATTTTAACTTAACCTGTTCAATAACTC
TATAAGGTAGAGACTAATCCATGTATAATGATGGAACAAAAATATAGAGATTA
AGTAAATTTTGCAAGGTCTCAGGTAGTTGCTAGAGGAATTAGTTTGAGCCTA
GGCAGTTCCACTGCAGAATCTGTGCACTTAGAGAATATGTCATGTTGCCTGT
ACCATACCTAGTGATGTTCCAGGATTGGCTCCTTTACTCTTACAACATTGTCA
CTCAGTGTTCTGCCTGTGCTTTCACCAAGCTGAAGACTTTAATGAAGGTTGA
CGGTCTGTCTTCCTCACGTGGTGCAGCTAAGGAACTCTAACTGTGTGGCTG
TTATGTTAGCCTTTTGCTCCTTTTTATATGGGCTATAGAAAATGTTTTTAAATC
CTGGAGGCCTCCTTTTGATGTTATCACTTATTTCCCAGTCATCACTATATTTT
TAAAAGCCAAAATAGAAGGAAATAAATACAAAACATAAAACATGAATAGTACA
GCTATTTGAGGCAACTGAGAATAGAGATCATGGCACTGAAATTGCATTTTGC
TAGGAAAAAGACCACAAAAGTTCTCCCCTTGCTACCTTTCCTGAACTATTCT
GCTAGATTCAGACTTCAAAAACATTGTATCAGGAAATACAGAAATGTTCTTTC
AAAATGAGTGTATGGGAATGTGGGAATGCCTAATAAAATCTGTCCTCATTGA
TTCGTTAGCAAAAATCATATAAATCAATACCTTGTGATTGCAAGCAGATATAT
TTCAGATCCTTTCTGTGTTTGTTTTTTTGCTTTCTTGATCTATCACAATTGGAG

AAAACTTAAAATTTCTCAATGGTATTGTATTTTTGCCAATTTCTTATTCTGCTT
TATGTTTCTCGTTGCTATATTATTGGGCTATAATGGTCCATAATTACTTAAGA
ATCACTGTGAAATATATTGCTTAATGACACAAGTAAATCTTTTTCATTGTTTGT
AATGTCTTTGCTCTTAATTCTACTTTGCCTAAGATTAATACGGTTATTCCTGTT
TAGTTTTATATGTATTTATTTATTTATTTTGAAGATGGAGTCTCGTTCTGTCGC
CCAGGCTGGAGTGCAGTTGCATGATCTCGGCTCACTGCAACCTCTGCCTCC
CGGGTTCAAGCAATTCTCCTGCCTCAGCCTCCCAAGTAGCTGAGAATACAG
GCGCACACCATCGCGCCCAGTTAATTTTTTGTATTTTAGTAGAGACGGGGTT
TCACTGTGTTGCCCATGCTGGTCTCCAACTCCTGAGCTCAGGCAATCCACC
TGCCTCGGCCTCCCAAAGTGTTGGGATTACAGGCATGAGCCATTGCACCAG
TCCTAACCTATCTCTTTTGACTCAATCTAAAAGTTTCTGTCTTTTAATACAAAA
CCACAATCCATATGCATTCATTAATTCACAACTGACATTTAGTATCTTATTTCT
GTTATCCTATTTCATATTTTATGATTCCTTGTTTCTGCTCTTTTGATATATAAAT
TATGTTTTATTTGCCCTTATCCTTTCATGTGTTTCTAAAGTATATAGCCTACGT
GTAATTGTCCCATTAGCTAACTTTATGTTTTTGAAAGCATTCTCTCTCAGAAT
TCCCATTTTAGTGGTGCAGCACACATAGAAAGTCTAAGTGCTTTCTGGAGCT
AGATAAGCTGGATAAAGGTGTGCATGAGCCACTGGTCAATGGCTTGTGCAG
GCGGTGAGTGCATTTCTGGTATTTCATATGCTATTGATCTGGCAGCCAGGTA
TTCAGATAGGGTATAACCAGGTTCATCAGGCTCAAAACATAATCAAGTATTA
TTGAGACATAGTTAATGTGCACTACAACTCACAGCACACAGGCTCACACACA
CACTTGTCTGAAATAAAATTCCACAAAATAATACCTTCCCTTATTCTGTGTGA
TGTACTTTGATATATTCTCTCCTGTTTTATACAACTTAATTTTTTTTAGAGAAA
AGATTTTGCTCTGTGGCCTAAGCTGGACTGCAACGGCACAGTCATAGCTTA
CTTCAGTCTTGAACTGCTGGATTCAAGTGATTCTCCAGCTTCTGCCTCTCAA
GTAGCTGAGACTTCAGGTGTGCTCAACCACACCTGACTAATTTTTTGGTTAT
TTAATTTGTAAATATGGGGTCTTGCTATGTTGCCCAGGCTGGTCTCGAGCTC
CTGGCCTCAAGCGATCCTCCTGCCTTGGCCTCCCAAAGCACTGGGGTTACA
GGCATGAGCCACCACACCTAGAATACAACTTAATTTTTTAGTGCCAGTGACA
ACCCACTGGACTGATTTCATAACCCATTAGTAGAGGAATGCACCATCTTGAC
TGAAGGTTGGAATTTTCTCAGGGAATCTATGTAGCACTGATGATTGGGTTTC
ATATCCAGAGATTCTAGTTATGCTAATACAGAGGCCAAGCAAACTATAGCCT
GTGAATGGCCGGCCCCCTGGTTTTGTATACCTTACAAGTTACAAATGATTTT
TACTTTTTTAAGTGCTTAAAAAAACCAAAATAGGCCGGGTGCAGTGGTTCAA
GCCTGTAATCCCATCACTTTGGGAGGCTGAGGCAGGCGGATCACGAGGTC
AGAGGATCGAGATCGTCCTGGCTAACACAGTGAAACCCCATCTCTCCTAAA
AATACAAAAAATTAGCCAGGCTTGGTGGTGGGCGCCTGTAGTCCTAGCTAC
TTGGGAGGCTGAGGCAGGAGAATGGAGTGAACCCGGGAGGCAGAGCTTGC
AGTGAGCCAAGATCATGCCACTTCACTCTAGCCTGGGCAACAGAGCAAGCC
TCTGTCTCAAAGAAAAAAAAAAAAGAAAGACACAAAAAAAATCAAAATAATAAT
AATAATATGTGAATATTATATGAAATTCAAATTCTACTGCCCACAAATCATTAT
TGGAACATAGTCATACTCATTTATTTATGCTTTGGTTTACATATTGTCTGTAG
CTGCTTTTGCACAGTGACAGAGTTGAATATTTGTAATAGATGGTCCACAAAG
CCTAAAGTAGTTGTGGCCCACAAATCCTAAAGTAGTTACTCTCTCTCCCTTTA
CATAGGAAGTTTACTAATACTTGTGCTAAGGGATCTCAACAGACAATCTGAA
AAACTTAAGTTTTAGACTAAAGATTTCCAATCTAAATTCCTGTGGAGCTTTCT
GAAGCTGCCAGGTGGAGATGGGAACAGGTTGTGAGGCTGCAGGCCAAACA

CTCAGGCCAGCTTCCACCAAGCAGTTCAACTCTGTCTGTTTCACACACTGAT
GAGCTTATCCTTGGAAAGTGATTAAAGTAAAATTAAATGCGAATTGAGGGAG
GAAGTGAGGGAGACTGTGGCTCTAAAACAAAACCCTAAGAAACACCAACAT
TTAAGATGGCAAATGATGTTATTTCTAAAGTCGTTCAGGCTAATATCACATAC
TATAGCTGTTCACTTTATAGATAAAGGTGACACTACAACCATAGAAAATGTAA
GAGTGGACCTCGAAACTCAGGAAGATGAAGTTTACATATATTAATCTATATTA
CCAACTGGAGCAGTTGTTCTCACTGCTGGCCGCACATCAGAATCCAATTCCT
GGGATATCACAGATGATTCTACCATGCAGTCAAGGATGAGAACAAACTAGG
TTCATTTCTGCAATTTTTTTATTGTTCAACCAGTGAAAAGGAAGTACCAGTGG
TGTGAGAACTTTGGGATAAAGTTTTTGTTTTCAATTAAAATTATTTTCATCCAG
CCCAACTTCCTTAAGCCCAAATTTAATGTGTGTGAAGTTCAGCTACAGAAAT
ACCAAACCTTAGACTAAAGCGGACACAGGTAAAATATGTGAAATCCTCTTTT
GTTCTGAGGATTCTTTAGTAGGCAGGAGTGACCAGATAGGAATATGCTTGG
CTGGAAAAATTAAGATTCAAGTTAACAAACTGTTAATAACCAGGACCATCTG
CTCTTCCGTAATGTGGATTTGCCACTGCAGGTCACCCTACAATGCTATGTTA
GAGGTACAACACTCTTACCCTCAGGCTATAAACAAGGTGAATTATTATCTTTA
TATCTCTTCATTTAGCCCTGATTTGCTGAAGTGAAGGCTCGCTTGAGAGTTG
GTTGCATTATAATTTGGTGAGAATTTAATCTCTCAATGACAACTTACTTGATT
CCCTCATTCTCTTTCTGCTACATAGATCACAGTAGACCTTGGCAGACAGTTC
TGTAGTTACATAGGTCTGAATTCAAAATCCAGGTCTGCCACTTGGTGGCTGT
GTGAACTTAAGCAAGTCAGGCAATGCTTCTGATGTTTTTTTCCTCCTCCACA
AAGAATAATTAACATATAACAATAGGGTCTCAGCTAGTTGTTTTAAAAATGGT
TAGAGAGATGTGTGGAATGAAGTAAGTGTGCAGTAAGTGTTAACTACAAATA
TTATTATCTTAGACATACAGATTTCCATGATTCATGAATGGTGAAGCATCTTA
GAAGACATCCATTCCAGGCCAGGCATGGTGGTGTGCACCTATAGTCCAAGT
TGCTCAGTAGAATGAGGCAGGAGAATTGCTTGAGCCTAGGAGTTTGAGGCT
AGTATGGGCAATATGGTGAAACCCTATCTCAAGAAAAAAGCAAAACATTTTT
TAAAGTTTAAAAAGAGAGACATCTGTTCCACTACTCTCATCTTAGAGGCCAT
AAAACTGAGGCTCAGATAATTTCAGAGACTTGCACAGATCCCCCAACCATTT
GGTGGCAAAGCCAGGAAGAGAACTCTGCTCTCCTTTCCCACTGGGACAGTG
GAAGAAATTCGTCTTGATTTCCATCTGTCCAGGCTGAAGAATGTGCACTGGC
TGGAATGACAGACTGACCGACTTTTTTTCTCCACCTCTGCTGTCTCAGCAAT
GGTTTGGGACAGTGTGGATGACCAGAAGCTGGATAGTACAGAGCCAGGCT
AAAGAGTTCAGGCTTCCTGAAGGGAAGCTGCAGTCCTCCTAGGCCACAACA
CCTTCGAGATAGAATACATAAAGCACCCTTCTCTACCAAGTTAGGAAAGGAA
GAAGTGTGACCAATTAGCTGTATGGGGACTGCCAAAGCATGCCAGTCTGAA
GATGAGCAGAAACTGGCTCATTCCATTTGGCACCTAGCACACTAACTGCATC
CGTTAATAGGCCATGCTTTTCTCCAGAGCCATTGGCTGAAGAGATCAAATAA
AAAGTATTGAGAATAGGCTACCCAAAACAGTAGGCTCAGATGCTATCACACA
AAGCACTTTATCCTTAAGTTCAATTTTTCTAAATTGTAGTTGGCTGCTTTGGC
TTAATAAAAACTTCCAAAAAAGAAAAACGAATGGCCACAGACAGTATGGGTA
TCTAACTATATTATCACAACTTGACCAAGATTGAACTTGCCAATCCTTTGGTT
CAAGAGCCAAACAAAATCGTTCCCTTAAAATATTGCTTCATGGGAACAGTCT
TCTTCAAACATCTTTTAGCACAGGCAAGATTCCCATTTATACATTAATTCTGT
TCAAGACAATGAGATTGGGCAGAAAAGGCATTGAGTTGGAAGTCAATGGAT
ATGAGTTTTTATCCCAGTTTTACCACAAATTAGCTGAGCATAACTTCCACAGA

TGCATTTATCAAGTAGTTTTCATGGTCATTGCAATGCCAAAAAACTGTAGCAT
TTAGAAAATTTAGTTTTCAGACTTGGAAACTATTTAAGGCATTTCATATGAAG
GGTGTGTCCTTGTGAGAGTTTGCTTATGCAAGATAAGGCTTCTTTCAGCTGC
AAGTCAGGAGCGAACCAAAACTCAAAGCAGCAGCTGCATGAGCTGACTTTA
TCACATCTTGACAAGAGCTCAGCCACTGGAAGTTTTGGCATACAGCGAAACT
GAAGCGTACTTATACAATATCACATTTTATTTTTATTGTTTCTAATAGCATTCC
AGGTTAGAAATGTCAATTATTTGGGAAAGCTGAGGGTCTGGTAGATAAAGCA
TGCAGCAGAGAGCTAGGAGGCTGGCTATTTCCAGTCGTTATCCTAACATGT
CTTGGGCCCCCAAGTCACCCCACCTCCATGGTACAATGGGAACTGTGGCAG
AAGTCCACGCTCTCTCCCCCAACACATGGGGATAAGAGACAAGAGAGGTGA
AATGTTCTGGAACATATCCGATGTTATACAAGTATAAGCTGTGAGATGATCC
AAACGCAAATATTGAATATTTCATTTTCTAGAAAGTATACCAATTCATTCCAC
CCTTCTCAAACCTAAATTACAGAATTCAATTCAGGTCACACAGATTTACTTTG
TACTAAGTACCATAGCAAATGCCATTTCAGTGCCTGAAAACTGAAAAACATA
AATTTAAAGTAGGAGTTTGAGGCCTCACTAATATGACAAAACATACCTTTATA
TTTTATTTTGCAGTAATTTGCCACTTAATCATTAAACTCTTATCAATCTGAGAG
ATTTGCCAACACTTGCCTGCTAGGTGACCTAAGCCTCCACATCAATGCATGT
TATACTCCCCTTTCTCCATATGTTAGGCCCATGCTATTTCTTTATCCCTCCTC
CTCTGCATCTTCACCTAAAACTCTGCCCATCCTTCAGGGTTCATCCAGTGAT
TCATTTGCAAGCAGGCATGGGGTAAGGTCTTCAGAGTATGTTTCTCAGAGG
CCCATGCAGCTAAGAAAATGTGCAGTGTTGGCACAAGGTCTGTCTATTCCT
GGGTAGCCAGATGCTGGACACATCTTTCATAACACCACAAGGTAAATATACT
TCACTTGGAGAGAGAGGTGAAATTTTGCAGGTATAGACTGGATGTGTTCCT
GCCAGAAGATGTGAAGGGATTAAGAAACTGACTCTCATCTCCGTATTGCTAG
AGCAAAACATAATTTCTCATAGTGGCTATAGTATAAGGACACTGAGGGGTAA
GAGATATAATCTAAGTAATACAATAAATTAGTGTGGAAAAATCATCAAAATGA
AGACTACATGGTTTTTACTAAAATTCTAGCTTTTAGGATGTCCAGGGAGCTC
AGGAATTTAGCTGTCCTTTTTTGTATGTACAATATGCCCCAATGCTTGCTGAC
TAATGTACTAAAACATTAGAGAAATCTTGCTGACAAGATCTCAACCAGTCAG
CGAGATCCGGAAGGTGAGACTAATATTGAGGGTCAGCAGAATTAAGTCTCA
GTTCTGCTGCTTACCAGATATGCTGATCTGAGCTAGTCATTTAATTTTTATGA
GACCAAATGTCTATCTGTAAAGTCGGCAATTTGGATTAGATGTGCTGCAAGT
GGTTTTCTAGCTTAAATGTACCTTCTGAATTCAACAGGACAATACTTAAACTG
ACCTTTAATCTAGGAATGACACAAGTAGATTTTTGAAAGCTACTTTAGCTACA
GAAAGCTGAGAGCACCAAAGGCAAAGAGATAAAAATAACAGGAGAGCCTTC
CCTTAATCCAGTCCCTAAGCAGTTTTGGCAAACTAAAGTTTGTTGTTCAATG
GTTACGAGTTTGCTTCAATGCTTTCTACCCAGTTTACTGAACTAAATAGTATA
TAGCTATAGTAAAAAGTCCTATTCAAAAACCAGCTTCTCACAGATATTTTGCA
GCTTTGCAGAATTGAATATGTCCACAGACGTCTATTAGCTGGTTAGGGTCTT
AGGAATCTAGGAGAGCCAAGTAGTTGTGTGAGCTGTTGTTATCAAATGTAGT
TTTGAACATTCTTGGTGATTTTAAGGGATCATATTGTGGAAATTTGGTTTCCT
TACCTTGAATTTTGAATGAAGCTTTAGAATTTGAGGATGTTTCTTTGGTTTCT
CCTTCCAGGTAAGTGATTTTTTTTTTTTTCAACCAGATGCTGGTTTATTTAATT
TGAAGGTATTGATGAAATTCTTTAAATTGCCCCCATGTGATTCTACTCTGGAA
TAACTACGAAATTATTTAAAAGTTAATTAATACAAGAAAATATGAAAACTCATT
TTTATGGGAGCTATTGTTCCTTCAAGATGACACTGTTTTGTAAACTATAGACT

TCCAGTAACAAGCCTCTGTGCCTTCTTCTTACCACTAAGCATGCATGGGTAT
TAATTCCTACTGAAAGACTTATGCTATCTTTTTTCCAGAAATGGAAGAAAAAT
GAACTATGAAAAAGGTCATTTTATAGGTCAGCTACCACTATGAGATTGTTGA
GGAAATGATATAAAAAACAATTTTTATCAAATTATCTTTAGGGCATTTATATGT
TTATTTTCTTACTATGTTGACTTAGGTGACTATAAGAAGTTGTATCAGAGCAA
CTGATTCTGGTGAATTAAAGCAAGTATTTCTAAGAACATAAGTGGCAACTTTC
AGTCTCAAATCAATTTGGCCACCAATCAGTTTTTGTAAGGGTACAAATAGGA
CATAACATGCTCAGATGGGACTTGGATAAAGTGTATACAATTTTACATCGAG
GAAATTGTGTCAATGTGTTACCTTCAATGTTAGAAATTCCCAAGTTCTGACAA
TAGTTCAGAGCCTTGTTAAAAGCCAGAGTGGAGGCATGTAGATCCAGCTGG
AAAGAGAGGCATTATGGTCTAACTTAGGACAAATTTTAAAGCCAGTGTTAGG
GTCTGAGTCCAGCTTTGTAAACTTGAGTACAGTGTTTGATCTCTGGGGTTTC
AGCCTTCACTTCAGAACAAAATTTCCACCAAGTGCTCTTTTACTGTGAGGAG
TAGCTGTTGAAGAAGAAAGAAGTCTACTTATTTGCTAGAGTGTTACAATTGTT
TTGATAAAGCTCAAAACTTATCTAAATAAGCTCTCTCTCCCTAAGCATGTTTT
CATTTTTATAAAAAAGTTACATATACTTTGCTTATAAATTTAAAATACTTTTCAC
CTCCTCTGACTTCATTTAAAATTAAAATAATTAAAGTGCCAATTTTAAGAGAT
GTTAGCTCCCATTATTGGTTCTTTGCCATATTCTTTTGACAACCTGCTGTAAT
TTTCTGCCCCCTTTAAAGCCTCAGGCTATAGGCCTTCTCCACCAAAGGAATA
TTAAGAAGTGATAAGGACCTTCTGTGAGCAGAAGTGGCTTGTTTGCAAAGG
GACTGCTTATCTTGGCCACTCTTGAACACAAGATGGGACCCTCTACTGCAAA
GCTCTGGCATGTTTTTTTTTCCCCTAAGTTATCCTCCATACTACTGACAGTGA
TTTTCCCTAAATAAAAAACTGCTTCAAACCATTCATTGTCTTTCCACTGCCTT
AAAGATAAAGTCCAAATTCTAGAACATGGCCCACAGCATTTGGTGCCTCACC
ACCTCTTCAGCCTCTCAGTTGCTGTTCACCCATTTCTCTATTCCTCTCCTTCT
CACACCTTGTGCTGCAGCCACATAGATAACCTGCAGTTTTTGTAACGTGCAA
TGATGTCTCAAATTCCAAGGCATTGCTGGTACCACACAGCCTGCCTGGTAAA
ATCCTAGACTTCTTTCAAGATAAATTCAAAGACACCTCCATGAGGTCTTTCTA
CCTCTCCAAGTAGAGTTGACCGCTGTCTCCTTTGTGTCCCCACTTCCACCAC
CATCCTAAAATACTTATTATACTTAGATTAATAATTGTCGCTCTTACTGCACTG
GAATTACCCTGAAAGGAAAGGCCATGTATTATTTATCATTGTCTTCCTAGTAC
ATAGCCCACAGCCTATACCTCCCACCCCAAAAAAAACCTTTTGTAAATAATT
GAACAAATTAAGAAACACCCAAGGCCCCCAGTAAACATCAAGGCCTAAGGA
ATGCATATCTGGATTCTAAATAATCATAAGGTTTTACAACACCATGTTAAGCA
CCAGGGACTTCAGAGAGCTTTTAGTCTAAATCTTATTAGAGAGGCCAGCGAA
GACCTCCCAAAGGAAGTGGCATTGAACTGAGACTTGAAAAGCCAGTAGTTA
GGCAAAGATAGGGAGGGAAATATTTCAGACGAAGGGAGGAGATGGCACAA
GATTTAGGACACGGAAAAGGGTATGGTGCAGTCATAGAGAAAACAGATGTG
CAGAATGGCTGGAGCCCCAAGAGGGAAGGGAAGGGCGAAGCAATGAAGAT
GTGAGGCAAGCAGGACTGGACCATGCAGAGTCTTGCAGATGTTCACAAAGA
AAATTGCAGCAGGTAGTCCCTAACATCGTGCTGAACAGTTAGGCAACTTGG
AGGAATATGTATATTTGTACTCATAGTCAAAACCACTAGATGGCATTTACAGA
CTACGTTTTGTGTATTTTTATTTTTTACTTTTTGTTTTTTTTTTCTTATGTTAGC
AAAAGTATGCTCGCTATTGAAATGTTGAAAATATTTCATTGGTCTTAAAATGA
TGCTTATTTTTCCAGATGCTTGCATTCATTCTGCATGTGCTATTTTGTCATGT
GGTTTGCTTAATTTATTAAACAATTGTATTAATTAAATATATTAATTATAAATTG

ATTAATTTATAATTAATTATGTGTTATAATTAAGTTAAATTTATTAATTACTTAA
ATTATTATATTCACATTCAGATGCAATCTGAAAACCCATTTGTTCTCACACTG
CTATAAAGAAATAACTGATACTGGGTAATTTATAAAGAAAAGAGGTTCCATTT
GACCCAGCCATCCCATTACTGGGTATATACCCAAAGGACTATAAATCATGCT
GCTATAAAGACACATGGACGTGTATGTTTATTGCGGCACTATTCATAATATC
AAAGACTTGGAACCAATCCAAATGTCCAACAATGATAGACTGGATTAAGAAA
ATGTGGCAAATATACACCATGGAATACTATGCAGCCATAAAAAATGATGAGT
TCATGTCCTTTGTAGGAACAGGGATGAAATTGGAAATCATCATTCTCAGTAA
ACTGTCGCAAGAACAAAAAACCAAACACCGCATATTCTCACTCATAGGTGGG
AATTGAACAGTGAGAACACATGGACACAGGAAGGGGAACATCACACTCTGG
AGACTGTTGTGGGGTGGGGGGAGGGGGGAGGGATAGCATTAGGAGATATA
CCTAATGCTAAATGACGAGTTAATGGGTGCAGCACACCAGCATGGCACATG
TATACATATGTAACTAACCTGCACATTGTGCACAGGTACCCAAAAACTTAAA
GTATAATAATAATAAAATAAAATAAAATAAAATAAAATAAAATAAAATAAAATA
AAATAAAATAAAAGAGGTTTAATTGCCTCATGGTTCTGCAGGCTATACAAGA
AGCATAGTGCTTCTGCTTCTGGGGAGGCCTCAGGAAACAATCATGGCAAAA
GACGAAGGGAAAGTAGGCACGTCTTACATGGTTGGAACAAGAGCAAGAGA
GAGAGTGGGGAGAGAGAGCCTTGGAGCAGGAGCAAGAGAGAGTGGGGAG
GTGCCACACACTTTTAAACAACCAGATCTTATGAGAAATCACTATCTCCCAG
ACAGCATCAAGGGGGATGATGTTAAGCAATGAGAAACCAGCCCCATGATTC
AATTACCACCCACCAGTCCCCACTTCCAACATTGGGGATTACATTTCCCCAT
GAGATTTGGATGATGCCACAGATCCAAACCATACCACTCACCTAATTCTTTC
TACGTAAGAATTTGTCCAAGCATTTATAACAATTAGCATTTCATTTAACATCTT
TTATGAATAAAGCACTATTCTCATGCTGAGAAGATTCAAAATAATGGGAAATT
GAAGTCCTAGGAACAAGTTTTATGTTTCAGAAGAGCCCATTTGGTATCCACA
GGGCTAAGAAATGTGCACCCTAAATGTAAGTGGATTACACTGAACTGAAAG
GTGTAAAGAAGGAGTGGAAGATTAAAGGGAGAAGCTTGGAGAGGATGAAAG
TTAGAAATGGAAGTGACGAGCACACCTGAGTGAAGGATGAGAGCTCCAGCT
GCATTTTCCAGTTGTATTCCCATGTTGCTGAGCCAAAGGCTGATCTCAAGTT
TATTGTTACATGCCCATTTAAGGCTTCTGGCCATTAACACTTTTGATTTTTTTT
GGCTTGTTGTTTTACTAGCTATTTTCACAACACTTTCATAGCTAAACCTATTTT
ACTCAGATTGTATGCCTTTTCAAAAATACAATAGAAGGTCCATATTCCATTAT
CTAGAAATAAGCCAAAGCTCATATCTAACATTTATTAAGAGAGATGGATTATT
TTTGTTCATTAGTTATCTTTATAAATAATTTTTACGTACTTTAGTTGACTCATAA
AGATGTTTCTTTCTGTAATTTTAATCTTAATATTTGTTGAACTTCAAAATCCCT
ATCACCAGGTTATTGTTTAAAAGCATTGGTTTTTATATTATCTTAAAAGCCATT
ATACCTGAGTGCTGAACAACTTAGAAACATTCAGTAATTGTTTTGCATGCTAT
TTAGTGAATTCATATGGCAATCGTTTATACATACATGATGGAATCAGGTGGC
AGGCCAAGTTAAAGAGCAAGGCCAGAAAAGAACTTAAAAGAGAAGAGAAAA
AATAGACAGTTTAGGAACAATAGATCATGTCTTCTCCATGATTTGGAGGTAA
ACTGATTACCTATCAGCTGATAAATAGAGGAAGGTTTTAGAAGTCTTCAGTT
GGGTAGACTAATGAGAGGTGTCAGAGAAGATGTTTTCTGTTGTTTGTGGGTT
CTCCAGGAAACTTTGAGCATTCAGCTGAGGGGCCAAGTTGGCTGCCTCTGA
GAAGAAGCCCTTCCACCTCCACTCCATTGCACTTGGGTGCCATTCCCCTCA
GTTGAATATCTCCAAGAGATGAGCAAATGTACATCTACAGAGTTCAGGGTAC
TGACTTTTATCATAATGATTTATAACTCTCAGAAGAGTGAAAAACACATGAAT

GCACAGAATAGGAGATTGAAATATAAACCACAGAACATTCATACAATGGAAT
ACTCTGCAGTCATAAAAATCTTCTCATAGAAGAATATTTGACAGCATAGGGA
TATCTGTGGCATATTAAGTAGAAAGTCAGACTTGTAAACATTATATACATATT
CACGTATATTTAAACACCATGATCCCATATTTAGATATAACAACTAAAAGTTC
AGATGGCTATATATCAAAATGTGTCAAATGTTCAACCTTGCATAGGCTGACT
GTAGATGAATTTTATATTATTCTTTGTGCTTTCTTGTAGTTCCCAAATTTTCTT
TACTGAATCTATATTACTTTTGCAATTTAAAGAATTTAATTTATAAAATTTTATA
AAATAACTTATAAATTTGAAATGTATTGCATTTAAGAATAAAAAGTGTTTAATT
ACAAAAATAATTCACAATTTATTTAATGAGATTTTAAAAGGATATATGTGAGTC
TACATTCTGATTTCATGTTTGCATGCATGGTTTTTTTTTTCTTTTGAGACAGAG
TCTCGCTCTGTCGCCCAGGCTGGAGTGCAGTGGCGTGATCTCGGCTCACT
GCAAGCTTTGCCTCCTGGGTTCACACAATGTAATAGTGTTTTATTATTGTTTC
CATTTTTATTGAAGAAGTAAGATTGTCCCTAGCAGATGGAGACACTGAGATA
TGGGACAGAAGTTTTGTTCTATATAATTATTATGCGCTTCCACCTTTCTTAGC
ATAGACAGTTTCCAAAATGCAACTTCAAGTTACCCCTTTATAAGCATAATAAC
AATAATACCCAACATATATGTAATGCTCTTTATGTGCCAAGTACTATACTAAC
ACATGCACATTACATACACACACACCACATACACACACATATTTAAACTAATT
TCGTTCTCACAATGACATTTTGAGGCAAGTATTATTATTGTACAGATGAGAAA
ACCAAGGCACGCTTTATCTGTAAACCTCTGCTATGCAGAAATTCTGGAGGG
GCTTCTGGCCCCTTAATTTTAAAATAAGGCCAATAATACAATACTTACCACAT
AGCAATTCTCTAAACATTATGTAAGATATATACCAAAGCGCTTAGCTCAGGG
ACTGGAGGGATGTGAGGGAATTTGTCTTTTGCAATATGCTTTATGGTCCGCT
CAGTCACCTCGTTCTTAATCCCTTTCTCAACTTCTATTTTATACAGAAATTGT
GAGCATATCAGCATTAAGTACCACTGAGGTGGCAATGCACACTTCAACTTCT
TCTTCAGTCACAAAGAGTTACATCTCATCACAGACAAATGGTTTGTTTTCATT
TTTATTTTTAAATTGTGGCTCCGAAATCATTTTTGTGATGTAACCCATTTTAGG
GGACCTGTCACTGCAGAGAAACTGACAAACACTGAGAAATGCGAGCTAAGT
AGACACAGCCTACTAAGTAGACACAATTCCTACTATGGAGGAATTCTTGCCT
CTGAAATATCTCACAGAAATAATACTGTGAGTTAAAGAAATTAAAACAATGTG
GCAAAGCACAGAAATGATGCACGTGACCATGAAATAGTGGGCCAGATAAAG
GGGACCTAATAGTGCGGTGGTGCGGAGGGTCTGTGGGCAAACTGAGTTCA
GCTCAGACCCGGGCTCAGCTCTATGCCAGCTGCTGACCCAGGGTGAGTTG
CCCTGCAGGGTTTCTATCCCATTAATTTTAAAATGGGGCCAATAACACAGTA
CTTATCTCACAGCATTTCTCTAAAGGCTAAATAAGAAGATGTATCTAAAAGTT
ATTAGCTCAGAGCCTCACACATTCTCAGTGACTGATAAACAATAAGCAAAGC
TGGGTGCTGAGATAAGAGTAATCTGGTGGCAGTCTCTCTTGTTAGTTTTCAG
GGGAGAAGAAGAAATTCTGGAGCCGCTGCTGGGAGGGATGTGGGAGAGTT
TGTCTTTCATAATACGCTCTATGTCCACGCAGTCACCTCATTCTTGTGCCCTT
TCTCAACTTCTCTTATATGCAGATACGCACAAACGGGACACATATGCAGCCA
CTCCTAGAGCTCATGAAGTTTCAGAAATTTCTGTTAGAACTGTTTACCCTCCA
GAAGAGGAAACCGGTATGTTCTTAGTTTTAAATAGTTGCTCTGGAGTCATTG
TTGTGATTGAACTCTATTTACACGAGCTGTAACTCATGACAGTTCTCAAGCTT
TCGTGACAGAAAACCCATCTCTTTTACTCCAAAGCCCATATAGCACCCACAA
CTATTAACTGTGACCAAGAAAGAGAAGGCAAGCCCCAATTAACCTTTGTACG
TAAAGCCTAAAGAATGAAAAAATATACCTGAATCCTCAATCATCAAACAGCAT
AGTATATACTAAGTAATTTGTAATAATTAAACTCTAGAAAATTGTGTGGCTTC

GGTAGTAAGAGAGCTTCATGATGTAAAATGGCAAGTGGAGACAGAGACAAA
AGTAGGATGTGGACTGAGAGGGAAGGTTAGCACAGGTGGAACAGTAAGGC
AACCATACTATCAATTGCTGCTGACATAGAATCCAGAGAGACTATTGGCAAA
AGCTCAAATGAGACACAGTAACAGTTTAGATTCAGACAGTGGCTGTGGCATA
AATCAGAAAATTGATAGTCGCATGATCCCTCTTTGCATGGGACTGGCATCTG
TGTGGAGTAATGGTTCCATATGCCTCCTTTCTTCTCCTTATTTTTAAATTTTTT
AAAAATGCATTGCTTCTTGTGGAAGTCAATAAGTGATTCTTCCAATACTTTCT
CATTCCTTCCCCCTCAGTTATGAGACAATTTGCTTATTTCTCATCCATGAATA
CTTGTTGGGTCATTAAAAGTAGATACTGAAATTACTAATGGTACGACTGACA
TATTACCTCATAAATGTTACTAGCTAGATGTTGAAAGTTGACCAACAACTCTC
AAAATATGATTAAGAAAAGGAAACCCACAGAACAGTTTGATTCCAAAATGATT
TTTTTCTTTGCACATGCCTTACTTATTTGGACTTACATTGAAATTTTGCTTTAT
AGGAGAAAGGGTACAACTTGCCCATCATTTCTCTGAACCAGGTATGTTAATA
TTTGACAAAGAATAAAAGTCATTCCATTTTAAACTATCCATTGCTTGTTTCAAA
TGCCTAAGAAAATGTGTCTATCTTAGAAGAGCATATGTTGTTAACTTTATTCA
CACAAAATTGTAAAGGCAAAGAAAATATTCTCTTTTTAAAATTAAAATAGGCA
TTTCTTATTTTTAAAAACATTTTGGGGGCCAGGGGCCGTGGCTCATGCCTAT
AATCCCAGAACTTTGGGAGGCTGAGCCTGGCTAATCGCTTGAGCCCAGGAA
TTTGAGAACAGCCTGGGCAATATGGCGAAATCCATCTCTACAAAAAATACAA
AAATTAGCTGGCATGGGGCACGCACCTGTAGTCTCAGCTACTTGGGAGGCT
GGCTGAGGTGGGAGGATCGGATCCATTGCCTGAGTCTGGGAGTTTAAGGC
TGCAGTGAGCTATGACTGTGCCACTGTACTCTAGCCTTGGTAAGACCCTGT
CTCAAAAACAAATACATAAGTAAATAAAAATAAATAAAAACATTTTGGAAATA
GAAATACATAATTTGGTAATAGTTTTTCTCTTAAGTTAGATGTTTTACCTTTCT
AACCAAGCCTGAGTACTTGAAAAAAGCCTCATAAGAGCTTATAAAACAAATG
AACTTCCCTCATATAAAAAGCAAGGCATTTAAAATCATCTAATTAACTGGTAC
TGTATTTCAAGGGTAAATCTCAGCCTTGATTCATTTTTGGCCCAATGCAACCA
CTTAGGGACCATCTTGACAACCTCTGCTGAAGGGACATCCCTTCCCCTCACT
TGAGTATCACTGTGTGTGCTCATTTGCTATTCTGCATTCCAACCCTCCCTTCA
CACTTGGCTGTGTCCACGGCTCACAGGGTAAAAAGCACATCATAGAACTTC
ATCACTATCGCATACATTCAAGCTAAGTGGTCAAGAAGGCTGGGCAACACC
AGCAAGAGGAAATGCTACTTTTACTTTTTATCAACAATAGGGCTTTTAAATAT
TAATTAGGCAAATAAATGAGCCATTTTACCTTTATGTCTAGCCTTCCATTCTA
TTTACTTCAACTGGAAGCACTACAAATATGCTATAAATATGGAAATATCTCTT
AATTGATTTCAATTGTTTCATTCCCAACATATAAATGACTCAACAAGCATTTTT
AGTGACTACATTGGAGACTATGCATAAGAATACTATGGAAGGAATAAAGCTT
AGAACATAGATGACCTGCATTATAATTATAATTCTACTTTTAACTAGTTGTCT
GACCAAGGCTAAGTTAACCTTATTCAGCTTCTTTTCTTCATTTGTAAACTGTT
TATACCAGTTTCTTTCCAAAATTATGATTCTATGATCTGTTCAATGCTCTTTTA
TACATTAAGACATTATTTTCTCTCATAACTTCCAAACTATGGGAGAATTTGTG
GTTTTTTCCCCATATCTGAGGAGAACGTCCACTGAGTTCTTATCTACAGTTAC
ACTAGTGAAGAACGCTGGGTCTGGAATCAGAAGCTTCAGGTCTTAGTTCTG
TCATCAACTATTTTGCGACCTTGGACAAAAGACTTGATCACTCACAGTCCCA
GTTTCCCACAAGGTTACTGTAAAGCACACAATTTAAAAAAAGACAAAATCTAC
ATAATAGTATATTAATTGTGCTTTCTATTAAAAGGCAAGGTGATGGTATGCTG
ATGTTATCTGTCTTATTTTTCAGTTGCTATATGGTCATTTATTTCAGACTTTCA

TAATTTTGCTGCTCTCTTTATCTCCTGTAGAGATAACACTCATTATTTTTGGG
GTGATGGCTGGTGTTATTGGAACGATCCTCTTAATTTCTTACGGTATTCGCC
GACTGATAAAGGTGAGAATTCAGTTTTTAATTTTGCTGTAAATACCAATGTGA
ACAGCTCTAAGAGGGTTTATTCCTCTGAGTTCAGTTAAACTCAAAAGAGAAA
CAGAACTGCATAAAATTCCATATTTTTCAACTGGACACATAGAAGTCACTGT
GTTTCTCTAGCAGAATTTTTCTTTGCATTTGCCCAATTAAAGGGAACCTCTAA
ATATAAATCTGTCCCCCATTTTCCCAATGAAAGATCTCCCTAAGTTTTTGTCT
AACTTGCTGTCACATATTTTGATGGATATTGAGGAAATATTAAGATTCTACTT
ATAGTATTTACCCTATTAGTGTATAAAATATTTAAAATAATATATTTACATATG
TTTAAAACTTTGAGGGAAGCCAAGGCAGGAGGATTGCTTGAGCTCAGGAGT
TTGAGACCAGCCTGAGCAAAAAGGTGAAACCTAGTCTATACAAAAAATATGA
AAATTAGAAAGGCGTGGTGGTGCACATGTGTAGTATCAGCTACTCAGGGGG
CTGAAGTGGGAGGATTGCTTGAGCCTGGGAAATCAAGGCTGCAGTGAGCT
GTGATCATGCTACTGCACTCCAGCCTGGGCAACAGAGTGAGACCCTGTCTC
AATAATTATATAAATAAATAAATAAAAATAAACAAAATAAAACTTTTGCCTTTC
TTAATTCTCACATATTCTGAAACAGATTTTTCAAATTTCCACCCATGAATTCTT
AACATCAGTGATTTTTTTTGAATCATTAATGCTTTTTTTAATTTTTTTTTTTTTTT
TTGAGACAAGAGTTTCCCTCTGTCACCCAGGCTCGAGTGCAAAGTGGTGCA
ATCTCTGCTCACTGCAGCCTCTGCCTCCCTGGTTTAAGTGATTCTCGTGCTT
CAGCCTCCGCAGTAGTTGGGACTACAGGTGCGGGACACCATGCCTGACTA
ATTTTTGTATTTTTTTAATAGCAGAGATGGGGTTTCGCTGTGTTGGCCAGGC
TGGTTTCAAACTCCTGACCTCAAGTGATCCATCTGCCCTTGGCCTCCAAAGT
GCTGGGATTACAAGCATGAGCCACCACGCCCAGCCCACTAATGCTATTTTT
ACATCCATACAACACAGCTTATCGAAGTGCATAACTTTTGCTATCACTTTCTA
TTCACGATATTTAAGACATAATATGTGTGTGTGTATTTATGATGCTGTCACTG
TCTCTGTAATCCTAGATCAGAAGTACTTAGTCACATGAGATTGGTACAGTTG
TGTTTTCATTCATCCTCTATTCTTAATCTCTCTTTGTGATTTTTGAGACCATAA
CCACTATATAATTCTTTTAAAAAGGCTGAGAGGTGTGACAGCACTGCAATTG
TGGGGCCATCAGAAGATATGATAGTAATATCTACATTAAGTTCCTTTGCCTCT
TTTCTTTTTTAACTACTTCTAACAGTTAACTTCTACCATCATCCAATCCTATAA
TTGATTTTCAGTATTCCATGTAAATATATCTTCCTTAAATAATACTTTTTGTTAA
TCAAAGAAAAGTAACTGAAAATGCCTACTCTTGTGTGAGATATTTTGTAAGG
ACTTTAATATAAGATAGCTTTTTTTGCCTGGAGTATAAAAGAGAAAAGTCATC
TTCTTACATGGGCATATATGGCAAAGTGGGTTGTCTTCTCTCTTCGTCAATG
TTCTAAAACCTGAAAAAGCCAAGGAAATATTTAGTTGGCAAAGTTCAGAGAA
TTTTCTAAGTGTATATGGATGAATTTTGTCCTGGTCAACATGATGCAGAGATC
ACACACTTTATTTTTATTTTTATTTTCACTTTCACTATTTATTACAGCAGGGAA
ATATGTAAGTATCAGTGTTTGAGGTGATATTTCTCCTACTGAAATACCAAATA
CTATAGAGGAACACAAATACAAGTTTAAATCAATGCTTATACCAGTAACTAGT
AACAACAACAATAACAAAATCTCTGCAAAGGGGATTTCAACCAAAAGAAAAA
AAATTTTAGAAAAAAATATTTTTAAGCTGAAGCATTTTACTTTTTACTGTCTTA
AGACTAGAAAATTGTGTTATTAATATTTTATGGTATTTCTTCATAGAAAAGCC
CATCTGATGTAAAACCTCTCCCCTCACCTGACACAGACGTGCCTTTAAGTTC
TGTTGAAATAGAAAATCCAGGTTGGTGTTAATATTTGCAGTTCCTTTTGCCTT
TTAGGAAAAAAAAATCAAACCAGTGAGTTACTTCTTTCTGATTTGAGGGAGG
AGGGAACCAGTTATGATTCATTTCTATTCTATCTCATTAATTCTACTTCTTTGA

CTTTTTAGAAATGTCTGCAGCATAGTGAGATTCTCCTTTGGACACAAAGTGTT
TTGTTTTGTTTTGTTTTTTTAACAAAAAAAAAAAAAACTCAATCAAATAGTAAAA
GCAAAAGAGAAAACCAAGTGTACTTCGTATTTCCCAAACTGCAAAGTTATGT
GTATAGGAGACTCTATGGTCAGTATGGTGTAGCATAGTGAATTAGCCCCAG
ATCTGAAATCAGACTTGGATTTGAATCCATGCTCCAACACCTATTAGCTGTG
TAACCCTGAGCAAGCTACTAAACCTCTTTTAATATGGGGATAATGATAGTAT
CAACCTCACAAAGTTTAATGAGAATTAAATGAGCTACAACCGGTAAAGCATT
TAAAACCATTTGTGGCCATCATAAGTCCTCATGCCTGTTAGCTGTTATCAATA
TAGCACTGACATCAATGCTATATCAATATAGCATGTTATCAATATAGTGTCAT
TCCCAAATGACCTCCTGTGCACACTGGCAAGCCATCTGGCACATGCTTTCAT
CTCCACTCCCAGGTGCTAAGCAGATACAAAACATGTGAAAGGCCATGGATA
TATTTTGTTTATCCAGAACAGTATTAAACCACATAGTGCTTTTTGAAAAGAAT
ATTTATTGTCAACCTTTAAAAGTCGGAAATTGTTACATTTTAAAAATCAAGTAT
TGCTATTCCTCTGGGGAAAAATGTAAACTCCCAAAATGCTGAGAGCCTTCAT
ACCAGCATGAGACCAATTCCTAAGAGCTGAGTAGTGGCTGCTACCTGTACT
GTCTGTCTAAATCCCTAGCCAATTGCATTTGTTTTATTCACCGTGGCCCCTG
GTATGAACTCACTAAGAAAGCATATAGTTTCTATTAAACTTTGCCTGAAGCAT
AAACCCAAATGACATCTATTTTGGGAGATAGTTACTAAGAACAAGTCTCTGG
AATGAGCTTTATTTCTCAAGCAAAAGAGATTTCATTCTGCCTTCTACAAAATC
AACTGATTTTACTCCCATAATTTTCAGAAATCATGACAGATCAGAGGTCCTGT
ATGCTTCTGGATTTCGATTTTAACCCTGGGCCAGTCTAGGTTTTCTAGACTTT
AGAGTCACAGAACACAGAGTTTTCAAGATCCATCACAGCTACACAGGTTATA
TGCAGGATTTGCCACATCACATTATCATGTGAATTCTTAAAGCTTAAGAGTAA
TTGTTACATAAGTTTATAATCCTAAGACATTCCTGCTATGTGGAAATGAATGG
CATAGATATGATTCTCAGCTAAAAGGATTAATAAAATCCAATCTGCAGATACT
TGAAACAACGGAAGTTTTTGAGTCATATGCCAGATTCACTTCATTTACTAAG
GTTATCTTGTTATTGGACTGGCAGCTGGAACAAGTATCTGTAAAATATTCATT
TTATCTGCATTCTGCCTTGTTCCACAAAAAAGTCTTGATGTAGTTTTTCAAGT
GGAGCAATTACAACCTAAAGCCTATTTTTCGAACTGAAATTTATATACATTTT
TAGCTACTTATTTATTCTAGAGACAAATTTATTGTTTAGAGTTTCCCCTGCCA
TTTTTTTCATACAATTTTAAGCATCTCAAATGTTTGGCACAATTTAATACGCCA
CAGTGCATCAAGATGTCCTTGTAGTTTAATTCAGTTAAGTGCAACAAACATTT
GCTAAATGCATACAGTGGGGTAGGCACCACACTCACATTAGATATACCAATA
TGAGTCTTCGTCCTTTAGAAGCTGAGAGACTAATGGAAAAAACAGAATGTCA
TTGCAGTGAACAAGTTCTACAGTAGTGGAGGCAATAGCTCCACTTGTCCCA
GAGACTGAGACAGGTATCAAAGGCTTCTGAAGATGAAATCACCTGGGATTA
GCCTTAAAAGACAGATAGATATTAGCTAGGGCAGGGTAGTTTTAGCAGAAG
GGCAGCCTGAGTGAGTAAAAGCATGGAAGACAGAATATGTTTACTTAAAGAA
TTGTATGCATTTCCACATTAGCAGGATTGCTGCTTTGGTTCTCTGTTCACATC
TCAAATATGTGTAATGGCAGTGGAAAGTCAGAAGAACCAAACTTTAGGCTCA
CTTTATTTCCCCACATTTGTGCAAGTGAAGTTATTAAATGTCTTAGTATGTTA
GTGAGACAAGTTATGAATTCTGACTGCACCTCACAGAAAACATAGGAAAACA
CATTATTAAAGATTATTTAAAATGCTTTATTTCTACTTTTATAGAATATGGCTC
TAAATTAGTTTATAAGCCAAAGGCATAAGAGGTTAAAATGACAGTACCATCT
CAACAAGAACTAATGATGTAAAGGAGTAATTAGAGTATAAATTGTTTTAACCT
TCTAAAAGTGCACATGATCTGTGATTGGTGAAAAATGAGAATAAGCGAATCT

GAGTCAGCTGGCCACTGTGGCATGCATATGTGACCCACTAGCCTATTTCCC
ACAGGAGAATGTTTGAGATGCACAGTTCCTGTGGTGCCCAAATAGAAGAAG
GCTGGAAAAGCTCTGCTTCTGGAAGAGCAAGGGCTCCCCTCTCCCTTTCAT
GCAGTTTCTAGGAGCAACATAAATTCAACCTTCCAACCAGGAAAAGTGGAG
CATCGGGTTTACTGGAGAAAACTAGCCCAGTGCCCTTCTTTTACACCCTAGA
ACCAGAGAGGAACTTGGCCATAAGCTTTTGTGCAGACTTCTCCTTGGGGGA
AAAAAAAAGTCATTATTTAAAAAGACATGACAGACTTAGACACATGCCTTAAA
TTTTAACATGCATATGTGATTCAACTTATCATTTACTGGCTTCACATTATATTT
TGCCTCTATACAAGTTTGGCTGTTTGTTTCTTATCTCTGTAGAAACTAGGAGC
AGAGCAATTATATTTATTCTTTACCTAAGGCTTTTAGAATAGATATTCTAAGAA
ATTCTGTATTTTTCTTTACACAAAACTTGACAATAGAGCTAATATGTAAGGAG
AGTCCTTTCGTTTCCTACTAATTACATTCAAGAACAACTCTGCAAGAATGTAG
AATCCTAAAATGTATACTGTGCATTAATTTCCTGTTGTGTTTAAACATAACTAT
GTCTCATATTTCGGTCTTGTATTTTTTTTACTATAATCCTTCTAGAGACAAGTG
ATCAATGAGAATCTGTTCACCAAACCAAATGTGGAAAGAACACAAAGAAGAC
ATAAGACTTCAGTCAAGTGAAAAATTAACATGTGGACTGGACACTCCAATAA
ATTATATACCTGCCTAAGTTGTACAATTTCAGAATGCAATTTTCATTATAATGA
GTTCCAGTGACTCAATGATGGGGAAAAAAATCTCTGCTCATTAATATTTCAA
GATAAAGAACAAATGTTTCCTTGAATGCTTGCTTTTGTGTGTTAGCATAATTT
TTAGAATTGTTTGAGAATTCTGATCCAAAACTTTAGTTGAATTCATCTACGTTT
GTTTAATATTAACTTAACCTATTCTATTGTATTATAATGATGATTCTGTCAAAT
GAAAGGCTTGAAATACCTAGATGAAGTTTAGATTTTCTTCCTATTGTAAACTT
TTGAGTCTGGTTTCATTGTTTTAAATAAATTAAGGGGACACTAAAGTCCTATC
ATTCATTTCCTTCATTGCTGAACAGGCAAGATATAATATTACATGAATGATTA
CTATATTTTGTTCACACTAATAAAGCTTATGCTCAGAAATGCCATACACACAC
ACACACACACACACAAACACACACATTTATCATTTAATGCATAAATCAACACA
AAAGGTTTTCCCATTAATATGAAATATTACATATATATAAGTGCCATATTTAAA
ATAATTTGTCTAACAGTAGAACTGTGTCGGAGCACTCACTGAAGCTTGCATT
CCACTGAAAGAGTTATTTGTGTAAGTAGAGTATCCGGAGAAGGAAAAGAACT
TACGACCTTTCTTTATAACAGAAACTCAACTCTAAATTCAACAAGATGTGCAA
ACCGGACATGCAGGTGAATATTTTAATAGGTTACTATAAGGTTCTCAATTAAA
TTCTTTAATCTGTCCAGTCCCAGTTTCTCTTATTAATAAAACTTTGGAAATTGC
TTTAAACCATTTAAAGGAAATTTCTAGATATAGAAACTAAGGACTGTGACTAT
ACAGCTGTCACTCATTTGTAGTAAAACTTAAAAAGCAAAAACAAAAAACAAAA
AAGACCTTCCTGTGATACTTTATTTCCGAACTAATAAAAATCTATATGACTTTT
TATTATTGTGTGATAACCAAGTAAATGTTTTCTATTTTGCATATTTTCAGGCAT
GGTAACAGAAATTTACCTTTTAATAAATTAAAAAATCTAAATTTTAACCTACTT
GTATGTTCGGAGAGTGTTTTTGTACTATATTGACTACTTAAAATAGAGAATGA
GACTAAGAAGGGAACATTTCTGTTGATACATGTTTTTTAAAAGTAATTTTAAG
AGCATTATTAGGTTAATTAATCCAATTAATGACCCAAATGCCAAGGTAATTTT
AAATTTACATTTTTAATAAAAGCAACATGTTGAAACAAGAGAGGGTGAGATTA
ACCTTTTTGCTAAAGTAATTTACAAGTCAAAGACAGGAAGAGATCAGAGTGA
ATGTGCCTTCTTAACCAGAGCTACAGAATTTAGTGAATAATTAAAGTACAAAC
TGCTTTGACCTCCTTGAACTTTTCCAAGCAATTTCTCTGTACTTCTATATATG
AATGTCTTAGCCAATTTTCTGCTACTATAACAGAATACGACAGACTGGGTAA
TTTAAAAAGAAAAGAAATTTATTTTCTTCCTAGTTCTGGAGGCTGGGAAGGC

GAAGGGCATGGCACTGACATCTGCCTTGTAACTGATGAGAACCTTCTTACT
GCATGATAACAAAGCAGCAAGGCAAGCAAAAGCGTAAGATGAAGAGAGAGG
AAATGAAGCCAAACACATCCTTTCATCAGAAGCCCATTCCCTCTATAAGGCG
TTATTACATTTATGAGAATGGAGTCCTCATGACCTAATCGTGACCTTAAAGG
CCCCTCCCAACACTGTTACAATGGCAATTAAATTTCAACAAAGGTTCCAGAG
GTGACATTCGAATCAGCAATGAAATTTTCATAGTTAAATTTGGTATTCGTGGG
GGAAGAAATGACCATTTCCCTTGTATTTTTATAATTAAATCAGCAAAATATTG
TAATAAAGAAATCTTTCCTGTGAAGATACCATGACCCCAn

Selected Module Sets:
Selected Module Count: 3
Ignore Asp Overlap: True

The results below are zinc finger arrays that can be constructed using CoDA. Note that other methods (including modular assembly and OPEN) can also potentially be used to target the input sequence of interest."

Sort By: Position

ZFA-unknown-1
0 nGCAGGAAGGt 10
0 nCGTCCTTCCa 10

FINGER	HELIX	TRIPLET	REFERENCE NUMBER	SOURCE
F1	RPHHLDA	AGG	-	CoDA
F2	QSAHLKR	GGA	-	CoDA
F3	QDVSLVR	GCA	-	CoDA

ZF DNA Sequence — Bos taurus (cow) Build 3.1

Blast GCAGGAAGG

ZFA-unknown-2
4 gGAAGGTGGGc 14
4 cCTTCCACCCg 14

FINGER	HELIX	TRIPLET	REFERENCE NUMBER	SOURCE
F1	KGDHLRR	GGG	-	CoDA
F2	EAHHLSR	GGT	-	CoDA
F3	QDGNLTR	GAA	-	CoDA

ZF DNA Sequence — Bos taurus (cow) Build 3.1

Blast GAAGGTGGG

ZFA-unknown-3
17 gGAAGATAACa 27

17 cCTTCTATTGt 27

FINGER	HELIX	TRIPLET	REFERENCE NUMBER	SOURCE
F1	GHTALRN	AAC	-	CoDA
F2	VRHNLTR	GAT	-	CoDA
F3	QRNNLGR	GAA	-	CoDA

ZF DNA Sequence Bos taurus (cow) Build 3.1

Blast GAAGATAAC

ZFA-unknown-4
58 tGCAGTTGTCt 68
58 aCGTCAACAGa 68

FINGER	HELIX	TRIPLET	REFERENCE NUMBER	SOURCE
F1	TKKILTV	GTC	-	CoDA
F2	HKSSLTR	GTT	-	CoDA
F3	QGNTLTR	GCA	-	CoDA

ZF DNA Sequence Bos taurus (cow) Build 3.1

Blast GCAGTTGTC

ZFA-unknown-5
276 gGAAGAGGAAa 266
276 cCTTCTCCTTt 266

FINGER	HELIX	TRIPLET	REFERENCE NUMBER	SOURCE
F1	QASNLLR	GAA	-	CoDA
F2	RQDNLGR	GAG	-	CoDA
F3	QRNNLGR	GAA	-	CoDA

ZF DNA Sequence Bos taurus (cow) Build 3.1

Blast GAAGAGGAA

ZFA-unknown-6
282 gGTGGATGGAt 292
282 cCACCTACCTa 292

FINGER	HELIX	TRIPLET	REFERENCE NUMBER	SOURCE
F1	DKTKLRV	GGA	-	CoDA
F2	VRHNLTR	GAT	-	CoDA
F3	RRAALGP	GTG	-	CoDA

ZF DNA Sequence Bos taurus (cow) Build 3.1

Blast GTGGATGGA

ZFA-unknown-7
312 gGTAGCTGAGt 302
312 cCATCGACTCa 302

FINGER	HELIX	TRIPLET	REFERENCE NUMBER	SOURCE
F1	KHSNLTR	GAG	-	CoDA
F2	QRSDLTR	GCT	-	CoDA
F3	QSGTLTR	GTA	-	CoDA

ZF DNA Sequence Bos taurus (cow) Build 3.1

Blast GTAGCTGAG

ZFA-unknown-8
314 aTGTGGGGGAa 324
314 tACACCCCCTt 324

FINGER	HELIX	TRIPLET	REFERENCE NUMBER	SOURCE
F1	TSAHLAR	GGA	-	CoDA
F2	RREHLVR	GGG	-	CoDA
F3	QRHGLSS	TGT	-	CoDA

ZF DNA Sequence Bos taurus (cow) Build 3.1

Blast TGTGGGGGA

ZFA-unknown-9
315 tGTGGGGGAAa 325
315 aCACCCCCTTt 325

FINGER	HELIX	TRIPLET	REFERENCE NUMBER	SOURCE
F1	QLSNLTR	GAA	-	CoDA
F2	RREHLVR	GGG	-	CoDA
F3	RPDALPR	GTG	-	CoDA

ZF DNA Sequence Bos taurus (cow) Build 3.1

Blast GTGGGGGAA

ZFA-unknown-10
392 cTAAGTCTAGc 402
392 gATTCAGATCg 402

FINGER	HELIX	TRIPLET	REFERENCE NUMBER	SOURCE

F1	RGTNLRT	TAG	-	CoDA
F2	DHSSLKR	GTC	-	CoDA
F3	QQGNLGL	TAA	-	CoDA

ZF DNA Sequence Bos taurus (cow) Build 3.1

Blast TAAGTCTAG

ZFA-unknown-11
477 aTAAGGAGATt 487
477 tATTCCTCTAa 487

FINGER	HELIX	TRIPLET	REFERENCE NUMBER	SOURCE
F1	TRQRLRI	GAT	-	CoDA
F2	QSAHLKR	GGA	-	CoDA
F3	QRGNLNM	TAA	-	CoDA

ZF DNA Sequence Bos taurus (cow) Build 3.1

Blast TAAGGAGAT

ZFA-unknown-12
513 cGAAGATGTTg 523
513 gCTTCTACAAc 523

FINGER	HELIX	TRIPLET	REFERENCE NUMBER	SOURCE
F1	TNQALGV	GTT	-	CoDA
F2	VRHNLTR	GAT	-	CoDA
F3	QRNNLGR	GAA	-	CoDA

ZF DNA Sequence Bos taurus (cow) Build 3.1

Blast GAAGATGTT

ZFA-unknown-13
516 aGATGTTGGTa 526
516 tCTACAACCAt 526

FINGER	HELIX	TRIPLET	REFERENCE NUMBER	SOURCE
F1	RRQKLTI	GGT	-	CoDA
F2	HKSSLTR	GTT	-	CoDA
F3	ISHNLAR	GAT	-	CoDA

ZF DNA Sequence Bos taurus (cow) Build 3.1

Blast GATGTTGGT

ZFA-unknown-14
610 gTGGGGAGTTg 620
610 cACCCCTCAAc 620

FINGER	HELIX	TRIPLET	REFERENCE NUMBER	SOURCE
F1	TTALLKR	GTT	-	CoDA
F2	QSAHLKR	GGA	-	CoDA
F3	RSDHLSL	TGG	-	CoDA

ZF DNA Sequence Bos taurus (cow) Build 3.1

Blast TGGGGAGTT

ZFA-unknown-15
613 gGGAGTTGTTc 623
613 cCCTCAACAAg 623

FINGER	HELIX	TRIPLET	REFERENCE NUMBER	SOURCE
F1	TKPVLKI	GTT	-	CoDA
F2	HKSSLTR	GTT	-	CoDA
F3	QTTHLSR	GGA	-	CoDA

ZF DNA Sequence Bos taurus (cow) Build 3.1

Blast GGAGTTGTT

ZFA-unknown-16
629 aGAAGCGAACa 619
629 tCTTCGCTTGt 619

FINGER	HELIX	TRIPLET	REFERENCE NUMBER	SOURCE
F1	GHTALAL	AAC	-	CoDA
F2	RTDTLAR	GCG	-	CoDA
F3	QTNNLGR	GAA	-	CoDA

ZF DNA Sequence Bos taurus (cow) Build 3.1

Blast GAAGCGAAC

ZFA-unknown-17
656 aTAGGAAGAAa 666
656 tATCCTTCTTt 666

FINGER	HELIX	TRIPLET	REFERENCE NUMBER	SOURCE
F1	QASNLTR	GAA	-	CoDA
F2	QQTNLTR	GAA	-	CoDA
F3	RRDHLSL	TAG	-	CoDA

ZF DNA Sequence
Bos taurus (cow) Build 3.1
Blast TAGGAAGAA

ZFA-unknown-18
879 aGAGGATGTTt 869
879 tCTCCTACAAa 869

FINGER	HELIX	TRIPLET	REFERENCE NUMBER	SOURCE
F1	TNQALGV	GTT	-	CoDA
F2	VRHNLTR	GAT	-	CoDA
F3	REDNLPR	GAG	-	CoDA

ZF DNA Sequence
Bos taurus (cow) Build 3.1
Blast GAGGATGTT

ZFA-unknown-19
923 gTGTGCTGGGc 913
923 cACACGACCCg 913

FINGER	HELIX	TRIPLET	REFERENCE NUMBER	SOURCE
F1	KKDHLHR	GGG	-	CoDA
F2	QRSDLTR	GCT	-	CoDA
F3	QPHGLRH	TGT	-	CoDA

ZF DNA Sequence
Bos taurus (cow) Build 3.1
Blast TGTGCTGGG

ZFA-unknown-20
932 gGCAGTTGCTt 942
932 cCGTCAACGAa 942

FINGER	HELIX	TRIPLET	REFERENCE NUMBER	SOURCE
F1	TKQILGR	GCT	-	CoDA
F2	HKSSLTR	GTT	-	CoDA
F3	QGNTLTR	GCA	-	CoDA

ZF DNA Sequence
Bos taurus (cow) Build 3.1
Blast GCAGTTGCT

ZFA-unknown-21
949 tTCTGAGTCTa 959
949 aAGACTCAGAt 959

FINGER	HELIX	TRIPLET	REFERENCE NUMBER	SOURCE
F1	SKPNLKM	TCT	-	CoDA
F2	RQDNLGR	GAG	-	CoDA
F3	QRNTLKG	TCT	-	CoDA

ZF DNA Sequence

Bos taurus (cow) Build 3.1

Blast TCTGAGTCT

ZFA-unknown-22
1026 gGGAGGAGAGa 1036
1026 cCCTCCTCTCt 1036

FINGER	HELIX	TRIPLET	REFERENCE NUMBER	SOURCE
F1	KHSNLTR	GAG	-	CoDA
F2	QSAHLKR	GGA	-	CoDA
F3	QMSHLKR	GGA	-	CoDA

ZF DNA Sequence

Bos taurus (cow) Build 3.1

Blast GGAGGAGAG

ZFA-unknown-23
1144 gGCTGGGGAAc 1154
1144 cCGACCCCTTg 1154

FINGER	HELIX	TRIPLET	REFERENCE NUMBER	SOURCE
F1	QLSNLTR	GAA	-	CoDA
F2	RREHLVR	GGG	-	CoDA
F3	VSNSLAR	GCT	-	CoDA

ZF DNA Sequence

Bos taurus (cow) Build 3.1

Blast GCTGGGGAA

ZFA-unknown-24
1197 aGACGAGGACa 1187
1197 tCTGCTCCTGt 1187

FINGER	HELIX	TRIPLET	REFERENCE NUMBER	SOURCE
F1	DEANLRR	GAC	-	CoDA
F2	RQDNLGR	GAG	-	CoDA
F3	DPSNLRR	GAC	-	CoDA

ZF DNA Sequence

Bos taurus (cow) Build 3.1

Blast GACGAGGAC

ZFA-unknown-25
1322 cTGTGATTAGa 1332
1322 gACACTAATCt 1332

FINGER	HELIX	TRIPLET	REFERENCE NUMBER	SOURCE
F1	RSHNLRL	TAG	-	CoDA
F2	VRHNLTR	GAT	-	CoDA
F3	QPHGLAH	TGT	-	CoDA

ZF DNA Sequence
Bos taurus (cow) Build 3.1
Blast TGTGATTAG

ZFA-unknown-26
1376 aTAAGCAGTAg 1386
1376 tATTCGTCATc 1386

FINGER	HELIX	TRIPLET	REFERENCE NUMBER	SOURCE
F1	QKQALTR	GTA	-	CoDA
F2	QSTTLKR	GCA	-	CoDA
F3	QGGNLAL	TAA	-	CoDA

ZF DNA Sequence
Bos taurus (cow) Build 3.1
Blast TAAGCAGTA

ZFA-unknown-27
1379 aGCAGTAGCTa 1389
1379 tCGTCATCGAt 1389

FINGER	HELIX	TRIPLET	REFERENCE NUMBER	SOURCE
F1	NGQGLRR	GCT	-	CoDA
F2	QRSSLVR	GTA	-	CoDA
F3	QDNTLRR	GCA	-	CoDA

ZF DNA Sequence
Bos taurus (cow) Build 3.1
Blast GCAGTAGCT

ZFA-unknown-28
1420 gTAGGTTTGTt 1410
1420 cATCCAAACAa 1410

FINGER	HELIX	TRIPLET	REFERENCE NUMBER	SOURCE
F1	RRQHLQY	TGT	-	CoDA
F2	HKSSLTR	GTT	-	CoDA

F3	RPESLAP	TAG	-	CoDA

ZF DNA Sequence

Bos taurus (cow) Build 3.1

Blast TAGGTTTGT

ZFA-unknown-29
1507 tTAAGCAGAAa 1517
1507 aATTCGTCTTt 1517

FINGER	HELIX	TRIPLET	REFERENCE NUMBER	SOURCE
F1	QGSNLAR	GAA	-	CoDA
F2	QSTTLKR	GCA	-	CoDA
F3	QGGNLAL	TAA	-	CoDA

ZF DNA Sequence

Bos taurus (cow) Build 3.1

Blast TAAGCAGAA

ZFA-unknown-30
1561 gGATGTAGTGt 1571
1561 cCTACATCACa 1571

FINGER	HELIX	TRIPLET	REFERENCE NUMBER	SOURCE
F1	RKHILIH	GTG	-	CoDA
F2	QRSSLVR	GTA	-	CoDA
F3	VGHNLSR	GAT	-	CoDA

ZF DNA Sequence

Bos taurus (cow) Build 3.1

Blast GATGTAGTG

ZFA-unknown-31
1633 tTGTGTTGCTc 1643
1633 aACACAACGAg 1643

FINGER	HELIX	TRIPLET	REFERENCE NUMBER	SOURCE
F1	TKQILGR	GCT	-	CoDA
F2	HKSSLTR	GTT	-	CoDA
F3	QPHGLAH	TGT	-	CoDA

ZF DNA Sequence

Bos taurus (cow) Build 3.1

Blast TGTGTTGCT

ZFA-unknown-32
1646 tGGGGCAAACc 1656
1646 aCCCCGTTTGg 1656

FINGER	HELIX	TRIPLET	REFERENCE NUMBER	SOURCE
F1	GHTALRN	AAC	-	CoDA
F2	QSTTLKR	GCA	-	CoDA
F3	RTEHLAR	GGG	-	CoDA

ZF DNA Sequence

Bos taurus (cow) Build 3.1

Blast GGGGCAAAC

ZFA-unknown-33

1700 cTGGGGGTGTg 1710

1700 gACCCCCACAc 1710

FINGER	HELIX	TRIPLET	REFERENCE NUMBER	SOURCE
F1	RKQHLTL	TGT	-	CoDA
F2	RREHLVR	GGG	-	CoDA
F3	RMDHLAG	TGG	-	CoDA

ZF DNA Sequence

Bos taurus (cow) Build 3.1

Blast TGGGGGTGT

ZFA-unknown-34

1701 tGGGGGTGTGc 1711

1701 aCCCCCACACg 1711

FINGER	HELIX	TRIPLET	REFERENCE NUMBER	SOURCE
F1	RRFILSR	GTG	-	CoDA
F2	EAHHLSR	GGT	-	CoDA
F3	RTEHLAR	GGG	-	CoDA

ZF DNA Sequence

Bos taurus (cow) Build 3.1

Blast GGGGGTGTG

ZFA-unknown-35

1719 tTGGGAAAACt 1729

1719 aACCCTTTTGa 1729

FINGER	HELIX	TRIPLET	REFERENCE NUMBER	SOURCE
F1	GASALRQ	AAC	-	CoDA
F2	QQTNLTR	GAA	-	CoDA
F3	RSDHLSL	TGG	-	CoDA

ZF DNA Sequence

Bos taurus (cow) Build 3.1

Blast TGGGAAAAC

ZFA-unknown-36
1807 aGTTGTAGAAa 1797
1807 tCAACATCTTt 1797

FINGER	HELIX	TRIPLET	REFERENCE NUMBER	SOURCE
F1	HRPNLTR	GAA	-	CoDA
F2	QRSSLVR	GTA	-	CoDA
F3	HHNSLTR	GTT	-	CoDA

ZF DNA Sequence   Bos taurus (cow) Build 3.1

Blast GTTGTAGAA

ZFA-unknown-37
1810 tGTAGTTGTAg 1800
1810 aCATCAACATc 1800

FINGER	HELIX	TRIPLET	REFERENCE NUMBER	SOURCE
F1	QQQALKR	GTA	-	CoDA
F2	HKSSLTR	GTT	-	CoDA
F3	QSTSLQR	GTA	-	CoDA

ZF DNA Sequence   Bos taurus (cow) Build 3.1

Blast GTAGTTGTA

ZFA-unknown-38
1813 tTGTGTAGTTg 1803
1813 aACACATCAAc 1803

FINGER	HELIX	TRIPLET	REFERENCE NUMBER	SOURCE
F1	TSTRLDI	GTT	-	CoDA
F2	QRSSLVR	GTA	-	CoDA
F3	QPHGLAH	TGT	-	CoDA

ZF DNA Sequence   Bos taurus (cow) Build 3.1

Blast TGTGTAGTT

ZFA-unknown-39
1819 aTCTGCTTGTg 1809
1819 tAGACGAACAc 1809

FINGER	HELIX	TRIPLET	REFERENCE NUMBER	SOURCE

F1	KRQHLEY	TGT	-	CoDA
F2	QRSDLTR	GCT	-	CoDA
F3	QRNTLKG	TCT	-	CoDA

ZF DNA Sequence Bos taurus (cow) Build 3.1

Blast TCTGCTTGT

ZFA-unknown-40
1910 aGATGATAACa 1900
1910 tCTACTATTGt 1900

FINGER	HELIX	TRIPLET	REFERENCE NUMBER	SOURCE
F1	GHTALRN	AAC	-	CoDA
F2	VRHNLTR	GAT	-	CoDA
F3	ISHNLAR	GAT	-	CoDA

ZF DNA Sequence Bos taurus (cow) Build 3.1

Blast GATGATAAC

ZFA-unknown-41
1945 tGATGTGGCTa 1955
1945 aCTACACCGAt 1955

FINGER	HELIX	TRIPLET	REFERENCE NUMBER	SOURCE
F1	TKPILVR	GCT	-	CoDA
F2	RREVLEN	GTG	-	CoDA
F3	ISHNLAR	GAT	-	CoDA

ZF DNA Sequence Bos taurus (cow) Build 3.1

Blast GATGTGGCT

ZFA-unknown-42
2008 aGCTGAAGAGt 2018
2008 tCGACTTCTCa 2018

FINGER	HELIX	TRIPLET	REFERENCE NUMBER	SOURCE
F1	KHSNLTR	GAG	-	CoDA
F2	QQTNLTR	GAA	-	CoDA
F3	VGNSLTR	GCT	-	CoDA

ZF DNA Sequence Bos taurus (cow) Build 3.1

Blast GCTGAAGAG

ZFA-unknown-43
2024 tGGTGAAGTCt 2034
2024 aCCACTTCAGa 2034

FINGER	HELIX	TRIPLET	REFERENCE NUMBER	SOURCE
F1	TSTLLNR	GTC	-	CoDA
F2	QQTNLTR	GAA	-	CoDA
F3	IRHHLKR	GGT	-	CoDA

ZF DNA Sequence Bos taurus (cow) Build 3.1

Blast GGTGAAGTC

ZFA-unknown-44
2068 aGATGATTGCc 2078
2068 tCTACTAACGg 2078

FINGER	HELIX	TRIPLET	REFERENCE NUMBER	SOURCE
F1	RKRNLIM	TGC	-	CoDA
F2	VRHNLTR	GAT	-	CoDA
F3	ISHNLAR	GAT	-	CoDA

ZF DNA Sequence Bos taurus (cow) Build 3.1

Blast GATGATTGC

ZFA-unknown-45
2133 gTAAGTTTAGa 2123
2133 cATTCAAATCt 2123

FINGER	HELIX	TRIPLET	REFERENCE NUMBER	SOURCE
F1	RSHNLRL	TAG	-	CoDA
F2	HKSSLTR	GTT	-	CoDA
F3	QQGNLQL	TAA	-	CoDA

ZF DNA Sequence Bos taurus (cow) Build 3.1

Blast TAAGTTTAG

ZFA-unknown-46
2148 gGAAGGAGATc 2138
2148 cCTTCCTCTAg 2138

FINGER	HELIX	TRIPLET	REFERENCE NUMBER	SOURCE
F1	TRQRLRI	GAT	-	CoDA
F2	QSAHLKR	GGA	-	CoDA
F3	LGENLRR	GAA	-	CoDA

ZF DNA Sequence — Bos taurus (cow) Build 3.1

Blast GAAGGAGAT

ZFA-unknown-47
2265 tGCTGTGGGCt 2255
2265 aCGACACCCGa 2255

FINGER	HELIX	TRIPLET	REFERENCE NUMBER	SOURCE
F1	SPSKLVR	GGC	-	CoDA
F2	RREVLEN	GTG	-	CoDA
F3	VGASLKR	GCT	-	CoDA

ZF DNA Sequence — Bos taurus (cow) Build 3.1

Blast GCTGTGGGC

ZFA-unknown-48
2298 gGAGGGTAGGc 2308
2298 cCTCCCATCCg 2308

FINGER	HELIX	TRIPLET	REFERENCE NUMBER	SOURCE
F1	RRAHLLS	AGG	-	CoDA
F2	EAHHLSR	GGT	-	CoDA
F3	RGDNLKR	GAG	-	CoDA

ZF DNA Sequence — Bos taurus (cow) Build 3.1

Blast GAGGGTAGG

ZFA-unknown-49
2302 gGTAGGCGCAg 2312
2302 cCATCCGCGTc 2312

FINGER	HELIX	TRIPLET	REFERENCE NUMBER	SOURCE
F1	RRQELHR	GCA	-	CoDA
F2	LKEHLTR	GGC	-	CoDA
F3	QTQSLQR	GTA	-	CoDA

ZF DNA Sequence — Bos taurus (cow) Build 3.1

Blast GTAGGCGCA

ZFA-unknown-50
2305 aGGCGCAGTGg 2315
2305 tCCGCGTCACc 2315

FINGER	HELIX	TRIPLET	REFERENCE NUMBER	SOURCE
F1	RSHILTN	GTG	-	CoDA
F2	QSTTLKR	GCA	-	CoDA
F3	DGGHLTR	GGC	-	CoDA

ZF DNA Sequence Bos taurus (cow) Build 3.1

Blast GGCGCAGTG

ZFA-unknown-51
2311 aGTGGATGATg 2321
2311 tCACCTACTAc 2321

FINGER	HELIX	TRIPLET	REFERENCE NUMBER	SOURCE
F1	TKQRLVV	GAT	-	CoDA
F2	VRHNLTR	GAT	-	CoDA
F3	RRAALGP	GTG	-	CoDA

ZF DNA Sequence Bos taurus (cow) Build 3.1

Blast GTGGATGAT

ZFA-unknown-52
2314 gGATGATGGGa 2324
2314 cCTACTACCCt 2324

FINGER	HELIX	TRIPLET	REFERENCE NUMBER	SOURCE
F1	KKDHLHR	GGG	-	CoDA
F2	VRHNLTR	GAT	-	CoDA
F3	ISHNLAR	GAT	-	CoDA

ZF DNA Sequence Bos taurus (cow) Build 3.1

Blast GATGATGGG

ZFA-unknown-53
2319 aTGGGAGGGAa 2329
2319 tACCCTCCCTt 2329

FINGER	HELIX	TRIPLET	REFERENCE NUMBER	SOURCE
F1	THAHLTR	GGA	-	CoDA
F2	RQDNLGR	GAG	-	CoDA
F3	RMDHLAG	TGG	-	CoDA

ZF DNA Sequence Bos taurus (cow) Build 3.1

Blast TGGGAGGGA

ZFA-unknown-54
2321 gGGAGGGAACg 2331
2321 cCCTCCCTTGc 2331

FINGER	HELIX	TRIPLET	REFERENCE NUMBER	SOURCE
F1	HRTNLIA	AAC	-	CoDA
F2	RREHLVR	GGG	-	CoDA
F3	QTTHLRR	GGA	-	CoDA

ZF DNA Sequence
Bos taurus (cow) Build 3.1
Blast GGAGGGAAC

ZFA-unknown-55
2322 gGAGGGAACGc 2332
2322 cCTCCCTTGCg 2332

FINGER	HELIX	TRIPLET	REFERENCE NUMBER	SOURCE
F1	KRIDLQR	ACG	-	CoDA
F2	QSAHLKR	GGA	-	CoDA
F3	VHWNLMR	GAG	-	CoDA

ZF DNA Sequence
Bos taurus (cow) Build 3.1
Blast GAGGGAACG

ZFA-unknown-56
2355 aGTTGTTGTTc 2345
2355 tCAACAACAAg 2345

FINGER	HELIX	TRIPLET	REFERENCE NUMBER	SOURCE
F1	TKPVLKI	GTT	-	CoDA
F2	HKSSLTR	GTT	-	CoDA
F3	IRTSLKR	GTT	-	CoDA

ZF DNA Sequence
Bos taurus (cow) Build 3.1
Blast GTTGTTGTT

ZFA-unknown-57
2412 gTAAGATGATa 2422
2412 cATTCTACTAt 2422

FINGER	HELIX	TRIPLET	REFERENCE NUMBER	SOURCE
F1	TKQRLVV	GAT	-	CoDA
F2	VRHNLTR	GAT	-	CoDA

F3	QQGNLQL	TAA	-	CoDA

Bos taurus (cow) Build 3.1

ZF DNA Sequence

Blast TAAGATGAT

ZFA-unknown-58
2447 tTAAGCAGCTt 2437
2447 aATTCGTCGAa 2437

FINGER	HELIX	TRIPLET	REFERENCE NUMBER	SOURCE
F1	TKQILGR	GCT	-	CoDA
F2	QSTTLKR	GCA	-	CoDA
F3	QGGNLAL	TAA	-	CoDA

Bos taurus (cow) Build 3.1

ZF DNA Sequence

Blast TAAGCAGCT

ZFA-unknown-59
2454 gGTAGCAGGGa 2464
2454 cCATCGTCCCt 2464

FINGER	HELIX	TRIPLET	REFERENCE NUMBER	SOURCE
F1	RRAHLQN	GGG	-	CoDA
F2	QSTTLKR	GCA	-	CoDA
F3	QKVSLKR	GTA	-	CoDA

Bos taurus (cow) Build 3.1

ZF DNA Sequence

Blast GTAGCAGGG

ZFA-unknown-60
2476 aGTAGATGACa 2486
2476 tCATCTACTGt 2486

FINGER	HELIX	TRIPLET	REFERENCE NUMBER	SOURCE
F1	EEVNLRR	GAC	-	CoDA
F2	VRHNLTR	GAT	-	CoDA
F3	QSTSLQR	GTA	-	CoDA

Bos taurus (cow) Build 3.1

ZF DNA Sequence

Blast GTAGATGAC

ZFA-unknown-61
2507 aGGAGACGGAg 2517
2507 tCCTCTGCCTc 2517

FINGER	HELIX	TRIPLET	REFERENCE NUMBER	SOURCE
F1	RPAKLVL	GGA	-	CoDA
F2	DRGNLTR	GAC	-	CoDA
F3	QGGHLKR	GGA	-	CoDA

ZF DNA Sequence

Bos taurus (cow) Build 3.1

Blast GGAGACGGA

ZFA-unknown-62
2510 aGACGGAGCCa 2520
2510 tCTGCCTCGGt 2520

FINGER	HELIX	TRIPLET	REFERENCE NUMBER	SOURCE
F1	DSPTLRR	GCC	-	CoDA
F2	QSAHLKR	GGA	-	CoDA
F3	DPSNLRR	GAC	-	CoDA

ZF DNA Sequence

Bos taurus (cow) Build 3.1

Blast GACGGAGCC

ZFA-unknown-63
2552 gGCAGAGGGAa 2562
2552 cCGTCTCCCTt 2562

FINGER	HELIX	TRIPLET	REFERENCE NUMBER	SOURCE
F1	THAHLTR	GGA	-	CoDA
F2	RQDNLGR	GAG	-	CoDA
F3	QSNVLSR	GCA	-	CoDA

ZF DNA Sequence

Bos taurus (cow) Build 3.1

Blast GCAGAGGGA

ZFA-unknown-64
2615 aGCTGGAGCTt 2625
2615 tCGACCTCGAa 2625

FINGER	HELIX	TRIPLET	REFERENCE NUMBER	SOURCE
F1	TTQALRR	GCT	-	CoDA
F2	QSAHLKR	GGA	-	CoDA
F3	LKHDLRR	GCT	-	CoDA

ZF DNA Sequence

Bos taurus (cow) Build 3.1

Blast GCTGGAGCT

ZFA-unknown-65
2691 aGTCGAAGTGa 2681
2691 tCAGCTTCACt 2681

FINGER	HELIX	TRIPLET	REFERENCE NUMBER	SOURCE
F1	RTSSLKR	GTG	-	CoDA
F2	QQTNLTR	GAA	-	CoDA
F3	DATQLVR	GTC	-	CoDA

ZF DNA Sequence Bos taurus (cow) Build 3.1

Blast GTCGAAGTG

ZFA-unknown-66
2744 tTAAGTAAACa 2734
2744 aATTCATTTGt 2734

FINGER	HELIX	TRIPLET	REFERENCE NUMBER	SOURCE
F1	GGTALRM	AAC	-	CoDA
F2	QRSSLVR	GTA	-	CoDA
F3	QQGNLQL	TAA	-	CoDA

ZF DNA Sequence Bos taurus (cow) Build 3.1

Blast TAAGTAAAC

ZFA-unknown-67
2792 tGGAGGAAACa 2802
2792 aCCTCCTTTGt 2802

FINGER	HELIX	TRIPLET	REFERENCE NUMBER	SOURCE
F1	GGTALVM	AAC	-	CoDA
F2	QSAHLKR	GGA	-	CoDA
F3	QMSHLKR	GGA	-	CoDA

ZF DNA Sequence Bos taurus (cow) Build 3.1

Blast GGAGGAAAC

ZFA-unknown-68
2903 aTAAGCAAGGa 2913
2903 tATTCGTTCCt 2913

FINGER	HELIX	TRIPLET	REFERENCE NUMBER	SOURCE

F1	RNEHLKV	AGG	-	CoDA
F2	QSTTLKR	GCA	-	CoDA
F3	QGGNLAL	TAA	-	CoDA

ZF DNA Sequence

Bos taurus (cow) Build 3.1

Blast TAAGCAAGG

ZFA-unknown-69
2911 gGAAGGAGGTg 2921
2911 cCTTCCTCCAc 2921

FINGER	HELIX	TRIPLET	REFERENCE NUMBER	SOURCE
F1	IPNHLAR	GGT	-	CoDA
F2	QSAHLKR	GGA	-	CoDA
F3	LGENLRR	GAA	-	CoDA

ZF DNA Sequence

Bos taurus (cow) Build 3.1

Blast GAAGGAGGT

ZFA-unknown-70
2914 aGGAGGTGAAg 2924
2914 tCCTCCACTTc 2924

FINGER	HELIX	TRIPLET	REFERENCE NUMBER	SOURCE
F1	RKPNLLR	GAA	-	CoDA
F2	EAHHLSR	GGT	-	CoDA
F3	QNSHLRR	GGA	-	CoDA

ZF DNA Sequence

Bos taurus (cow) Build 3.1

Blast GGAGGTGAA

ZFA-unknown-71
2917 aGGTGAAGGGa 2927
2917 tCCACTTCCCt 2927

FINGER	HELIX	TRIPLET	REFERENCE NUMBER	SOURCE
F1	KRERLDR	GGG	-	CoDA
F2	QQTNLTR	GAA	-	CoDA
F3	IRHHLKR	GGT	-	CoDA

ZF DNA Sequence

Bos taurus (cow) Build 3.1

Blast GGTGAAGGG

ZFA-unknown-72
2925 gGAGGGAGAAg 2935
2925 cCTCCCTCTTc 2935

FINGER	HELIX	TRIPLET	REFERENCE NUMBER	SOURCE
F1	QRSNLAR	GAA	-	CoDA
F2	QSAHLKR	GGA	-	CoDA
F3	VHWNLMR	GAG	-	CoDA

ZF DNA Sequence
Bos taurus (cow) Build 3.1
Blast GAGGGAGAA

ZFA-unknown-73
2928 gGGAGAAGGAg 2938
2928 cCCTCTTCCTc 2938

FINGER	HELIX	TRIPLET	REFERENCE NUMBER	SOURCE
F1	RMERLDR	GGA	-	CoDA
F2	QQTNLTR	GAA	-	CoDA
F3	QTTHLSR	GGA	-	CoDA

ZF DNA Sequence
Bos taurus (cow) Build 3.1
Blast GGAGAAGGA

ZFA-unknown-74
2931 aGAAGGAGAGa 2941
2931 tCTTCCTCTCt 2941

FINGER	HELIX	TRIPLET	REFERENCE NUMBER	SOURCE
F1	KHSNLTR	GAG	-	CoDA
F2	QSAHLKR	GGA	-	CoDA
F3	LGENLRR	GAA	-	CoDA

ZF DNA Sequence
Bos taurus (cow) Build 3.1
Blast GAAGGAGAG

ZFA-unknown-75
2941 aGAAGCAGAGa 2951
2941 tCTTCGTCTCt 2951

FINGER	HELIX	TRIPLET	REFERENCE NUMBER	SOURCE
F1	KHSNLAR	GAG	-	CoDA
F2	QSTTLKR	GCA	-	CoDA
F3	QRNNLGR	GAA	-	CoDA

ZF DNA Sequence [Bos taurus (cow) Build 3.1]

Blast GAAGCAGAG

ZFA-unknown-76

2949 aGAGGGAGAGg 2959
2949 tCTCCCTCTCc 2959

FINGER	HELIX	TRIPLET	REFERENCE NUMBER	SOURCE
F1	KHSNLTR	GAG	-	CoDA
F2	QSAHLKR	GGA	-	CoDA
F3	VHWNLMR	GAG	-	CoDA

ZF DNA Sequence [Bos taurus (cow) Build 3.1]

Blast GAGGGAGAG

ZFA-unknown-77

2952 gGGAGAGGAAg 2962
2952 cCCTCTCCTTc 2962

FINGER	HELIX	TRIPLET	REFERENCE NUMBER	SOURCE
F1	QASNLLR	GAA	-	CoDA
F2	RQDNLGR	GAG	-	CoDA
F3	QANHLSR	GGA	-	CoDA

ZF DNA Sequence [Bos taurus (cow) Build 3.1]

Blast GGAGAGGAA

ZFA-unknown-78

2955 aGAGGAAGGAa 2965
2955 tCTCCTTCCTt 2965

FINGER	HELIX	TRIPLET	REFERENCE NUMBER	SOURCE
F1	RMERLDR	GGA	-	CoDA
F2	QQTNLTR	GAA	-	CoDA
F3	RRDNLNR	GAG	-	CoDA

ZF DNA Sequence [Bos taurus (cow) Build 3.1]

Blast GAGGAAGGA

ZFA-unknown-79

2996 aGAGGAGGGAg 3006
2996 tCTCCTCCCTc 3006

FINGER	HELIX	TRIPLET	REFERENCE NUMBER	SOURCE
F1	THAHLTR	GGA	-	CoDA
F2	RQDNLGR	GAG	-	CoDA
F3	RVDNLPR	GAG	-	CoDA

ZF DNA Sequence

Bos taurus (cow) Build 3.1

Blast GAGGAGGGA

ZFA-unknown-80

2999 gGAGGGAGAGa 3009
2999 cCTCCCTCTCt 3009

FINGER	HELIX	TRIPLET	REFERENCE NUMBER	SOURCE
F1	KHSNLTR	GAG	-	CoDA
F2	QSAHLKR	GGA	-	CoDA
F3	VHWNLMR	GAG	-	CoDA

ZF DNA Sequence

Bos taurus (cow) Build 3.1

Blast GAGGGAGAG

ZFA-unknown-81

3007 aGAGGGAGGGa 3017
3007 tCTCCCTCCCt 3017

FINGER	HELIX	TRIPLET	REFERENCE NUMBER	SOURCE
F1	KKDHLHR	GGG	-	CoDA
F2	QSAHLKR	GGA	-	CoDA
F3	VHWNLMR	GAG	-	CoDA

ZF DNA Sequence

Bos taurus (cow) Build 3.1

Blast GAGGGAGGG

ZFA-unknown-82

3011 gGAGGGAGGCa 3021
3011 cCTCCCTCCGt 3021

FINGER	HELIX	TRIPLET	REFERENCE NUMBER	SOURCE
F1	VPSKLLR	GGC	-	CoDA
F2	QSAHLKR	GGA	-	CoDA
F3	VHWNLMR	GAG	-	CoDA

ZF DNA Sequence

Bos taurus (cow) Build 3.1

Blast GAGGGAGGC

ZFA-unknown-83
3026 gGGAGAAGAGa 3036
3026 cCCTCTTCTCt 3036

FINGER	HELIX	TRIPLET	REFERENCE NUMBER	SOURCE
F1	KHSNLTR	GAG	-	CoDA
F2	QQTNLTR	GAA	-	CoDA
F3	QTTHLSR	GGA	-	CoDA

ZF DNA Sequence
Bos taurus (cow) Build 3.1
Blast GGAGAAGAG

ZFA-unknown-84
3055 aGGAGGAAGGg 3065
3055 tCCTCCTTCCc 3065

FINGER	HELIX	TRIPLET	REFERENCE NUMBER	SOURCE
F1	RPHHLDA	AGG	-	CoDA
F2	QSAHLKR	GGA	-	CoDA
F3	QMSHLKR	GGA	-	CoDA

ZF DNA Sequence
Bos taurus (cow) Build 3.1
Blast GGAGGAAGG

ZFA-unknown-85
3056 gGAGGAAGGGg 3066
3056 cCTCCTTCCCc 3066

FINGER	HELIX	TRIPLET	REFERENCE NUMBER	SOURCE
F1	KRERLDR	GGG	-	CoDA
F2	QQTNLTR	GAA	-	CoDA
F3	RRDNLNR	GAG	-	CoDA

ZF DNA Sequence
Bos taurus (cow) Build 3.1
Blast GAGGAAGGG

ZFA-unknown-86
3059 gGAAGGGGAGg 3069
3059 cCTTCCCCTCc 3069

FINGER	HELIX	TRIPLET	REFERENCE NUMBER	SOURCE
F1	RNTNLTR	GAG	-	CoDA
F2	RREHLVR	GGG	-	CoDA

F3	QDGNLGR	GAA	-	CoDA

Bos taurus (cow) Build 3.1

ZF DNA Sequence

Blast GAAGGGGAG

ZFA-unknown-87
3062 aGGGGAGGAGg 3072
3062 tCCCCTCCTCc 3072

FINGER	HELIX	TRIPLET	REFERENCE NUMBER	SOURCE
F1	RQMNLDR	GAG	-	CoDA
F2	RQDNLGR	GAG	-	CoDA
F3	RIDKLGG	GGG	-	CoDA

Bos taurus (cow) Build 3.1

ZF DNA Sequence

Blast GGGGAGGAG

ZFA-unknown-88
3064 gGGAGGAGGAa 3074
3064 cCCTCCTCCTt 3074

FINGER	HELIX	TRIPLET	REFERENCE NUMBER	SOURCE
F1	RTDRLIR	GGA	-	CoDA
F2	QSAHLKR	GGA	-	CoDA
F3	QMSHLKR	GGA	-	CoDA

Bos taurus (cow) Build 3.1

ZF DNA Sequence

Blast GGAGGAGGA

ZFA-unknown-89
3065 gGAGGAGGAAa 3075
3065 cCTCCTCCTTt 3075

FINGER	HELIX	TRIPLET	REFERENCE NUMBER	SOURCE
F1	QASNLLR	GAA	-	CoDA
F2	RQDNLGR	GAG	-	CoDA
F3	RVDNLPR	GAG	-	CoDA

Bos taurus (cow) Build 3.1

ZF DNA Sequence

Blast GAGGAGGAA

ZFA-unknown-90
3068 gGAGGAAAGGa 3078
3068 cCTCCTTTCCt 3078

FINGER	HELIX	TRIPLET	REFERENCE NUMBER	SOURCE
F1	RMAHLHA	AGG	-	CoDA
F2	QQTNLTR	GAA	-	CoDA
F3	RRDNLNR	GAG	-	CoDA

ZF DNA Sequence Bos taurus (cow) Build 3.1

Blast GAGGAAAGG

ZFA-unknown-91
3102 aGATGAAAACa 3092
3102 tCTACTTTTGt 3092

FINGER	HELIX	TRIPLET	REFERENCE NUMBER	SOURCE
F1	GASALRQ	AAC	-	CoDA
F2	QQTNLTR	GAA	-	CoDA
F3	VGSNLTR	GAT	-	CoDA

ZF DNA Sequence Bos taurus (cow) Build 3.1

Blast GATGAAAAC

ZFA-unknown-92
3128 aTGCGGAGCAc 3118
3128 tACGCCTCGTg 3118

FINGER	HELIX	TRIPLET	REFERENCE NUMBER	SOURCE
F1	KNTRLSV	GCA	-	CoDA
F2	QSAHLKR	GGA	-	CoDA
F3	QRRSLGH	TGC	-	CoDA

ZF DNA Sequence Bos taurus (cow) Build 3.1

Blast TGCGGAGCA

ZFA-unknown-93
3172 tGCAGGGGTAt 3162
3172 aCGTCCCCATa 3162

FINGER	HELIX	TRIPLET	REFERENCE NUMBER	SOURCE
F1	QQSSLLR	GTA	-	CoDA
F2	RREHLVR	GGG	-	CoDA
F3	QTATLKR	GCA	-	CoDA

ZF DNA Sequence Bos taurus (cow) Build 3.1

Blast GCAGGGGTA

ZFA-unknown-94
3376 aTGTGTGGGAc 3366
3376 tACACACCCTg 3366

FINGER	HELIX	TRIPLET	REFERENCE NUMBER	SOURCE
F1	THAHLTR	GGA	-	CoDA
F2	RREVLEN	GTG	-	CoDA
F3	QRHGLSS	TGT	-	CoDA

ZF DNA Sequence
Bos taurus (cow) Build 3.1
Blast TGTGTGGGA

ZFA-unknown-95
3387 tGTGGCTGACa 3377
3387 aCACCGACTGt 3377

FINGER	HELIX	TRIPLET	REFERENCE NUMBER	SOURCE
F1	DLSNLKR	GAC	-	CoDA
F2	QRSDLTR	GCT	-	CoDA
F3	RPDALPR	GTG	-	CoDA

ZF DNA Sequence
Bos taurus (cow) Build 3.1
Blast GTGGCTGAC

ZFA-unknown-96
3390 gTAGGCAGTCa 3400
3390 cATCCGTCAGt 3400

FINGER	HELIX	TRIPLET	REFERENCE NUMBER	SOURCE
F1	TGAVLTR	GTC	-	CoDA
F2	QSTTLKR	GCA	-	CoDA
F3	RRDGLAG	TAG	-	CoDA

ZF DNA Sequence
Bos taurus (cow) Build 3.1
Blast TAGGCAGTC

ZFA-unknown-97
3412 aGTAGTTGATa 3402
3412 tCATCAACTAt 3402

FINGER	HELIX	TRIPLET	REFERENCE NUMBER	SOURCE

F1	TGQQLRV	GAT	-	CoDA
F2	HKSSLTR	GTT	-	CoDA
F3	QSTSLQR	GTA	-	CoDA

ZF DNA Sequence

Bos taurus (cow) Build 3.1

Blast GTAGTTGAT

ZFA-unknown-98
3450 tGCCGCAGAAt 3440
3450 aCGGCGTCTTa 3440

FINGER	HELIX	TRIPLET	REFERENCE NUMBER	SOURCE
F1	QGSNLAR	GAA	-	CoDA
F2	QSTTLKR	GCA	-	CoDA
F3	DPSNLRR	GCC	-	CoDA

ZF DNA Sequence

Bos taurus (cow) Build 3.1

Blast GCCGCAGAA

ZFA-unknown-99
3444 tGCGGCAGACt 3454
3444 aCGCCGTCTGa 3454

FINGER	HELIX	TRIPLET	REFERENCE NUMBER	SOURCE
F1	EEVNLRR	GAC	-	CoDA
F2	QSTTLKR	GCA	-	CoDA
F3	RLDMLAR	GCG	-	CoDA

ZF DNA Sequence

Bos taurus (cow) Build 3.1

Blast GCGGCAGAC

ZFA-unknown-100
3447 gGCAGACTAGt 3457
3447 cCGTCTGATCa 3457

FINGER	HELIX	TRIPLET	REFERENCE NUMBER	SOURCE
F1	RSHNLRL	TAG	-	CoDA
F2	DRGNLTR	GAC	-	CoDA
F3	QGNTLTR	GCA	-	CoDA

ZF DNA Sequence

Bos taurus (cow) Build 3.1

Blast GCAGACTAG

ZFA-unknown-101
3525 aGTAGATGGTa 3535
3525 tCATCTACCAt 3535

FINGER	HELIX	TRIPLET	REFERENCE NUMBER	SOURCE
F1	MKHHLDA	GGT	-	CoDA
F2	VRHNLTR	GAT	-	CoDA
F3	QSTSLQR	GTA	-	CoDA

ZF DNA Sequence Bos taurus (cow) Build 3.1

Blast GTAGATGGT

ZFA-unknown-102
3577 tTGGGGATTCa 3567
3577 aACCCCTAAGt 3567

FINGER	HELIX	TRIPLET	REFERENCE NUMBER	SOURCE
F1	RANHLTI	TTC	-	CoDA
F2	QSAHLKR	GGA	-	CoDA
F3	RSDHLSL	TGG	-	CoDA

ZF DNA Sequence Bos taurus (cow) Build 3.1

Blast TGGGGATTC

ZFA-unknown-103
3669 gGGTGTCTGTt 3659
3669 cCCACAGACAa 3659

FINGER	HELIX	TRIPLET	REFERENCE NUMBER	SOURCE
F1	RKQHLVL	TGT	-	CoDA
F2	DHSSLKR	GTC	-	CoDA
F3	QPHHLPR	GGT	-	CoDA

ZF DNA Sequence Bos taurus (cow) Build 3.1

Blast GGTGTCTGT

ZFA-unknown-104
3672 aGTGGGTGTCt 3662
3672 tCACCCACAGa 3662

FINGER	HELIX	TRIPLET	REFERENCE NUMBER	SOURCE
F1	TRAVLRR	GTC	-	CoDA
F2	EAHHLSR	GGT	-	CoDA
F3	RKDALHV	GTG	-	CoDA

ZF DNA Sequence

Bos taurus (cow) Build 3.1

Blast GTGGGTGTC

ZFA-unknown-105
3773 aTGTGAAGCTa 3763
3773 tACACTTCGAt 3763

FINGER	HELIX	TRIPLET	REFERENCE NUMBER	SOURCE
F1	QRQALDR	GCT	-	CoDA
F2	QQTNLTR	GAA	-	CoDA
F3	QPHGLTA	TGT	-	CoDA

ZF DNA Sequence

Bos taurus (cow) Build 3.1

Blast TGTGAAGCT

ZFA-unknown-106
3902 aGTTGATGTCa 3892
3902 tCAACTACAGt 3892

FINGER	HELIX	TRIPLET	REFERENCE NUMBER	SOURCE
F1	TKKILTV	GTC	-	CoDA
F2	VRHNLTR	GAT	-	CoDA
F3	HHNSLTR	GTT	-	CoDA

ZF DNA Sequence

Bos taurus (cow) Build 3.1

Blast GTTGATGTC

ZFA-unknown-107
3905 tGCAGTTGATg 3895
3905 aCGTCAACTAc 3895

FINGER	HELIX	TRIPLET	REFERENCE NUMBER	SOURCE
F1	TGQQLRV	GAT	-	CoDA
F2	HKSSLTR	GTT	-	CoDA
F3	QGNTLTR	GCA	-	CoDA

ZF DNA Sequence

Bos taurus (cow) Build 3.1

Blast GCAGTTGAT

ZFA-unknown-108
3947 gTAAGTAGGAt 3937
3947 cATTCATCCTa 3937

FINGER	HELIX	TRIPLET	REFERENCE NUMBER	SOURCE
F1	THAHLTR	GGA	-	CoDA
F2	QRSSLVR	GTA	-	CoDA
F3	QQGNLQL	TAA	-	CoDA

ZF DNA Sequence

Bos taurus (cow) Build 3.1

Blast TAAGTAGGA

ZFA-unknown-109
3949 tGGTGCTGACa 3959
3949 aCCACGACTGt 3959

FINGER	HELIX	TRIPLET	REFERENCE NUMBER	SOURCE
F1	DLSNLKR	GAC	-	CoDA
F2	QRSDLTR	GCT	-	CoDA
F3	HGHRLKT	GGT	-	CoDA

ZF DNA Sequence

Bos taurus (cow) Build 3.1

Blast GGTGCTGAC

ZFA-unknown-110
4039 cTGTGTAGTTt 4049
4039 gACACATCAAa 4049

FINGER	HELIX	TRIPLET	REFERENCE NUMBER	SOURCE
F1	TSTRLDI	GTT	-	CoDA
F2	QRSSLVR	GTA	-	CoDA
F3	QPHGLAH	TGT	-	CoDA

ZF DNA Sequence

Bos taurus (cow) Build 3.1

Blast TGTGTAGTT

ZFA-unknown-111
4113 cTCTGAGTGTt 4123
4113 gAGACTCACAa 4123

FINGER	HELIX	TRIPLET	REFERENCE NUMBER	SOURCE
F1	RHQHLKL	TGT	-	CoDA
F2	RQDNLGR	GAG	-	CoDA
F3	QRNTLKG	TCT	-	CoDA

ZF DNA Sequence

Bos taurus (cow) Build 3.1

Blast TCTGAGTGT

ZFA-unknown-112
4122 tTGGGATTGCa 4132
4122 aACCCTAACGt 4132

FINGER	HELIX	TRIPLET	REFERENCE NUMBER	SOURCE
F1	RKRNLIM	TGC	-	CoDA
F2	VRHNLTR	GAT	-	CoDA
F3	RSDHLSL	TGG	-	CoDA

ZF DNA Sequence
Bos taurus (cow) Build 3.1
Blast TGGGATTGC

ZFA-unknown-113
4243 tTGTGTTTGTt 4233
4243 aACACAAACAa 4233

FINGER	HELIX	TRIPLET	REFERENCE NUMBER	SOURCE
F1	RRQHLQY	TGT	-	CoDA
F2	HKSSLTR	GTT	-	CoDA
F3	QPHGLAH	TGT	-	CoDA

ZF DNA Sequence
Bos taurus (cow) Build 3.1
Blast TGTGTTTGT

ZFA-unknown-114
4254 cTAAGAAGTCt 4264
4254 gATTCTTCAGa 4264

FINGER	HELIX	TRIPLET	REFERENCE NUMBER	SOURCE
F1	TSTLLNR	GTC	-	CoDA
F2	QQTNLTR	GAA	-	CoDA
F3	QGGNLAL	TAA	-	CoDA

ZF DNA Sequence
Bos taurus (cow) Build 3.1
Blast TAAGAAGTC

ZFA-unknown-115
4379 cTTTGTTTGTa 4389
4379 gAAACAAACAt 4389

FINGER	HELIX	TRIPLET	REFERENCE NUMBER	SOURCE
F1	RRQHLQY	TGT	-	CoDA
F2	HKSSLTR	GTT	-	CoDA

F3	QRNALSG	TTT	-	CoDA

Bos taurus (cow) Build 3.1

ZF DNA Sequence

Blast TTTGTTTGT

ZFA-unknown-116
4474 aGAGGCAGGTt 4484
4474 tCTCCGTCCAa 4484

FINGER	HELIX	TRIPLET	REFERENCE NUMBER	SOURCE
F1	TTTKLAI	GGT	-	CoDA
F2	QSTTLKR	GCA	-	CoDA
F3	RGDNLNR	GAG	-	CoDA

Bos taurus (cow) Build 3.1

ZF DNA Sequence

Blast GAGGCAGGT

ZFA-unknown-117
4523 tTGTGCCGTGc 4533
4523 aACACGGCACg 4533

FINGER	HELIX	TRIPLET	REFERENCE NUMBER	SOURCE
F1	RTSSLKR	GTG	-	CoDA
F2	DSSVLRR	GCC	-	CoDA
F3	QAHGLTA	TGT	-	CoDA

Bos taurus (cow) Build 3.1

ZF DNA Sequence

Blast TGTGCCGTG

ZFA-unknown-118
4628 aTGGGTAGCAt 4638
4628 tACCCATCGTa 4638

FINGER	HELIX	TRIPLET	REFERENCE NUMBER	SOURCE
F1	HNGTLKR	GCA	-	CoDA
F2	QRSSLVR	GTA	-	CoDA
F3	RSDHLSL	TGG	-	CoDA

Bos taurus (cow) Build 3.1

ZF DNA Sequence

Blast TGGGTAGCA

ZFA-unknown-119
4669 tGATGGGTGTg 4659
4669 aCTACCCACAc 4659

FINGER	HELIX	TRIPLET	REFERENCE NUMBER	SOURCE
F1	RKQHLTL	TGT	-	CoDA
F2	RREHLVR	GGG	-	CoDA
F3	ISHNLAR	GAT	-	CoDA

ZF DNA Sequence   Bos taurus (cow) Build 3.1

Blast GATGGGTGT

ZFA-unknown-120
4672 tGGTGATGGGt 4662
4672 aCCACTACCCa 4662

FINGER	HELIX	TRIPLET	REFERENCE NUMBER	SOURCE
F1	KKDHLHR	GGG	-	CoDA
F2	VRHNLTR	GAT	-	CoDA
F3	QPHHLPR	GGT	-	CoDA

ZF DNA Sequence   Bos taurus (cow) Build 3.1

Blast GGTGATGGG

ZFA-unknown-121
4720 gTAAGAGGTTt 4710
4720 cATTCTCCAAa 4710

FINGER	HELIX	TRIPLET	REFERENCE NUMBER	SOURCE
F1	TTTVLAR	GTT	-	CoDA
F2	RQDNLGR	GAG	-	CoDA
F3	QGGNLTL	TAA	-	CoDA

ZF DNA Sequence   Bos taurus (cow) Build 3.1

Blast TAAGAGGTT

ZFA-unknown-122
4735 aGTGGCTGCTa 4725
4735 tCACCGACGAt 4725

FINGER	HELIX	TRIPLET	REFERENCE NUMBER	SOURCE
F1	MKNTLTR	GCT	-	CoDA
F2	QRSDLTR	GCT	-	CoDA
F3	RPDALPR	GTG	-	CoDA

ZF DNA Sequence   Bos taurus (cow) Build 3.1

Blast GTGGCTGCT

ZFA-unknown-123
4738 gGAAGTGGCTg 4728
4738 cCTTCACCGAc 4728

FINGER	HELIX	TRIPLET	REFERENCE NUMBER	SOURCE
F1	TKPILVR	GCT	-	CoDA
F2	RREVLEN	GTG	-	CoDA
F3	QTVNLDR	GAA	-	CoDA

ZF DNA Sequence
Bos taurus (cow) Build 3.1
Blast GAAGTGGCT

ZFA-unknown-124
4751 gGCTGGAGGAa 4741
4751 cCGACCTCCTt 4741

FINGER	HELIX	TRIPLET	REFERENCE NUMBER	SOURCE
F1	RTDRLIR	GGA	-	CoDA
F2	QSAHLKR	GGA	-	CoDA
F3	LKHDLRR	GCT	-	CoDA

ZF DNA Sequence
Bos taurus (cow) Build 3.1
Blast GCTGGAGGA

ZFA-unknown-125
4754 aGGGGCTGGAg 4744
4754 tCCCCGACCTc 4744

FINGER	HELIX	TRIPLET	REFERENCE NUMBER	SOURCE
F1	RPAKLVL	GGA	-	CoDA
F2	QRSDLTR	GCT	-	CoDA
F3	RTEHLAR	GGG	-	CoDA

ZF DNA Sequence
Bos taurus (cow) Build 3.1
Blast GGGGCTGGA

ZFA-unknown-126
4779 tGTAGAAAACa 4769
4779 aCATCTTTTGt 4769

FINGER	HELIX	TRIPLET	REFERENCE NUMBER	SOURCE

F1	GASALRQ	AAC	-	CoDA
F2	QQTNLTR	GAA	-	CoDA
F3	QSTSLQR	GTA	-	CoDA

ZF DNA Sequence
Bos taurus (cow) Build 3.1

Blast GTAGAAAAC

ZFA-unknown-127
4782 aGATGTAGAAa 4772
4782 tCTACATCTTt 4772

FINGER	HELIX	TRIPLET	REFERENCE NUMBER	SOURCE
F1	HRPNLTR	GAA	-	CoDA
F2	QRSSLVR	GTA	-	CoDA
F3	VGHNLSR	GAT	-	CoDA

ZF DNA Sequence
Bos taurus (cow) Build 3.1

Blast GATGTAGAA

ZFA-unknown-128
4785 tGTAGATGTAg 4775
4785 aCATCTACATc 4775

FINGER	HELIX	TRIPLET	REFERENCE NUMBER	SOURCE
F1	QQQALVR	GTA	-	CoDA
F2	VRHNLTR	GAT	-	CoDA
F3	QSTSLQR	GTA	-	CoDA

ZF DNA Sequence
Bos taurus (cow) Build 3.1

Blast GTAGATGTA

ZFA-unknown-129
4828 aTAGGTAGCCt 4838
4828 tATCCATCGGa 4838

FINGER	HELIX	TRIPLET	REFERENCE NUMBER	SOURCE
F1	DGSTLRR	GCC	-	CoDA
F2	QRSSLVR	GTA	-	CoDA
F3	RLDGLAG	TAG	-	CoDA

ZF DNA Sequence
Bos taurus (cow) Build 3.1

Blast TAGGTAGCC

ZFA-unknown-130
4840 tTGTGTATGTc 4850
4840 aACACATACAg 4850

FINGER	HELIX	TRIPLET	REFERENCE NUMBER	SOURCE
F1	RKQHLQL	TGT	-	CoDA
F2	QRSSLVR	GTA	-	CoDA
F3	QPHGLAH	TGT	-	CoDA

ZF DNA Sequence
Bos taurus (cow) Build 3.1
Blast TGTGTATGT

ZFA-unknown-131
4891 tGTAGCATGTa 4901
4891 aCATCGTACAt 4901

FINGER	HELIX	TRIPLET	REFERENCE NUMBER	SOURCE
F1	RRQHLQY	TGT	-	CoDA
F2	QSTTLKR	GCA	-	CoDA
F3	QKVSLKR	GTA	-	CoDA

ZF DNA Sequence
Bos taurus (cow) Build 3.1
Blast GTAGCATGT

ZFA-unknown-132
4925 tTGTGTTGGTa 4935
4925 aACACAACCAt 4935

FINGER	HELIX	TRIPLET	REFERENCE NUMBER	SOURCE
F1	RRQKLTI	GGT	-	CoDA
F2	HKSSLTR	GTT	-	CoDA
F3	QPHGLAH	TGT	-	CoDA

ZF DNA Sequence
Bos taurus (cow) Build 3.1
Blast TGTGTTGGT

ZFA-unknown-133
4975 tTGGGTTGGCa 4985
4975 aACCCAACCGt 4985

FINGER	HELIX	TRIPLET	REFERENCE NUMBER	SOURCE
F1	APSKLKR	GGC	-	CoDA
F2	HKSSLTR	GTT	-	CoDA
F3	RSDHLSL	TGG	-	CoDA

ZF DNA Sequence Bos taurus (cow) Build 3.1

Blast TGGGTTGGC

ZFA-unknown-134
5025 tTGTGTTGTTt 5035
5025 aACACAACAAa 5035

FINGER	HELIX	TRIPLET	REFERENCE NUMBER	SOURCE
F1	TKPVLKI	GTT	-	CoDA
F2	HKSSLTR	GTT	-	CoDA
F3	QPHGLAH	TGT	-	CoDA

ZF DNA Sequence Bos taurus (cow) Build 3.1

Blast TGTGTTGTT

ZFA-unknown-135
5041 aGGTGGAAACa 5031
5041 tCCACCTTTGt 5031

FINGER	HELIX	TRIPLET	REFERENCE NUMBER	SOURCE
F1	GGTALVM	AAC	-	CoDA
F2	QSAHLKR	GGA	-	CoDA
F3	IRHHLKR	GGT	-	CoDA

ZF DNA Sequence Bos taurus (cow) Build 3.1

Blast GGTGGAAAC

ZFA-unknown-136
5050 tTGTGAATAGt 5060
5050 aACACTTATCa 5060

FINGER	HELIX	TRIPLET	REFERENCE NUMBER	SOURCE
F1	RRRNLQI	TAG	-	CoDA
F2	QQTNLTR	GAA	-	CoDA
F3	QPHGLTA	TGT	-	CoDA

ZF DNA Sequence Bos taurus (cow) Build 3.1

Blast TGTGAATAG

ZFA-unknown-137
5115 aGATGTATAGg 5125
5115 tCTACATATCc 5125

FINGER	HELIX	TRIPLET	REFERENCE NUMBER	SOURCE
F1	RAHNLLL	TAG	-	CoDA
F2	QRSSLVR	GTA	-	CoDA
F3	VGHNLSR	GAT	-	CoDA

ZF DNA Sequence

Bos taurus (cow) Build 3.1

Blast GATGTATAG

ZFA-unknown-138
5123 aGGGGTAGAAt 5133
5123 tCCCCATCTTa 5133

FINGER	HELIX	TRIPLET	REFERENCE NUMBER	SOURCE
F1	HRPNLTR	GAA	-	CoDA
F2	QRSSLVR	GTA	-	CoDA
F3	RTEHLAR	GGG	-	CoDA

ZF DNA Sequence

Bos taurus (cow) Build 3.1

Blast GGGGTAGAA

ZFA-unknown-139
5205 aGGTGCAGCTc 5195
5205 tCCACGTCGAg 5195

FINGER	HELIX	TRIPLET	REFERENCE NUMBER	SOURCE
F1	TKQILGR	GCT	-	CoDA
F2	QSTTLKR	GCA	-	CoDA
F3	VDHHLRR	GGT	-	CoDA

ZF DNA Sequence

Bos taurus (cow) Build 3.1

Blast GGTGCAGCT

ZFA-unknown-140
5250 tTGTGGAGAAa 5240
5250 aACACCTCTTt 5240

FINGER	HELIX	TRIPLET	REFERENCE NUMBER	SOURCE
F1	QRSNLAR	GAA	-	CoDA
F2	QSAHLKR	GGA	-	CoDA
F3	QPHGLAH	TGT	-	CoDA

ZF DNA Sequence

Bos taurus (cow) Build 3.1

Blast TGTGGAGAA

ZFA-unknown-141
5352 tGACGCTGAGa 5362
5352 aCTGCGACTCt 5362

FINGER	HELIX	TRIPLET	REFERENCE NUMBER	SOURCE
F1	KHSNLTR	GAG	-	CoDA
F2	QRSDLTR	GCT	-	CoDA
F3	DPSNLRR	GAC	-	CoDA

ZF DNA Sequence
Bos taurus (cow) Build 3.1
Blast GACGCTGAG

ZFA-unknown-142
5378 tGTTGGTAACt 5388
5378 aCAACCATTGa 5388

FINGER	HELIX	TRIPLET	REFERENCE NUMBER	SOURCE
F1	GASALRS	AAC	-	CoDA
F2	EAHHLSR	GGT	-	CoDA
F3	IRTSLKR	GTT	-	CoDA

ZF DNA Sequence
Bos taurus (cow) Build 3.1
Blast GTTGGTAAC

ZFA-unknown-143
5434 gGGTGAAGGAc 5424
5434 cCCACTTCCTg 5424

FINGER	HELIX	TRIPLET	REFERENCE NUMBER	SOURCE
F1	RMERLDR	GGA	-	CoDA
F2	QQTNLTR	GAA	-	CoDA
F3	IRHHLKR	GGT	-	CoDA

ZF DNA Sequence
Bos taurus (cow) Build 3.1
Blast GGTGAAGGA

ZFA-unknown-144
5457 tTGTGTTGCTa 5467
5457 aACACAACGAt 5467

FINGER	HELIX	TRIPLET	REFERENCE NUMBER	SOURCE
F1	TKQILGR	GCT	-	CoDA
F2	HKSSLTR	GTT	-	CoDA

F3	QPHGLAH	TGT	-	CoDA

Bos taurus (cow) Build 3.1

ZF DNA Sequence

Blast TGTGTTGCT

ZFA-unknown-145
5465 cTAAGTTGTAa 5475
5465 gATTCAACATt 5475

FINGER	HELIX	TRIPLET	REFERENCE NUMBER	SOURCE
F1	QQQALKR	GTA	-	CoDA
F2	HKSSLTR	GTT	-	CoDA
F3	QQGNLQL	TAA	-	CoDA

Bos taurus (cow) Build 3.1

ZF DNA Sequence

Blast TAAGTTGTA

ZFA-unknown-146
5638 gTAAGCAGCAa 5628
5638 cATTCGTCGTt 5628

FINGER	HELIX	TRIPLET	REFERENCE NUMBER	SOURCE
F1	QRGTLNR	GCA	-	CoDA
F2	QSTTLKR	GCA	-	CoDA
F3	QGGNLAL	TAA	-	CoDA

Bos taurus (cow) Build 3.1

ZF DNA Sequence

Blast TAAGCAGCA

ZFA-unknown-147
5699 aTAGGGAGAAa 5689
5699 tATCCCTCTTt 5689

FINGER	HELIX	TRIPLET	REFERENCE NUMBER	SOURCE
F1	QRSNLAR	GAA	-	CoDA
F2	QSAHLKR	GGA	-	CoDA
F3	RPEGLST	TAG	-	CoDA

Bos taurus (cow) Build 3.1

ZF DNA Sequence

Blast TAGGGAGAA

ZFA-unknown-148
5709 aGAAGAAAACa 5699
5709 tCTTCTTTTGt 5699

FINGER	HELIX	TRIPLET	REFERENCE NUMBER	SOURCE
F1	GASALRQ	AAC	-	CoDA
F2	QQTNLTR	GAA	-	CoDA
F3	QTNNLNR	GAA	-	CoDA

ZF DNA Sequence

Bos taurus (cow) Build 3.1

Blast GAAGAAAAC

ZFA-unknown-149

5806 tGCAGCTGTCc 5816

5806 aCGTCGACAGg 5816

FINGER	HELIX	TRIPLET	REFERENCE NUMBER	SOURCE
F1	TRAVLRR	GTC	-	CoDA
F2	QRSDLTR	GCT	-	CoDA
F3	QGGTLRR	GCA	-	CoDA

ZF DNA Sequence

Bos taurus (cow) Build 3.1

Blast GCAGCTGTC

ZFA-unknown-150

5824 tGGTGCTGGGa 5814

5824 aCCACGACCCt 5814

FINGER	HELIX	TRIPLET	REFERENCE NUMBER	SOURCE
F1	KKDHLHR	GGG	-	CoDA
F2	QRSDLTR	GCT	-	CoDA
F3	HGHRLKT	GGT	-	CoDA

ZF DNA Sequence

Bos taurus (cow) Build 3.1

Blast GGTGCTGGG

ZFA-unknown-151

5824 aTTTGTTGAAg 5834

5824 tAAACAACTTc 5834

FINGER	HELIX	TRIPLET	REFERENCE NUMBER	SOURCE
F1	QRSNLAR	GAA	-	CoDA
F2	HKSSLTR	GTT	-	CoDA
F3	QRNALSG	TTT	-	CoDA

ZF DNA Sequence

Bos taurus (cow) Build 3.1

Blast TTTGTTGAA

ZFA-unknown-152
5827 tGTTGAAGAGa 5837
5827 aCAACTTCTCt 5837

FINGER	HELIX	TRIPLET	REFERENCE NUMBER	SOURCE
F1	KHSNLTR	GAG	-	CoDA
F2	QQTNLTR	GAA	-	CoDA
F3	VGGSLNR	GTT	-	CoDA

ZF DNA Sequence
Bos taurus (cow) Build 3.1
Blast GTTGAAGAG

ZFA-unknown-153
5874 aGAAGGGTGTc 5864
5874 tCTTCCCACAg 5864

FINGER	HELIX	TRIPLET	REFERENCE NUMBER	SOURCE
F1	RKQHLTL	TGT	-	CoDA
F2	RREHLVR	GGG	-	CoDA
F3	QDGNLGR	GAA	-	CoDA

ZF DNA Sequence
Bos taurus (cow) Build 3.1
Blast GAAGGGTGT

ZFA-unknown-154
6080 tTAGGATTAGg 6090
6080 aATCCTAATCc 6090

FINGER	HELIX	TRIPLET	REFERENCE NUMBER	SOURCE
F1	RSHNLRL	TAG	-	CoDA
F2	VRHNLTR	GAT	-	CoDA
F3	RIDHLVP	TAG	-	CoDA

ZF DNA Sequence
Bos taurus (cow) Build 3.1
Blast TAGGATTAG

ZFA-unknown-155
6224 gGTAGTTGCTa 6234
6224 cCATCAACGAt 6234

FINGER	HELIX	TRIPLET	REFERENCE NUMBER	SOURCE

F1	TKQILGR	GCT	-	CoDA
F2	HKSSLTR	GTT	-	CoDA
F3	QSTSLQR	GTA	-	CoDA

ZF DNA Sequence Bos taurus (cow) Build 3.1

Blast GTAGTTGCT

ZFA-unknown-156
6269 tGCAGTGGAAc 6259
6269 aCGTCACCTTg 6259

FINGER	HELIX	TRIPLET	REFERENCE NUMBER	SOURCE
F1	HKPNLHR	GAA	-	CoDA
F2	RREVLEN	GTG	-	CoDA
F3	QGGTLRR	GCA	-	CoDA

ZF DNA Sequence Bos taurus (cow) Build 3.1

Blast GCAGTGGAA

ZFA-unknown-157
6297 tGTTGCCTGTa 6307
6297 aCAACGGACAt 6307

FINGER	HELIX	TRIPLET	REFERENCE NUMBER	SOURCE
F1	RKQHLTL	TGT	-	CoDA
F2	DSSVLRR	GCC	-	CoDA
F3	IRTSLKR	GTT	-	CoDA

ZF DNA Sequence Bos taurus (cow) Build 3.1

Blast GTTGCCTGT

ZFA-unknown-158
6387 aGCTGAAGACt 6397
6387 tCGACTTCTGa 6397

FINGER	HELIX	TRIPLET	REFERENCE NUMBER	SOURCE
F1	DEANLRR	GAC	-	CoDA
F2	QQTNLTR	GAA	-	CoDA
F3	VGNSLTR	GCT	-	CoDA

ZF DNA Sequence Bos taurus (cow) Build 3.1

Blast GCTGAAGAC

ZFA-unknown-159
6427 tGAGGAAGACa 6417
6427 aCTCCTTCTGt 6417

FINGER	HELIX	TRIPLET	REFERENCE NUMBER	SOURCE
F1	DEANLRR	GAC	-	CoDA
F2	QQTNLTR	GAA	-	CoDA
F3	RRDNLNR	GAG	-	CoDA

ZF DNA Sequence Bos taurus (cow) Build 3.1

Blast GAGGAAGAC

ZFA-unknown-160
6430 tGGTGCAGCTa 6440
6430 aCCACGTCGAt 6440

FINGER	HELIX	TRIPLET	REFERENCE NUMBER	SOURCE
F1	TKQILGR	GCT	-	CoDA
F2	QSTTLKR	GCA	-	CoDA
F3	VDHHLRR	GGT	-	CoDA

ZF DNA Sequence Bos taurus (cow) Build 3.1

Blast GGTGCAGCT

ZFA-unknown-161
6452 cTGTGTGGCTg 6462
6452 gACACACCGAc 6462

FINGER	HELIX	TRIPLET	REFERENCE NUMBER	SOURCE
F1	TKPILVR	GCT	-	CoDA
F2	RREVLEN	GTG	-	CoDA
F3	QRHGLSS	TGT	-	CoDA

ZF DNA Sequence Bos taurus (cow) Build 3.1

Blast TGTGTGGCT

ZFA-unknown-162
6455 tGTGGCTGTTa 6465
6455 aCACCGACAAt 6465

FINGER	HELIX	TRIPLET	REFERENCE NUMBER	SOURCE
F1	QATLLRR	GTT	-	CoDA
F2	QRSDLTR	GCT	-	CoDA
F3	RPDALPR	GTG	-	CoDA

ZF DNA Sequence

Bos taurus (cow) Build 3.1

Blast GTGGCTGTT

ZFA-unknown-163
6703 aGGGGAGAACt 6693
6703 tCCCCTCTTGa 6693

FINGER	HELIX	TRIPLET	REFERENCE NUMBER	SOURCE
F1	GHTALRN	AAC	-	CoDA
F2	RQDNLGR	GAG	-	CoDA
F3	RIDKLGG	GGG	-	CoDA

ZF DNA Sequence

Bos taurus (cow) Build 3.1

Blast GGGGAGAAC

ZFA-unknown-164
6710 gGTAGCAAGGg 6700
6710 cCATCGTTCCc 6700

FINGER	HELIX	TRIPLET	REFERENCE NUMBER	SOURCE
F1	RNEHLKV	AGG	-	CoDA
F2	QSTTLKR	GCA	-	CoDA
F3	QKVSLKR	GTA	-	CoDA

ZF DNA Sequence

Bos taurus (cow) Build 3.1

Blast GTAGCAAGG

ZFA-unknown-165
6729 aGCAGAATAGt 6719
6729 tCGTCTTATCa 6719

FINGER	HELIX	TRIPLET	REFERENCE NUMBER	SOURCE
F1	RRRNLQI	TAG	-	CoDA
F2	QQTNLTR	GAA	-	CoDA
F3	QGNTLTR	GCA	-	CoDA

ZF DNA Sequence

Bos taurus (cow) Build 3.1

Blast GCAGAATAG

ZFA-unknown-166
6791 aTGGGAATGTg 6801
6791 tACCCTTACAc 6801

FINGER	HELIX	TRIPLET	REFERENCE NUMBER	SOURCE
F1	RRQHLQY	TGT	-	CoDA
F2	QQTNLTR	GAA	-	CoDA
F3	RSDHLSL	TGG	-	CoDA

ZF DNA Sequence

Bos taurus (cow) Build 3.1

Blast TGGGAATGT

ZFA-unknown-167

6799 gTGGGAATGCc 6809

6799 cACCCTTACGg 6809

FINGER	HELIX	TRIPLET	REFERENCE NUMBER	SOURCE
F1	RRRNLTL	TGC	-	CoDA
F2	QQTNLTR	GAA	-	CoDA
F3	RSDHLSL	TGG	-	CoDA

ZF DNA Sequence

Bos taurus (cow) Build 3.1

Blast TGGGAATGC

ZFA-unknown-168

6863 tTGTGATTGCa 6873

6863 aACACTAACGt 6873

FINGER	HELIX	TRIPLET	REFERENCE NUMBER	SOURCE
F1	RKRNLIM	TGC	-	CoDA
F2	VRHNLTR	GAT	-	CoDA
F3	QPHGLAH	TGT	-	CoDA

ZF DNA Sequence

Bos taurus (cow) Build 3.1

Blast TGTGATTGC
