# Supplementary material for: Targeting wild-type Erythrocyte receptors for Plasmodium falciparum and vivax Merozoites by Zinc Finger Nucleases In- silico: Towards a Genetic Vaccine against Malaria
Source: Genet Vaccines Ther. 2012 Aug 31;10:8. doi: 10.1186/1479-0556-10-8 (PMC3500210; doi:10.1186/1479-0556-10-8)
Supplement: Additional file 3 — A list of the 1–6 recognition domains of the alpha-helix of one of the paired ZFAs for engineering ZFNs that cleave the human darc-gene. This file offers a detailed list of the 1–6 recognition domains of the alpha-helix of one of the paired ZFAs for engineering ZFNs that cleave the human darc-gene. [file 1479-0556-10-8-S3.pdf]

Zinc Finger Site Type: Nuclease
Zinc Finger Engineering Method: CoDA
Sequence Name : darc
Sequence Length:2.49kb
Nucleotide Sequence
:nTTTTTCCTGAGTGTAGTCCCAACCAGCCAAATCCAACCTCAAAACAGGAAG
ACCCAAGGCCAGTGACCCCCATAGGCCTGAGGCTTGTGCAGGCAGTGGGC
GTGGGGTAAGGCTTCCTGATGCCCCCTGTCCCTGCCCAGAACCTGATGGC
CCTCATTAGTCCTTGGCTCTTATCTTGGAAGCACAGGCGCTGACAGCCGTC
CCAGCCCTTCTGTCTGCGGGCCTGAACCAAACGGTGCCATGGGGAACTGT
CTGCACAGGGTGAGTATGGGGCCAGGCCCCAGAGTCCCTTATCCCTATGC
CCCTCATTTCCCGTGCTGTTTGCCCCTCAGTCTTTATATCTCTTCCTTTTCCT
CCTCATCTTTTCTCCCTTCCTGCTTTTTTCCTCTTCCTTCAAAGTCTTTTTCCT
TCTCTCCTTCCTATGCTAGCCTCCTAGCTCCCTCTTGTGTCCCTCCCTTTGC
CTTTGAGTCAGTTCCATCCTGGTCTCTTGGTGCCTTTTCCTTCTGACCTTGC
ACTGCTCCTCCAGCCCCAGCTGCCCTGGCTTCCCCAGGACTGTTCCTGCTC
CGGCTCTTCAGGCTCCCTGCTTTGTCCTTTTCCACTGTCCGCACTGCATCTG
ACTCCTGCAGAGACCTTGTTCTCCCACCCGACCTTCCTCTCTGTCCTCCCCT
CCCACCTGCCCCTCAATTCCCAGGAGACTCTTCCGGTGTAACTCTGATGGC
CTCCTCTGGGTATGTCCTCCAGGCGGAGCTCTCCCCCTCAACTGAGAACTC
AAGTCAGCTGGACTTCGAAGATGTATGGAATTCTTCCTATGGTGTGAATGAT
TCCTTCCCAGATGGAGACTATGGTGCCAACCTGGAAGCAGCTGCCCCCTGC
CACTCCTGTAACCTGCTGGATGACTCTGCACTGCCCTTCTTCATCCTCACCA
GTGTCCTGGGTATCCTAGCTAGCAGCACTGTCCTCTTCATGCTTTTCAGACC
TCTCTTCCGCTGGCAGCTCTGCCCTGGCTGGCCTGTCCTGGCACAGCTGG
CTGTGGGCAGTGCCCTCTTCAGCATTGTGGTGCCCGTCTTGGCCCCAGGG
CTAGGTAGCACTCGCAGCTCTGCCCTGTGTAGCCTGGGCTACTGTGTCTGG
TATGGCTCAGCCTTTGCCCAGGCTTTGCTGCTAGGGTGCCATGCCTCCCTG
GGCCACAGACTGGGTGCAGGCCAGGTCCCAGGCCTCACCCTGGGGCTCAC
TGTGGGAATTTGGGGAGTGGCTGCCCTACTGACACTGCCTGTCACCCTGGC
CAGTGGTGCTTCTGGTGGACTCTGCACCCTGATATACAGCACGGAGCTGAA
GGCTTTGCAGGCCACACACACTGTAGCCTGTCTTGCCATCTTTGTCTTGTTG
CCATTGGGTTTGTTTGGAGCCAAGGGGCTGAAGAAGGCATTGGGTATGGG
GCCAGGCCCCTGGATGAATATCCTGTGGGCCTGGTTTATTTTCTGGTGGCC
TCATGGGGTGGTTCTAGGACTGGATTTCCTGGTGAGGTCCAAGCTGTTGCT
GTTGTCAACATGTCTGGCCCAGCAGGCTCTGGACCTGCTGCTGAACCTGGC
AGAAGCCCTGGCAATTTTGCACTGTGTGGCTACGCCCCTGCTCCTCGCCCT
ATTCTGCCACCAGGCCACCCGCACCCTCTTGCCCTCTCTGCCCCTCCCTGA
AGGATGGTCTTCTCATCTGGACACCCTTGGAAGCAAATCCTAGTTCTCTTCC
CACCTGTCAACCTGAATTAAAGTCTACACTGCCTTTGTGAAn
Selected Module Sets:
Left Module Count: 3
Spacer Nucleotide Count: 5,6,7
Right Module Count: 3
Ignore Asp Overlap: False

The results below are zinc finger arrays that can be constructed using CoDA. Note that other

methods (including modular assembly and OPEN) can also potentially be used to target the input sequence of interest.”

Sort By: Position ☐ **Hide intron splice sites**

⊞ ZFN-unknown-SP-5-1
1038 tGCCCTCTTCAGCATTGTGGTGCCc 1062
1038 aCGGGAGAAGTCGTAACACCACGGg 1062

FINGER	HELIX	TRIPLET	REFERENCE NUMBER	SOURCE
Left F1	SPSKLVR	GGC	-	CoDA
Left F2	RQDNLGR	GAG	-	CoDA
Left F3	QRNNLGR	GAA	-	CoDA
Right F1	SKKSLTR	GCC	-	CoDA
Right F2	EAHHLSR	GGT	-	CoDA
Right F3	QPHGLAH	TGT	-	CoDA

ZF DNA Sequence Bos taurus (cow) Build 3.1

Blast GCCCTCTTCNNNNNTGTGGTGCC

⊞ ZFN-unknown-SP-7-1
1337 tGCAGGCCACACACACTGTAGCCTGTc 1363
1337 aCGTCCGGTGTGTGTGACATCGGACAg 1363
