# Supplementary material for: Targeting wild-type Erythrocyte receptors for Plasmodium falciparum and vivax Merozoites by Zinc Finger Nucleases In- silico: Towards a Genetic Vaccine against Malaria
Source: Genet Vaccines Ther. 2012 Aug 31;10:8. doi: 10.1186/1479-0556-10-8 (PMC3500210; doi:10.1186/1479-0556-10-8)
Supplement: Additional file 4 — A list of the 1–6 recognition domains of the alpha-helix of one of the paired ZFAs for engineering ZFNs that cleave the human glycophorin-a gene. This file offers details of the 1–6 recognition domians (denoted F1, F2, F3/F3, F2, F1) of the alpha-helix for the 18 assembled paired ZFAs for engineering ZFNs that cleave within the human glycophorin-a gene. [file 1479-0556-10-8-S4.pdf]

Zinc Finger Site Type: Nuclease
Zinc Finger Engineering Method: CoDA
Sequence Name : GI|224589816:C145061904-145030456_HOMO_SAPIENS_CHROMOSOME_4,_GRCH37.P2_PRIMARY_REFERENCE_ASSEMBLY
Sequence Length:31451
Nucleotide Sequence
:nGCAGGAAGGTGGGCCTGGAAGATAACAGCTAGCAGGCTAAGGTCAGACA
CTGACACTTGCAGTTGTCTTTGGTAGTTTTTTTGCACTAACTTCAGGAACCA
GCTCATGATCTCAGGATGTATGGAAAAATAATCTTTGTATTACTATTGTCAGG
TAAGTGATTTTATTTCATCTTGGTTCTGTTATATTGGGTATGAGATCATAGAA
TAAAATATGAACTACCCTATTTTAGTTCTATCTTATTTAAATCAATAAATGAGT
AGTATTTCCTCTTCCAGTCTGGTGGATGGATTTTACTGGAACTCAGCTACCA
ATGTGGGGGAAATGGCACAAGGGAGCCCAGTATTTATGGCCAAATCCAGTT
TTCTAGTATGAGAAGCTTACTTCAATTCTAAGTCTAGCTAGAATTAAAATAAT
TTTATCAAATGCTATGAGAAATACCTCTCTGTGAATAAATGTATTGCTTTGTTT
GAGTTATAAGGAGATTCATTTCCAAACTAAAGAGTTATTAACGAAGATGTTG
GTAGCTATATGGCTTTTAGTTTTCAAAAGGTATAATTTCCTATTTCTGCCAAA
TGGCGAGAAGCCAAAAGCATGAACACTGAAACCGTGGGGAGTTGTTCGCTT
CTCTGTGGGTCCATTACTAAAGTGTCACATAGGAAGAAAAAAAACAAAAACA
ACTCTTACTGGCTTAGGTATCCTGTGAATTTTAGGAGAAATTTAAATCCATTA
AAATAAAGAAATATCATAGGGTTATTATTAAATTGTATTAATTCAATAATTTGA
ATTTAACTTAGTTTAAATTTAATTATTAATTTAGTGTCTTAAATTAACATGATTT
TGGCCTCTTTCTGAGAATATTATAGTTAAACATCCTCTCAAGTGCAGTGCTTA
TGTGTTAGCAATACTAGTGCCCAGCACACAGCGGGCAGGCAGTTGCTTGAA
ACATTCTGAGTCTATTAGACATTGCTGTATCCCAAGTGAGAGCAAGTATCAA
GGAGCTACTGAGCACTCTGTAGCACACAGGGAGGAGAGATCAGCATTTTCT
AAGATACCCTAGGGGAGGATAAAATAGTGCAATAGTTAAGAGCACAGGCAT
GAGGAACAGACAGAACTGGGTTCAAATCTACTTTTACTTCTCAAGGCTGGG
GAACATTAAGGCAAATTATGTGCCCACATTTTTATGTGTCCTCGTCTTTAAAA
TGCAGGCAGTGTTGGTACTTACCTCATAATAATTGCATAAAGATTAAACAAAA
TATTTAATGGAATACACTTACTGATGCCTGAAACAAAGTAAAATGTTAAGATT
ACTATGCATTTTCTGTGATTAGAATTAACTATCATGATTAAAAAGTATTAATAA
TATATTATTAAAATAAGCAGTAGCTATCAATAGTTACAGACTAGGGAACAAAC
CTACGTATGTGATTGGTGATTTCTGAAAAGTCAGAGAGAAAAGAAAATTACA
GAAAGAAAACAGAAAACAAACATAGCTACTCTAATTTTTTAAGCAGAAAAGTA
TGAAAACATTTAGTTTGAAGAAAAGAAAACAAATGAAAGGGATGTAGTGTAA
TATTTGTATATATATTCATATATTTGAAGTGCTATTACACAGAAAAAAAGATGT
ATTCTTTGTGTTGCTCCATGGGGCAAACCAAACTGGATGTAACTCAAGCAAA
ATTAGACACTGCATACTCTACTGGGGGTGTGCCCAGCATTTGGGAAAACTC
TGTGTGACTTACAAGTGCCCCAAATTTGGAAAGGGTTCCTGGCAAAGAAAT
GATTTTTTTTTAAATTTCTACAACTACACAAGCAGATAGTGTATTAAAGCCTT
AAATGGCACTTGGTCACTGGGGCAAGATGACCCTGAAAGCTACAATGGTCT
CCAGTACCCAAGCTGTTATCATCTTTGTAGCTTCAGAAACCCTCCAAGGAAA
CTCTCTTGATGTGGCTACTTTATAGTATAACAGAAAGGTGTAAGATCAAGTTT
TTCCCCCATACTGATTAGCTGAAGAGTAAACATGGTGAAGTCTTTTTCTTTTT

CTTTTATGTTGCTATAAAAAAAAAGATGATTGCCTTGCTTTCTCCAGGAATCT
TAAGAATAAAGCCAATATTTCTAATTCTAAACTTACCAGAGATCTCCTTCCAA
ATGGAGAATCCATTTTTTCTAATATGACTTGATTCCCAGTCCCTGAATTCCTG
CACTCATTTGATGATTCAGTCATTACATGTCAGATTGTGAACCAGACACTGA
GCCCACAGCAGGAAGAAAAATGGGCTCCCATGGAGGATACACGGAGGGTA
GGCGCAGTGGATGATGGGAGGGAACGCAGATAATAAATGGAACAACAACTA
TCTTATTAAAATAAGATAAAAACAGTCAAAACTAATACAAAGCATATAAAACC
AGGTAAGATGATAAACATGAATGCCGAAAGCTGCTTAAGAAAAGGGTAGCA
GGGAGTTATTTTCTGAGTAGATGACATTTATGCTAAATGTGGAACAAGGAGA
CGGAGCCAACCCTGAAAATTCTGGGAAAAGAGGACAGAAGGCAGAGGGAA
GAGCAAGAGCAAAAATTCTGAAACAGCAGGTAAGTTAGTGTTTTCAAGGAAA
AGCTGGAGCTTTTATCTGAAAATCAGATTCTGAAGCTAAGAACCAATTTGAA
AATACAATACAATATCACTTCGACTAGGAAATTATGGCATAAACCAGGAGTC
TCCAAAAGCTTTTTGTGTTTACTTAAAAATTCATACAAAATTTGCATTCTAGGT
CATAATATACTAATTTAATTGGAGGAAACAAAGGCACTGGTATGATATCATCA
TGCCTACTTTATTCATCCGTGTATCCCCAGAATCTAGCACAGTTCCCGATTG
GTATTTATAGTAGCATATTGGTTGAATAAGCAAGGAAGGAGGTGAAGGGAG
GGAGAAGGAGAGAGAAGCAGAGAGGGAGAGGAAGGAAGAAAGAAAAGGAA
AAAGGGAAGGAAAGAAGAGAGGAGGGAGAGAGGGAGGGAGGCAAGAAGG
GAGAAGAGAGAAGGGAAGGGAAGAGACAGGAGGAAGGGGAGGAGGAAAG
GAAAGAGGAAATATTTGTTTTCATCTGGTTAGACACAGTGAGTGCTCCGCAT
AGACAGATCATTATTACCCTGTGCATCTGACTCATACCCCTGCAAGTACATC
AGTCTGAGAAGCACATGTTAAGTGAAGAAACAAGGCATCTCTTTTTTTTTTT
TTTTCAGGGATCCAAGAAGAGAGCCTTGCTAGCTGCTATTTAATTGGCACAG
GAAAGAGTTACAGGAACTGTATGCCAGGGAATACATGACTATAAATTCTTTA
AAAGCAAAACCTGTGTCTTCGCTTATGTGTCCCACACATTGTCAGCCACATA
GTAGGCAGTCAATATCAACTACTCAAAATGACAAATGACAAATGACCAGAAT
TCTGCGGCAGACTAGTTTAGCCATGAAAAATCATTTAACACCCGTGGGCCTC
AGTTTTCTTGTGCCTATTCAATAAAGCGCCGAGTAGATGGTATCTACAAGCA
TTTTTCAACTGTAAACCCCAATGAATCCCCAAAATTCAGCCTGAGATGAGCT
GGACTAGTTGCCAAACCTATAAATATCTTTAGCATGGTGTGAAATAGGGTTT
TTAGAAAGAAACAGACACCCACTGTGAACTCCTTTGCAGAAAAGGTCTGAAT
AGAGGGGAAAGTAGGGATGGTATCTCAAACTTACTTTGTAGTGATTTTAAAT
TAGGAAATTTAGCTTCACATTCTTGTGATAAATTTCTTTTCACCTTGGTTTCTA
GAAGATTATTCAAAACATCTGTGAGACTATTTGAGAAGTATACTTTTGGGGA
ATTTCCCCAAGTTATCTTTATAGATTATATTTTGACATCAACTGCAAATGTAAT
ATCTTTTACTCAAAAAAAACCCAATCCTACTTACATGGTGCTGACAAAATCAG
GCTGGACCTACATTTTTACATCATAGATTTCCAGCCATTATTATCATATCCAC
ATCTTTAGTAAGTACCTATCTGTGTAGTTTTCTGTGATAAATGAACTAAACTA
AAACTAAAGCAAAAATGTTGAAAAAAAAATTCCAGGTTTATCTCTGAGTGTTGG
GATTGCAAGGTTTTTTTTTCTCATTTTAAATACTTTCTAAATTTTCTGCAAAGA
GAACCATATAATCTAATCAGGACAAGTTTTAATATATTTTAAAAAGTAAACCG
AACAAACACAATCTCTGCTTTCTAAGAAGTCTTTAATTTTTGTACGTTGGTCA
TAGACTATGACTATACAATTTATTTGTGATATGTATTAAGAATTTCTGTCTAAC
CCAAATTATTATATGTAAGCACGGGAAAAATGATGTCATCTTTGTTTGTAGTG
TACAAAGTTCTATAAACAGCTATTTGATCAACTTTGGTATTTCCATCCCTAGA

TTTATATACAGCAGGTTAGGTTCCATACAGAGGCAGGTTCTGAATAATAATA
ACCAACACTGATAATAGCACTTACTTTGTGCCGTGCACTGTTCTAAGCAATTT
ACATACACTTAATTTTTAAAATTGTAGTAAAATACACATAATATAAATTTACCA
TTTGAACCATTTTAAAGTGTACAATGGGTAGCATTTAATGCAGTCAAAATGAT
GCACACCCATCACCATTATGTAGCTCCAGAACATTTTCATCACTCCAAAAGG
AAACCTCTTACCCATTAGCAGCCACTTCCAATTCCTCCAGCCCCTGGAAACC
ACTAATTTGTTTTCTACATCTACAGATATACCCATTGTAGATATTTCATATAAA
TGGAATCATATAATAGGTAGCCTTTTGTGTATGTCCTCTTTCACTTAAAATAA
TGTGTTTAAAGTTCATCCATATTGTAGCATGTATCAGTATTTCATTCCTTTTAT
AATTGTGTTGGTATATCTCATTTTGTTTATCCACCCATCATTTGATTAAAATTT
GGGTTGGCATATCACATTTTGCTTATCGATCCATCATTTGATTAAAATTTGTG
TTGTTTCCACCTTTTGGCTATTGTGAATAGTGCTGCTATAAATATTCCTGTAC
TAGTTTTGTTTGAACCCACTTTTAATACTCAAAGATGTATAGGGGTAGAATTG
CTGGGTCATAGTAATTTTATGTTTAACTTACTAAGGAACTGCTCAACTCTTTT
CCACAGGAGCTGCACCTTTTGACCTTTTCACCAGGGTGTATGAGGTGCCAA
TTTCTCCACAATCTTGCCAGAAATTGTACTTTTTCATTTTTTTAATTATAGCCA
TTTCAGAGGGTATGAAATGGTTTTTCACTGTGGTTTCTTGCATTTTCCTAATA
ACTAATGACGCTGAGAATCTTCTCATGTAATTGTTGGTAACTGCATTTTGCAT
ATCTTTGGAGAAATGTTGGTACTAGTCCTTCACCCATTTTTCAATCTATTTTT
CTTTTTGTGTTGCTAAGTTGTAAGAGTTCTTTCTATGTTCTGGATAAAGAGTC
TTATCAGATATACTATTTGCAAATCTTTTCCTTCATTCTGTAGATTTTTGTTTTT
ACTTTTGATAGTGTCCTTTGATGCACAAATGTTTTTCATTTTCAAGTCCAATTT
ATTTTTTTTTCTTTTGCTGCTTACGCTTTTGATATCATATCTAAAAATAATTGC
CAAATTTAAAGTCATAAAAAATTTCTCCCTATGTTTTCTTCTAAGAGTTTTGTAT
TTCTTCTCTTATATTTAGATCTTTGGTTTATTATCAGTTAATTTTTCTATATGAT
GTATGATAAGAGTCCACCTTTATTATTTTGCAGCTGTCCCAGCACCATTTGTT
GAAGAGACTATCCTTTGCCCATTGAATGGTCTTGACACCCTTCTTGAAAGTT
AATTGGCCATGGATATATGAGTTTATTTCTGGAGTCTCAATTCTATCCTAAGA
ATATGTCTGTTCTTGGGGCAAAATCACACAGTTTTTATTGCTGTTACTTGGTT
ATACGTTTTTAATTCATGAAGTGTGATTCACCAAACTTTGTTCTTCAAGATTG
TTTTGCCTATTTAGATCCCTAACAATTTCATAGAAATTTTAGGATTAGGTTTTC
CATTCTTGCAAAAAAATAATTATGTGCATTTTAACTTAACCTGTTCAATAACTC
TATAAGGTAGAGACTAATCCATGTATAATGATGGAACAAAAATATAGAGATTA
AGTAAATTTTGCAAGGTCTCAGGTAGTTGCTAGAGGAATTAGTTTGAGCCTA
GGCAGTTCCACTGCAGAATCTGTGCACTTAGAGAATATGTCATGTTGCCTGT
ACCATACCTAGTGATGTTCCAGGATTGGCTCCTTTACTCTTACAACATTGTCA
CTCAGTGTTCTGCCTGTGCTTTCACCAAGCTGAAGACTTTAATGAAGGTTGA
CGGTCTGTCTTCCTCACGTGGTGCAGCTAAGGAACTCTAACTGTGTGGCTG
TTATGTTAGCCTTTTGCTCCTTTTTATATGGGCTATAGAAAATGTTTTTAAATC
CTGGAGGCCTCCTTTTGATGTTATCACTTATTTCCCAGTCATCACTATATTTT
TAAAAGCCAAAATAGAAGGAAATAAATACAAAACATAAAACATGAATAGTACA
GCTATTTGAGGCAACTGAGAATAGAGATCATGGCACTGAAATTGCATTTTGC
TAGGAAAAAGACCACAAAAGTTCTCCCCTTGCTACCTTTCCTGAACTATTCT
GCTAGATTCAGACTTCAAAAACATTGTATCAGGAAATACAGAAATGTTCTTTC
AAAATGAGTGTATGGGAATGTGGGAATGCCTAATAAAATCTGTCCTCATTGA
TTCGTTAGCAAAAATCATATAAATCAATACCTTGTGATTGCAAGCAGATATAT

TTCAGATCCTTTCTGTGTTTGTTTTTTTGCTTTCTTGATCTATCACAATTGGAG
AAAACTTAAAATTTCTCAATGGTATTGTATTTTTGCCAATTTCTTATTCTGCTT
TATGTTTCTCGTTGCTATATTATTGGGCTATAATGGTCCATAATTACTTAAGA
ATCACTGTGAAATATATTGCTTAATGACACAAGTAAATCTTTTTCATTGTTTGT
AATGTCTTTGCTCTTAATTCTACTTTGCCTAAGATTAATACGGTTATTCCTGTT
TAGTTTTATATGTATTTATTTATTTATTTTGAAGATGGAGTCTCGTTCTGTCGC
CCAGGCTGGAGTGCAGTTGCATGATCTCGGCTCACTGCAACCTCTGCCTCC
CGGGTTCAAGCAATTCTCCTGCCTCAGCCTCCCAAGTAGCTGAGAATACAG
GCGCACACCATCGCGCCCAGTTAATTTTTTGTATTTTAGTAGAGACGGGGTT
TCACTGTGTTGCCCATGCTGGTCTCCAACTCCTGAGCTCAGGCAATCCACC
TGCCTCGGCCTCCCAAAGTGTTGGGATTACAGGCATGAGCCATTGCACCAG
TCCTAACCTATCTCTTTTGACTCAATCTAAAAGTTTCTGTCTTTTAATACAAAA
CCACAATCCATATGCATTCATTAATTCACAACTGACATTTAGTATCTTATTTCT
GTTATCCTATTTCATATTTTATGATTCCTTGTTTCTGCTCTTTTGATATATAAAT
TATGTTTTATTTGCCCTTATCCTTTCATGTGTTTCTAAAGTATATAGCCTACGT
GTAATTGTCCCATTAGCTAACTTTATGTTTTTGAAAGCATTCTCTCTCAGAAT
TCCCATTTTAGTGGTGCAGCACACATAGAAAGTCTAAGTGCTTTCTGGAGCT
AGATAAGCTGGATAAAGGTGTGCATGAGCCACTGGTCAATGGCTTGTGCAG
GCGGTGAGTGCATTTCTGGTATTTCATATGCTATTGATCTGGCAGCCAGGTA
TTCAGATAGGGTATAACCAGGTTCATCAGGCTCAAAACATAATCAAGTATTA
TTGAGACATAGTTAATGTGCACTACAACTCACAGCACACAGGCTCACACACA
CACTTGTCTGAAATAAAATTCCACAAAATAATACCTTCCCTTATTCTGTGTGA
TGTACTTTGATATATTCTCTCCTGTTTTATACAACTTAATTTTTTTTAGAGAAA
AGATTTTGCTCTGTGGCCTAAGCTGGACTGCAACGGCACAGTCATAGCTTA
CTTCAGTCTTGAACTGCTGGATTCAAGTGATTCTCCAGCTTCTGCCTCTCAA
GTAGCTGAGACTTCAGGTGTGCTCAACCACACCTGACTAATTTTTTGGTTAT
TTAATTTGTAAATATGGGGTCTTGCTATGTTGCCCAGGCTGGTCTCGAGCTC
CTGGCCTCAAGCGATCCTCCTGCCTTGGCCTCCCAAAGCACTGGGGTTACA
GGCATGAGCCACCACACCTAGAATACAACTTAATTTTTTAGTGCCAGTGACA
ACCCACTGGACTGATTTCATAACCCATTAGTAGAGGAATGCACCATCTTGAC
TGAAGGTTGGAATTTTCTCAGGGAATCTATGTAGCACTGATGATTGGGTTTC
ATATCCAGAGATTCTAGTTATGCTAATACAGAGGCCAAGCAAACTATAGCCT
GTGAATGGCCGGCCCCCTGGTTTTGTATACCTTACAAGTTACAAATGATTTT
TACTTTTTTAAGTGCTTAAAAAAACCAAAATAGGCCGGGTGCAGTGGTTCAA
GCCTGTAATCCCATCACTTTGGGAGGCTGAGGCAGGCGGATCACGAGGTC
AGAGGATCGAGATCGTCCTGGCTAACACAGTGAAACCCCATCTCTCCTAAA
AATACAAAAAATTAGCCAGGCTTGGTGGTGGGCGCCTGTAGTCCTAGCTAC
TTGGGAGGCTGAGGCAGGAGAATGGAGTGAACCCGGGAGGCAGAGCTTGC
AGTGAGCCAAGATCATGCCACTTCACTCTAGCCTGGGCAACAGAGCAAGCC
TCTGTCTCAAAGAAAAAAAAAAAAGAAAGACACAAAAAAAAATCAAAATAATAAT
AATAATATGTGAATATTATATGAAATTCAAATTCTACTGCCCACAAATCATTAT
TGGAACATAGTCATACTCATTTATTTATGCTTTGGTTTACATATTGTCTGTAG
CTGCTTTTGCACAGTGACAGAGTTGAATATTTGTAATAGATGGTCCACAAAG
CCTAAAGTAGTTGTGGCCCACAAATCCTAAAGTAGTTACTCTCTCTCCCTTTA
CATAGGAAGTTTACTAATACTTGTGCTAAGGGATCTCAACAGACAATCTGAA
AAACTTAAGTTTTAGACTAAAGATTTCCAATCTAAATTCCTGTGGAGCTTTCT

GAAGCTGCCAGGTGGAGATGGGAACAGGTTGTGAGGCTGCAGGCCAAACA
CTCAGGCCAGCTTCCACCAAGCAGTTCAACTCTGTCTGTTTCACACACTGAT
GAGCTTATCCTTGGAAAGTGATTAAAGTAAAATTAAATGCGAATTGAGGGAG
GAAGTGAGGGAGACTGTGGCTCTAAAACAAAACCCTAAGAAACACCAACAT
TTAAGATGGCAAATGATGTTATTTCTAAAGTCGTTCAGGCTAATATCACATAC
TATAGCTGTTCACTTTATAGATAAAGGTGACACTACAACCATAGAAAATGTAA
GAGTGGACCTCGAAACTCAGGAAGATGAAGTTTACATATATTAATCTATATTA
CCAACTGGAGCAGTTGTTCTCACTGCTGGCCGCACATCAGAATCCAATTCCT
GGGATATCACAGATGATTCTACCATGCAGTCAAGGATGAGAACAAACTAGG
TTCATTTCTGCAATTTTTTTATTGTTCAACCAGTGAAAAGGAAGTACCAGTGG
TGTGAGAACTTTGGGATAAAGTTTTTGTTTTCAATTAAAATTATTTTCATCCAG
CCCAACTTCCTTAAGCCCAAATTTAATGTGTGTGAAGTTCAGCTACAGAAAT
ACCAAACCTTAGACTAAAGCGGACACAGGTAAAATATGTGAAATCCTCTTTT
GTTCTGAGGATTCTTTAGTAGGCAGGAGTGACCAGATAGGAATATGCTTGG
CTGGAAAAATTAAGATTCAAGTTAACAAACTGTTAATAACCAGGACCATCTG
CTCTTCCGTAATGTGGATTTGCCACTGCAGGTCACCCTACAATGCTATGTTA
GAGGTACAACACTCTTACCCTCAGGCTATAAACAAGGTGAATTATTATCTTTA
TATCTCTTCATTTAGCCCTGATTTGCTGAAGTGAAGGCTCGCTTGAGAGTTG
GTTGCATTATAATTTGGTGAGAATTTAATCTCTCAATGACAACTTACTTGATT
CCCTCATTCTCTTTCTGCTACATAGATCACAGTAGACCTTGGCAGACAGTTC
TGTAGTTACATAGGTCTGAATTCAAAATCCAGGTCTGCCACTTGGTGGCTGT
GTGAACTTAAGCAAGTCAGGCAATGCTTCTGATGTTTTTTTCCTCCTCCACA
AAGAATAATTAACATATAACAATAGGGTCTCAGCTAGTTGTTTTAAAAATGGT
TAGAGAGATGTGTGGAATGAAGTAAGTGTGCAGTAAGTGTTAACTACAAATA
TTATTATCTTAGACATACAGATTTCCATGATTCATGAATGGTGAAGCATCTTA
GAAGACATCCATTCCAGGCCAGGCATGGTGGTGTGCACCTATAGTCCAAGT
TGCTCAGTAGAATGAGGCAGGAGAATTGCTTGAGCCTAGGAGTTTGAGGCT
AGTATGGGCAATATGGTGAAACCCTATCTCAAGAAAAAAGCAAAACATTTTT
TAAAGTTTAAAAAGAGAGACATCTGTTCCACTACTCTCATCTTAGAGGCCAT
AAAACTGAGGCTCAGATAATTTCAGAGACTTGCACAGATCCCCCAACCATTT
GGTGGCAAAGCCAGGAAGAGAACTCTGCTCTCCTTTCCCACTGGGACAGTG
GAAGAAATTCGTCTTGATTTCCATCTGTCCAGGCTGAAGAATGTGCACTGGC
TGGAATGACAGACTGACCGACTTTTTTTCTCCACCTCTGCTGTCTCAGCAAT
GGTTTGGGACAGTGTGGATGACCAGAAGCTGGATAGTACAGAGCCAGGCT
AAAGAGTTCAGGCTTCCTGAAGGGAAGCTGCAGTCCTCCTAGGCCACAACA
CCTTCGAGATAGAATACATAAAGCACCCTTCTCTACCAAGTTAGGAAAGGAA
GAAGTGTGACCAATTAGCTGTATGGGGACTGCCAAAGCATGCCAGTCTGAA
GATGAGCAGAAACTGGCTCATTCCATTTGGCACCTAGCACACTAACTGCATC
CGTTAATAGGCCATGCTTTTCTCCAGAGCCATTGGCTGAAGAGATCAAATAA
AAAGTATTGAGAATAGGCTACCCAAAACAGTAGGCTCAGATGCTATCACACA
AAGCACTTTATCCTTAAGTTCAATTTTTCTAAATTGTAGTTGGCTGCTTTGGC
TTAATAAAAACTTCCAAAAAAGAAAAACGAATGGCCACAGACAGTATGGGTA
TCTAACTATATTATCACAACTTGACCAAGATTGAACTTGCCAATCCTTTGGTT
CAAGAGCCAAACAAAATCGTTCCCTTAAAATATTGCTTCATGGGAACAGTCT
TCTTCAAACATCTTTTAGCACAGGCAAGATTCCCATTTATACATTAATTCTGT
TCAAGACAATGAGATTGGGCAGAAAAGGCATTGAGTTGGAAGTCAATGGAT

ATGAGTTTTTATCCCAGTTTTACCACAAATTAGCTGAGCATAACTTCCACAGA
TGCATTTATCAAGTAGTTTTCATGGTCATTGCAATGCCAAAAAACTGTAGCAT
TTAGAAAATTTAGTTTTCAGACTTGGAAACTATTTAAGGCATTTCATATGAAG
GGTGTGTCCTTGTGAGAGTTTGCTTATGCAAGATAAGGCTTCTTTCAGCTGC
AAGTCAGGAGCGAACCAAAACTCAAAGCAGCAGCTGCATGAGCTGACTTTA
TCACATCTTGACAAGAGCTCAGCCACTGGAAGTTTTGGCATACAGCGAAACT
GAAGCGTACTTATACAATATCACATTTTATTTTTATTGTTTCTAATAGCATTCC
AGGTTAGAAATGTCAATTATTTGGGAAAGCTGAGGGTCTGGTAGATAAAGCA
TGCAGCAGAGAGCTAGGAGGCTGGCTATTTCCAGTCGTTATCCTAACATGT
CTTGGGCCCCCAAGTCACCCCACCTCCATGGTACAATGGGAACTGTGGCAG
AAGTCCACGCTCTCTCCCCCAACACATGGGGATAAGAGACAAGAGAGGTGA
AATGTTCTGGAACATATCCGATGTTATACAAGTATAAGCTGTGAGATGATCC
AAACGCAAATATTGAATATTTCATTTTCTAGAAAGTATACCAATTCATTCCAC
CCTTCTCAAACCTAAATTACAGAATTCAATTCAGGTCACACAGATTTACTTTG
TACTAAGTACCATAGCAAATGCCATTTCAGTGCCTGAAAACTGAAAAACATA
AATTTAAAGTAGGAGTTTGAGGCCTCACTAATATGACAAAACATACCTTTATA
TTTTATTTTGCAGTAATTTGCCACTTAATCATTAAACTCTTATCAATCTGAGAG
ATTTGCCAACACTTGCCTGCTAGGTGACCTAAGCCTCCACATCAATGCATGT
TATACTCCCCTTTCTCCATATGTTAGGCCCATGCTATTTCTTTATCCCTCCTC
CTCTGCATCTTCACCTAAAACTCTGCCCATCCTTCAGGGTTCATCCAGTGAT
TCATTTGCAAGCAGGCATGGGGTAAGGTCTTCAGAGTATGTTTCTCAGAGG
CCCATGCAGCTAAGAAAATGTGCAGTGTTGGCACAAGGTCTGTCTATTCCT
GGGTAGCCAGATGCTGGACACATCTTTCATAACACCACAAGGTAAATATACT
TCACTTGGAGAGAGAGGTGAAATTTTGCAGGTATAGACTGGATGTGTTCCT
GCCAGAAGATGTGAAGGGATTAAGAAACTGACTCTCATCTCCGTATTGCTAG
AGCAAAACATAATTTCTCATAGTGGCTATAGTATAAGGACACTGAGGGGTAA
GAGATATAATCTAAGTAATACAATAAATTAGTGTGGAAAAATCATCAAAATGA
AGACTACATGGTTTTTACTAAAATTCTAGCTTTTAGGATGTCCAGGGAGCTC
AGGAATTTAGCTGTCCTTTTTTGTATGTACAATATGCCCCAATGCTTGCTGAC
TAATGTACTAAAACATTAGAGAAATCTTGCTGACAAGATCTCAACCAGTCAG
CGAGATCCGGAAGGTGAGACTAATATTGAGGGTCAGCAGAATTAAGTCTCA
GTTCTGCTGCTTACCAGATATGCTGATCTGAGCTAGTCATTTAATTTTTATGA
GACCAAATGTCTATCTGTAAAGTCGGCAATTTGGATTAGATGTGCTGCAAGT
GGTTTTCTAGCTTAAATGTACCTTCTGAATTCAACAGGACAATACTTAAACTG
ACCTTTAATCTAGGAATGACACAAGTAGATTTTTGAAAGCTACTTTAGCTACA
GAAAGCTGAGAGCACCAAAGGCAAAGAGATAAAAATAACAGGAGAGCCTTC
CCTTAATCCAGTCCCTAAGCAGTTTTGGCAAACTAAAGTTTGTTGTTCAATG
GTTACGAGTTTGCTTCAATGCTTTCTACCCAGTTTACTGAACTAAATAGTATA
TAGCTATAGTAAAAAGTCCTATTCAAAAACCAGCTTCTCACAGATATTTTGCA
GCTTTGCAGAATTGAATATGTCCACAGACGTCTATTAGCTGGTTAGGGTCTT
AGGAATCTAGGAGAGCCAAGTAGTTGTGTGAGCTGTTGTTATCAAATGTAGT
TTTGAACATTCTTGGTGATTTTAAGGGATCATATTGTGGAAATTTGGTTTCCT
TACCTTGAATTTTGAATGAAGCTTTAGAATTTGAGGATGTTTCTTTGGTTTCT
CCTTCCAGGTAAGTGATTTTTTTTTTTTTCAACCAGATGCTGGTTTATTTAATT
TGAAGGTATTGATGAAATTCTTTAAATTGCCCCCATGTGATTCTACTCTGGAA
TAACTACGAAATTATTTAAAAGTTAATTAATACAAGAAAATATGAAAACTCATT

TTTATGGGAGCTATTGTTCCTTCAAGATGACACTGTTTTGTAAACTATAGACT
TCCAGTAACAAGCCTCTGTGCCTTCTTCTTACCACTAAGCATGCATGGGTAT
TAATTCCTACTGAAAGACTTATGCTATCTTTTTTCCAGAAATGGAAGAAAAAT
GAACTATGAAAAAGGTCATTTTATAGGTCAGCTACCACTATGAGATTGTTGA
GGAAATGATATAAAAAACAATTTTTATCAAATTATCTTTAGGGCATTTATATGT
TTATTTTCTTACTATGTTGACTTAGGTGACTATAAGAAGTTGTATCAGAGCAA
CTGATTCTGGTGAATTAAAGCAAGTATTTCTAAGAACATAAGTGGCAACTTTC
AGTCTCAAATCAATTTGGCCACCAATCAGTTTTTGTAAGGGTACAAATAGGA
CATAACATGCTCAGATGGGACTTGGATAAAGTGTATACAATTTTACATCGAG
GAAATTGTGTCAATGTGTTACCTTCAATGTTAGAAATTCCCAAGTTCTGACAA
TAGTTCAGAGCCTTGTTAAAAGCCAGAGTGGAGGCATGTAGATCCAGCTGG
AAAGAGAGGCATTATGGTCTAACTTAGGACAAATTTTAAAGCCAGTGTTAGG
GTCTGAGTCCAGCTTTGTAAACTTGAGTACAGTGTTTGATCTCTGGGGTTTC
AGCCTTCACTTCAGAACAAAATTTCCACCAAGTGCTCTTTTACTGTGAGGAG
TAGCTGTTGAAGAAGAAAGAAGTCTACTTATTTGCTAGAGTGTTACAATTGTT
TTGATAAAGCTCAAAACTTATCTAAATAAGCTCTCTCTCCCTAAGCATGTTTT
CATTTTTATAAAAAAGTTACATATACTTTGCTTATAAATTTAAAATACTTTTCAC
CTCCTCTGACTTCATTTAAAATTAAAATAATTAAAGTGCCAATTTTAAGAGAT
GTTAGCTCCCATTATTGGTTCTTTGCCATATTCTTTTGACAACCTGCTGTAAT
TTTCTGCCCCCTTTAAAGCCTCAGGCTATAGGCCTTCTCCACCAAAGGAATA
TTAAGAAGTGATAAGGACCTTCTGTGAGCAGAAGTGGCTTGTTTGCAAAGG
GACTGCTTATCTTGGCCACTCTTGAACACAAGATGGGACCCTCTACTGCAAA
GCTCTGGCATGTTTTTTTTTCCCCTAAGTTATCCTCCATACTACTGACAGTGA
TTTTCCCTAAATAAAAAACTGCTTCAAACCATTCATTGTCTTTCCACTGCCTT
AAAGATAAAGTCCAAATTCTAGAACATGGCCCACAGCATTTGGTGCCTCACC
ACCTCTTCAGCCTCTCAGTTGCTGTTCACCCATTTCTCTATTCCTCTCCTTCT
CACACCTTGTGCTGCAGCCACATAGATAACCTGCAGTTTTTGTAACGTGCAA
TGATGTCTCAAATTCCAAGGCATTGCTGGTACCACACAGCCTGCCTGGTAAA
ATCCTAGACTTCTTTCAAGATAAATTCAAAGACACCTCCATGAGGTCTTTCTA
CCTCTCCAAGTAGAGTTGACCGCTGTCTCCTTTGTGTCCCCACTTCCACCAC
CATCCTAAAATACTTATTATACTTAGATTAATAATTGTCGCTCTTACTGCACTG
GAATTACCCTGAAAGGAAAGGCCATGTATTATTTATCATTGTCTTCCTAGTAC
ATAGCCCACAGCCTATACCTCCCACCCCAAAAAAAACCTTTTGTAAATAATT
GAACAAATTAAGAAACACCCAAGGCCCCCAGTAAACATCAAGGCCTAAGGA
ATGCATATCTGGATTCTAAATAATCATAAGGTTTTACAACACCATGTTAAGCA
CCAGGGACTTCAGAGAGCTTTTAGTCTAAATCTTATTAGAGAGGCCAGCGAA
GACCTCCCAAAGGAAGTGGCATTGAACTGAGACTTGAAAAGCCAGTAGTTA
GGCAAAGATAGGGAGGGAAATATTTCAGACGAAGGGAGGAGATGGCACAA
GATTTAGGACACGGAAAAGGGTATGGTGCAGTCATAGAGAAAACAGATGTG
CAGAATGGCTGGAGCCCCAAGAGGGAAGGGAAGGGCGAAGCAATGAAGAT
GTGAGGCAAGCAGGACTGGACCATGCAGAGTCTTGCAGATGTTCACAAAGA
AAATTGCAGCAGGTAGTCCCTAACATCGTGCTGAACAGTTAGGCAACTTGG
AGGAATATGTATATTTGTACTCATAGTCAAAACCACTAGATGGCATTTACAGA
CTACGTTTTGTGTATTTTTATTTTTTACTTTTTGTTTTTTTTTTCTTATGTTAGC
AAAAGTATGCTCGCTATTGAAATGTTGAAAATATTTCATTGGTCTTAAAATGA
TGCTTATTTTTCCAGATGCTTGCATTCATTCTGCATGTGCTATTTTGTCATGT

GGTTTGCTTAATTTATTAAACAATTGTATTAATTAAATATATTAATTATAAATTG
ATTAATTTATAATTAATTATGTGTTATAATTAAGTTAAATTTATTAATTACTTAA
ATTATTATATTCACATTCAGATGCAATCTGAAAACCCATTTGTTCTCACACTG
CTATAAAGAAATAACTGATACTGGGTAATTTATAAAGAAAAGAGGTTCCATTT
GACCCAGCCATCCCATTACTGGGTATATACCCAAAGGACTATAAATCATGCT
GCTATAAAGACACATGGACGTGTATGTTTATTGCGGCACTATTCATAATATC
AAAGACTTGGAACCAATCCAAATGTCCAACAATGATAGACTGGATTAAGAAA
ATGTGGCAAATATACACCATGGAATACTATGCAGCCATAAAAAATGATGAGT
TCATGTCCTTTGTAGGAACAGGGATGAAATTGGAAATCATCATTCTCAGTAA
ACTGTCGCAAGAACAAAAAACCAAACACCGCATATTCTCACTCATAGGTGGG
AATTGAACAGTGAGAACACATGGACACAGGAAGGGGAACATCACACTCTGG
AGACTGTTGTGGGGTGGGGGGAGGGGGGAGGGATAGCATTAGGAGATATA
CCTAATGCTAAATGACGAGTTAATGGGTGCAGCACACCAGCATGGCACATG
TATACATATGTAACTAACCTGCACATTGTGCACAGGTACCCAAAAACTTAAA
GTATAATAATAATAAAATAAAATAAAATAAAATAAAATAAAATAAAATAAAATA
AAATAAAATAAAAGAGGTTTAATTGCCTCATGGTTCTGCAGGCTATACAAGA
AGCATAGTGCTTCTGCTTCTGGGGAGGCCTCAGGAAACAATCATGGCAAAA
GACGAAGGGAAAGTAGGCACGTCTTACATGGTTGGAACAAGAGCAAGAGA
GAGAGTGGGGAGAGAGAGCCTTGGAGCAGGAGCAAGAGAGAGTGGGGAG
GTGCCACACACTTTTAAACAACCAGATCTTATGAGAAATCACTATCTCCCAG
ACAGCATCAAGGGGGATGATGTTAAGCAATGAGAAACCAGCCCCATGATTC
AATTACCACCCACCAGTCCCCACTTCCAACATTGGGGATTACATTTCCCCAT
GAGATTTGGATGATGCCACAGATCCAAACCATACCACTCACCTAATTCTTTC
TACGTAAGAATTTGTCCAAGCATTTATAACAATTAGCATTTCATTTAACATCTT
TTATGAATAAAGCACTATTCTCATGCTGAGAAGATTCAAAATAATGGGAAATT
GAAGTCCTAGGAACAAGTTTTATGTTTCAGAAGAGCCCATTTGGTATCCACA
GGGCTAAGAAATGTGCACCCTAAATGTAAGTGGATTACACTGAACTGAAAG
GTGTAAAGAAGGAGTGGAAGATTAAAGGGAGAAGCTTGGAGAGGATGAAAG
TTAGAAATGGAAGTGACGAGCACACCTGAGTGAAGGATGAGAGCTCCAGCT
GCATTTTCCAGTTGTATTCCCATGTTGCTGAGCCAAAGGCTGATCTCAAGTT
TATTGTTACATGCCCATTTAAGGCTTCTGGCCATTAACACTTTTGATTTTTTTT
GGCTTGTTGTTTTACTAGCTATTTTCACAACACTTTCATAGCTAAACCTATTTT
ACTCAGATTGTATGCCTTTTCAAAAATACAATAGAAGGTCCATATTCCATTAT
CTAGAAATAAGCCAAAGCTCATATCTAACATTTATTAAGAGAGATGGATTATT
TTTGTTCATTAGTTATCTTTATAAATAATTTTTACGTACTTTAGTTGACTCATAA
AGATGTTTCTTTCTGTAATTTTAATCTTAATATTTGTTGAACTTCAAAATCCCT
ATCACCAGGTTATTGTTTAAAAGCATTGGTTTTTATATTATCTTAAAAGCCATT
ATACCTGAGTGCTGAACAACTTAGAAACATTCAGTAATTGTTTTGCATGCTAT
TTAGTGAATTCATATGGCAATCGTTTATACATACATGATGGAATCAGGTGGC
AGGCCAAGTTAAAGAGCAAGGCCAGAAAAGAACTTAAAAGAGAAGAGAAAA
AATAGACAGTTTAGGAACAATAGATCATGTCTTCTCCATGATTTGGAGGTAA
ACTGATTACCTATCAGCTGATAAATAGAGGAAGGTTTTAGAAGTCTTCAGTT
GGGTAGACTAATGAGAGGTGTCAGAGAAGATGTTTTCTGTTGTTTGTGGGTT
CTCCAGGAAACTTTGAGCATTCAGCTGAGGGGCCAAGTTGGCTGCCTCTGA
GAAGAAGCCCTTCCACCTCCACTCCATTGCACTTGGGTGCCATTCCCCTCA
GTTGAATATCTCCAAGAGATGAGCAAATGTACATCTACAGAGTTCAGGGTAC

TGACTTTTATCATAATGATTTATAACTCTCAGAAGAGTGAAAAACACATGAAT
GCACAGAATAGGAGATTGAAATATAAACCACAGAACATTCATACAATGGAAT
ACTCTGCAGTCATAAAAATCTTCTCATAGAAGAATATTTGACAGCATAGGGA
TATCTGTGGCATATTAAGTAGAAAGTCAGACTTGTAAACATTATATACATATT
CACGTATATTTAAACACCATGATCCCATATTTAGATATAACAACTAAAAGTTC
AGATGGCTATATATCAAAATGTGTCAAATGTTCAACCTTGCATAGGCTGACT
GTAGATGAATTTTATATTATTCTTTGTGCTTTCTTGTAGTTCCCAAATTTTCTT
TACTGAATCTATATTACTTTTGCAATTTAAAGAATTTAATTTATAAAATTTTATA
AAATAACTTATAAATTTGAAATGTATTGCATTTAAGAATAAAAAGTGTTTAATT
ACAAAAATAATTCACAATTTATTTAATGAGATTTTAAAAGGATATATGTGAGTC
TACATTCTGATTTCATGTTTGCATGCATGGTTTTTTTTTTCTTTTGAGACAGAG
TCTCGCTCTGTCGCCCAGGCTGGAGTGCAGTGGCGTGATCTCGGCTCACT
GCAAGCTTTGCCTCCTGGGTTCACACAATGTAATAGTGTTTTATTATTGTTTC
CATTTTTATTGAAGAAGTAAGATTGTCCCTAGCAGATGGAGACACTGAGATA
TGGGACAGAAGTTTTGTTCTATATAATTATTATGCGCTTCCACCTTTCTTAGC
ATAGACAGTTTCCAAAATGCAACTTCAAGTTACCCCTTTATAAGCATAATAAC
AATAATACCCAACATATATGTAATGCTCTTTATGTGCCAAGTACTATACTAAC
ACATGCACATTACATACACACACACCACATACACACACATATTTAAACTAATT
TCGTTCTCACAATGACATTTTGAGGCAAGTATTATTATTGTACAGATGAGAAA
ACCAAGGCACGCTTTATCTGTAAACCTCTGCTATGCAGAAATTCTGGAGGG
GCTTCTGGCCCCTTAATTTTAAAATAAGGCCAATAATACAATACTTACCACAT
AGCAATTCTCTAAACATTATGTAAGATATATACCAAAGCGCTTAGCTCAGGG
ACTGGAGGGATGTGAGGGAATTTGTCTTTTGCAATATGCTTTATGGTCCGCT
CAGTCACCTCGTTCTTAATCCCTTTCTCAACTTCTATTTTATACAGAAATTGT
GAGCATATCAGCATTAAGTACCACTGAGGTGGCAATGCACACTTCAACTTCT
TCTTCAGTCACAAAGAGTTACATCTCATCACAGACAAATGGTTTGTTTTCATT
TTTATTTTTAAATTGTGGCTCCGAAATCATTTTTGTGATGTAACCCATTTTAGG
GGACCTGTCACTGCAGAGAAACTGACAAACACTGAGAAATGCGAGCTAAGT
AGACACAGCCTACTAAGTAGACACAATTCCTACTATGGAGGAATTCTTGCCT
CTGAAATATCTCACAGAAATAATACTGTGAGTTAAAGAAATTAAAACAATGTG
GCAAAGCACAGAAATGATGCACGTGACCATGAAATAGTGGGCCAGATAAAG
GGGACCTAATAGTGCGGTGGTGCGGAGGGTCTGTGGGCAAACTGAGTTCA
GCTCAGACCCGGGCTCAGCTCTATGCCAGCTGCTGACCCAGGGTGAGTTG
CCCTGCAGGGTTTCTATCCCATTAATTTTAAAATGGGGCCAATAACACAGTA
CTTATCTCACAGCATTTCTCTAAAGGCTAAATAAGAAGATGTATCTAAAAGTT
ATTAGCTCAGAGCCTCACACATTCTCAGTGACTGATAAACAATAAGCAAAGC
TGGGTGCTGAGATAAGAGTAATCTGGTGGCAGTCTCTCTTGTTAGTTTTCAG
GGGAGAAGAAGAAATTCTGGAGCCGCTGCTGGGAGGGATGTGGGAGAGTT
TGTCTTTCATAATACGCTCTATGTCCACGCAGTCACCTCATTCTTGTGCCCTT
TCTCAACTTCTCTTATATGCAGATACGCACAAACGGGACACATATGCAGCCA
CTCCTAGAGCTCATGAAGTTTCAGAAATTTCTGTTAGAACTGTTTACCCTCCA
GAAGAGGAAACCGGTATGTTCTTAGTTTTAAATAGTTGCTCTGGAGTCATTG
TTGTGATTGAACTCTATTTACACGAGCTGTAACTCATGACAGTTCTCAAGCTT
TCGTGACAGAAAACCCATCTCTTTTACTCCAAAGCCCATATAGCACCCACAA
CTATTAACTGTGACCAAGAAAGAGAAGGCAAGCCCCAATTAACCTTTGTACG
TAAAGCCTAAAGAATGAAAAAATATACCTGAATCCTCAATCATCAAACAGCAT

AGTATATACTAAGTAATTTGTAATAATTAAACTCTAGAAAATTGTGTGGCTTC
GGTAGTAAGAGAGCTTCATGATGTAAAATGGCAAGTGGAGACAGAGACAAA
AGTAGGATGTGGACTGAGAGGGAAGGTTAGCACAGGTGGAACAGTAAGGC
AACCATACTATCAATTGCTGCTGACATAGAATCCAGAGAGACTATTGGCAAA
AGCTCAAATGAGACACAGTAACAGTTTAGATTCAGACAGTGGCTGTGGCATA
AATCAGAAAATTGATAGTCGCATGATCCCTCTTTGCATGGGACTGGCATCTG
TGTGGAGTAATGGTTCCATATGCCTCCTTTCTTCTCCTTATTTTTAAATTTTTT
AAAAATGCATTGCTTCTTGTGGAAGTCAATAAGTGATTCTTCCAATACTTTCT
CATTCCTTCCCCCTCAGTTATGAGACAATTTGCTTATTTCTCATCCATGAATA
CTTGTTGGGTCATTAAAAGTAGATACTGAAATTACTAATGGTACGACTGACA
TATTACCTCATAAATGTTACTAGCTAGATGTTGAAAGTTGACCAACAACTCTC
AAAATATGATTAAGAAAAGGAAACCCACAGAACAGTTTGATTCCAAAATGATT
TTTTTCTTTGCACATGCCTTACTTATTTGGACTTACATTGAAATTTTGCTTTAT
AGGAGAAAGGGTACAACTTGCCCATCATTTCTCTGAACCAGGTATGTTAATA
TTTGACAAAGAATAAAAGTCATTCCATTTTAAACTATCCATTGCTTGTTTCAAA
TGCCTAAGAAAATGTGTCTATCTTAGAAGAGCATATGTTGTTAACTTTATTCA
CACAAAATTGTAAAGGCAAAGAAAATATTCTCTTTTTAAAATTAAAATAGGCA
TTTCTTATTTTTAAAAACATTTTGGGGGCCAGGGGCCGTGGCTCATGCCTAT
AATCCCAGAACTTTGGGAGGCTGAGCCTGGCTAATCGCTTGAGCCCAGGAA
TTTGAGAACAGCCTGGGCAATATGGCGAAATCCATCTCTACAAAAAATACAA
AAATTAGCTGGCATGGGGCACGCACCTGTAGTCTCAGCTACTTGGGAGGCT
GGCTGAGGTGGGAGGATCGGATCCATTGCCTGAGTCTGGGAGTTTAAGGC
TGCAGTGAGCTATGACTGTGCCACTGTACTCTAGCCTTGGTAAGACCCTGT
CTCAAAAACAAATACATAAGTAAATAAAAATAAATAAAAACATTTTGGAAATA
GAAATACATAATTTGGTAATAGTTTTTCTCTTAAGTTAGATGTTTTACCTTTCT
AACCAAGCCTGAGTACTTGAAAAAAGCCTCATAAGAGCTTATAAAACAAATG
AACTTCCCTCATATAAAAAGCAAGGCATTTAAAATCATCTAATTAACTGGTAC
TGTATTTCAAGGGTAAATCTCAGCCTTGATTCATTTTTGGCCCAATGCAACCA
CTTAGGGACCATCTTGACAACCTCTGCTGAAGGGACATCCCTTCCCCTCACT
TGAGTATCACTGTGTGTGCTCATTTGCTATTCTGCATTCCAACCCTCCCTTCA
CACTTGGCTGTGTCCACGGCTCACAGGGTAAAAAGCACATCATAGAACTTC
ATCACTATCGCATACATTCAAGCTAAGTGGTCAAGAAGGCTGGGCAACACC
AGCAAGAGGAAATGCTACTTTTACTTTTTATCAACAATAGGGCTTTTAAATAT
TAATTAGGCAAATAAATGAGCCATTTTACCTTTATGTCTAGCCTTCCATTCTA
TTTACTTCAACTGGAAGCACTACAAATATGCTATAAATATGGAAATATCTCTT
AATTGATTTCAATTGTTTCATTCCCAACATATAAATGACTCAACAAGCATTTTT
AGTGACTACATTGGAGACTATGCATAAGAATACTATGGAAGGAATAAAGCTT
AGAACATAGATGACCTGCATTATAATTATAATTCTACTTTTAACTAGTTGTCT
GACCAAGGCTAAGTTAACCTTATTCAGCTTCTTTTCTTCATTTGTAAACTGTT
TATACCAGTTTCTTTCCAAAATTATGATTCTATGATCTGTTCAATGCTCTTTTA
TACATTAAGACATTATTTTCTCTCATAACTTCCAAACTATGGGAGAATTTGTG
GTTTTTTCCCCATATCTGAGGAGAACGTCCACTGAGTTCTTATCTACAGTTAC
ACTAGTGAAGAACGCTGGGTCTGGAATCAGAAGCTTCAGGTCTTAGTTCTG
TCATCAACTATTTTGCGACCTTGGACAAAAGACTTGATCACTCACAGTCCCA
GTTTCCCACAAGGTTACTGTAAAGCACACAATTTAAAAAAAGACAAAATCTAC
ATAATAGTATATTAATTGTGCTTTCTATTAAAAGGCAAGGTGATGGTATGCTG

ATGTTATCTGTCTTATTTTTCAGTTGCTATATGGTCATTTATTTCAGACTTTCA
TAATTTTGCTGCTCTCTTTATCTCCTGTAGAGATAACACTCATTATTTTTGGG
GTGATGGCTGGTGTTATTGGAACGATCCTCTTAATTTCTTACGGTATTCGCC
GACTGATAAAGGTGAGAATTCAGTTTTTAATTTTGCTGTAAATACCAATGTGA
ACAGCTCTAAGAGGGTTTATTCCTCTGAGTTCAGTTAAACTCAAAAGAGAAA
CAGAACTGCATAAAATTCCATATTTTTCAACTGGACACATAGAAGTCACTGT
GTTTCTCTAGCAGAATTTTTCTTTGCATTTGCCCAATTAAAGGGAACCTCTAA
ATATAAATCTGTCCCCCATTTTCCCAATGAAAGATCTCCCTAAGTTTTTGTCT
AACTTGCTGTCACATATTTTGATGGATATTGAGGAAATATTAAGATTCTACTT
ATAGTATTTACCCTATTAGTGTATAAAATATTTAAAATAATATATTTACATATG
TTTAAAACTTTGAGGGAAGCCAAGGCAGGAGGATTGCTTGAGCTCAGGAGT
TTGAGACCAGCCTGAGCAAAAAGGTGAAACCTAGTCTATACAAAAAATATGA
AAATTAGAAAGGCGTGGTGGTGCACATGTGTAGTATCAGCTACTCAGGGGG
CTGAAGTGGGAGGATTGCTTGAGCCTGGGAAATCAAGGCTGCAGTGAGCT
GTGATCATGCTACTGCACTCCAGCCTGGGCAACAGAGTGAGACCCTGTCTC
AATAATTATATAAATAAATAAATAAAAATAAACAAAATAAAACTTTTGCCTTTC
TTAATTCTCACATATTCTGAAACAGATTTTTCAAATTTCCACCCATGAATTCTT
AACATCAGTGATTTTTTTTGAATCATTAATGCTTTTTTTAATTTTTTTTTTTTTTT
TTGAGACAAGAGTTTCCCTCTGTCACCCAGGCTCGAGTGCAAAGTGGTGCA
ATCTCTGCTCACTGCAGCCTCTGCCTCCCTGGTTTAAGTGATTCTCGTGCTT
CAGCCTCCGCAGTAGTTGGGACTACAGGTGCGGGACACCATGCCTGACTA
ATTTTTGTATTTTTTTAATAGCAGAGATGGGGTTTCGCTGTGTTGGCCAGGC
TGGTTTCAAACTCCTGACCTCAAGTGATCCATCTGCCCTTGGCCTCCAAAGT
GCTGGGATTACAAGCATGAGCCACCACGCCCAGCCCACTAATGCTATTTTT
ACATCCATACAACACAGCTTATCGAAGTGCATAACTTTTGCTATCACTTTCTA
TTCACGATATTTAAGACATAATATGTGTGTGTGTATTTATGATGCTGTCACTG
TCTCTGTAATCCTAGATCAGAAGTACTTAGTCACATGAGATTGGTACAGTTG
TGTTTTCATTCATCCTCTATTCTTAATCTCTCTTTGTGATTTTTGAGACCATAA
CCACTATATAATTCTTTTAAAAAGGCTGAGAGGTGTGACAGCACTGCAATTG
TGGGGCCATCAGAAGATATGATAGTAATATCTACATTAAGTTCCTTTGCCTCT
TTTCTTTTTTAACTACTTCTAACAGTTAACTTCTACCATCATCCAATCCTATAA
TTGATTTTCAGTATTCCATGTAAATATATCTTCCTTAAATAATACTTTTTGTTAA
TCAAAGAAAAGTAACTGAAAATGCCTACTCTTGTGTGAGATATTTTGTAAGG
ACTTTAATATAAGATAGCTTTTTTTGCCTGGAGTATAAAAGAGAAAAGTCATC
TTCTTACATGGGCATATATGGCAAAGTGGGTTGTCTTCTCTCTTCGTCAATG
TTCTAAAACCTGAAAAAGCCAAGGAAATATTTAGTTGGCAAAGTTCAGAGAA
TTTTCTAAGTGTATATGGATGAATTTTGTCCTGGTCAACATGATGCAGAGATC
ACACACTTTATTTTTATTTTTATTTTCACTTTCACTATTTATTACAGCAGGGAA
ATATGTAAGTATCAGTGTTTGAGGTGATATTTCTCCTACTGAAATACCAAATA
CTATAGAGGAACACAAATACAAGTTTAAATCAATGCTTATACCAGTAACTAGT
AACAACAACAATAACAAAATCTCTGCAAAGGGGATTTCAACCAAAAGAAAAA
AAATTTTAGAAAAAAATATTTTTAAGCTGAAGCATTTTACTTTTTACTGTCTTA
AGACTAGAAAATTGTGTTATTAATATTTTATGGTATTTCTTCATAGAAAAGCC
CATCTGATGTAAAACCTCTCCCCTCACCTGACACAGACGTGCCTTTAAGTTC
TGTTGAAATAGAAAATCCAGGTTGGTGTTAATATTTGCAGTTCCTTTTGCCTT
TTAGGAAAAAAAAATCAAACCAGTGAGTTACTTCTTTCTGATTTGAGGGAGG

AGGGAACCAGTTATGATTCATTTCTATTCTATCTCATTAATTCTACTTCTTTGA
CTTTTTAGAAATGTCTGCAGCATAGTGAGATTCTCCTTTGGACACAAAGTGTT
TTGTTTTGTTTTGTTTTTTTAACAAAAAAAAAAAAAACTCAATCAAATAGTAAAA
GCAAAAGAGAAAACCAAGTGTACTTCGTATTTCCCAAACTGCAAAGTTATGT
GTATAGGAGACTCTATGGTCAGTATGGTGTAGCATAGTGAATTAGCCCCAG
ATCTGAAATCAGACTTGGATTTGAATCCATGCTCCAACACCTATTAGCTGTG
TAACCCTGAGCAAGCTACTAAACCTCTTTTAATATGGGGATAATGATAGTAT
CAACCTCACAAAGTTTAATGAGAATTAAATGAGCTACAACCGGTAAAGCATT
TAAAACCATTTGTGGCCATCATAAGTCCTCATGCCTGTTAGCTGTTATCAATA
TAGCACTGACATCAATGCTATATCAATATAGCATGTTATCAATATAGTGTCAT
TCCCAAATGACCTCCTGTGCACACTGGCAAGCCATCTGGCACATGCTTTCAT
CTCCACTCCCAGGTGCTAAGCAGATACAAAACATGTGAAAGGCCATGGATA
TATTTTGTTTATCCAGAACAGTATTAAACCACATAGTGCTTTTTGAAAAGAAT
ATTTATTGTCAACCTTTAAAAGTCGGAAATTGTTACATTTTAAAAATCAAGTAT
TGCTATTCCTCTGGGGAAAAATGTAAACTCCCAAAATGCTGAGAGCCTTCAT
ACCAGCATGAGACCAATTCCTAAGAGCTGAGTAGTGGCTGCTACCTGTACT
GTCTGTCTAAATCCCTAGCCAATTGCATTTGTTTTATTCACCGTGGCCCCTG
GTATGAACTCACTAAGAAAGCATATAGTTTCTATTAAACTTTGCCTGAAGCAT
AAACCCAAATGACATCTATTTTGGGAGATAGTTACTAAGAACAAGTCTCTGG
AATGAGCTTTATTTCTCAAGCAAAAGAGATTTCATTCTGCCTTCTACAAAATC
AACTGATTTTACTCCCATAATTTTCAGAAATCATGACAGATCAGAGGTCCTGT
ATGCTTCTGGATTTCGATTTTAACCCTGGGCCAGTCTAGGTTTTCTAGACTTT
AGAGTCACAGAACACAGAGTTTTCAAGATCCATCACAGCTACACAGGTTATA
TGCAGGATTTGCCACATCACATTATCATGTGAATTCTTAAAGCTTAAGAGTAA
TTGTTACATAAGTTTATAATCCTAAGACATTCCTGCTATGTGGAAATGAATGG
CATAGATATGATTCTCAGCTAAAAGGATTAATAAAATCCAATCTGCAGATACT
TGAAACAACGGAAGTTTTTGAGTCATATGCCAGATTCACTTCATTTACTAAG
GTTATCTTGTTATTGGACTGGCAGCTGGAACAAGTATCTGTAAAATATTCATT
TTATCTGCATTCTGCCTTGTTCCACAAAAAAGTCTTGATGTAGTTTTTCAAGT
GGAGCAATTACAACCTAAAGCCTATTTTTCGAACTGAAATTTATATACATTTT
TAGCTACTTATTTATTCTAGAGACAAATTTATTGTTTAGAGTTTCCCCTGCCA
TTTTTTTCATACAATTTTAAGCATCTCAAATGTTTGGCACAATTTAATACGCCA
CAGTGCATCAAGATGTCCTTGTAGTTTAATTCAGTTAAGTGCAACAAACATTT
GCTAAATGCATACAGTGGGGTAGGCACCACACTCACATTAGATATACCAATA
TGAGTCTTCGTCCTTTAGAAGCTGAGAGACTAATGGAAAAAACAGAATGTCA
TTGCAGTGAACAAGTTCTACAGTAGTGGAGGCAATAGCTCCACTTGTCCCA
GAGACTGAGACAGGTATCAAAGGCTTCTGAAGATGAAATCACCTGGGATTA
GCCTTAAAAGACAGATAGATATTAGCTAGGGCAGGGTAGTTTTAGCAGAAG
GGCAGCCTGAGTGAGTAAAAGCATGGAAGACAGAATATGTTTACTTAAAGAA
TTGTATGCATTTCCACATTAGCAGGATTGCTGCTTTGGTTCTCTGTTCACATC
TCAAATATGTGTAATGGCAGTGGAAAGTCAGAAGAACCAAACTTTAGGCTCA
CTTTATTTCCCCACATTTGTGCAAGTGAAGTTATTAAATGTCTTAGTATGTTA
GTGAGACAAGTTATGAATTCTGACTGCACCTCACAGAAAACATAGGAAAACA
CATTATTAAAGATTATTTAAAATGCTTTATTTCTACTTTTATAGAATATGGCTC
TAAATTAGTTTATAAGCCAAAGGCATAAGAGGTTAAAATGACAGTACCATCT
CAACAAGAACTAATGATGTAAAGGAGTAATTAGAGTATAAATTGTTTTAACCT

TCTAAAAGTGCACATGATCTGTGATTGGTGAAAAATGAGAATAAGCGAATCT
GAGTCAGCTGGCCACTGTGGCATGCATATGTGACCCACTAGCCTATTTCCC
ACAGGAGAATGTTTGAGATGCACAGTTCCTGTGGTGCCCAAATAGAAGAAG
GCTGGAAAAGCTCTGCTTCTGGAAGAGCAAGGGCTCCCCTCTCCCTTTCAT
GCAGTTTCTAGGAGCAACATAAATTCAACCTTCCAACCAGGAAAAGTGGAG
CATCGGGTTTACTGGAGAAAACTAGCCCAGTGCCCTTCTTTTACACCCTAGA
ACCAGAGAGGAACTTGGCCATAAGCTTTTGTGCAGACTTCTCCTTGGGGGA
AAAAAAAAGTCATTATTTAAAAAGACATGACAGACTTAGACACATGCCTTAAA
TTTTAACATGCATATGTGATTCAACTTATCATTTACTGGCTTCACATTATATTT
TGCCTCTATACAAGTTTGGCTGTTTGTTTCTTATCTCTGTAGAAACTAGGAGC
AGAGCAATTATATTTATTCTTTACCTAAGGCTTTTAGAATAGATATTCTAAGAA
ATTCTGTATTTTTCTTTACACAAAACTTGACAATAGAGCTAATATGTAAGGAG
AGTCCTTTCGTTTCCTACTAATTACATTCAAGAACAACTCTGCAAGAATGTAG
AATCCTAAAATGTATACTGTGCATTAATTTCCTGTTGTGTTTAAACATAACTAT
GTCTCATATTTCGGTCTTGTATTTTTTTTACTATAATCCTTCTAGAGACAAGTG
ATCAATGAGAATCTGTTCACCAAACCAAATGTGGAAAGAACACAAAGAAGAC
ATAAGACTTCAGTCAAGTGAAAAATTAACATGTGGACTGGACACTCCAATAA
ATTATATACCTGCCTAAGTTGTACAATTTCAGAATGCAATTTTCATTATAATGA
GTTCCAGTGACTCAATGATGGGGAAAAAAATCTCTGCTCATTAATATTTCAA
GATAAAGAACAAATGTTTCCTTGAATGCTTGCTTTTGTGTGTTAGCATAATTT
TTAGAATTGTTTGAGAATTCTGATCCAAAACTTTAGTTGAATTCATCTACGTTT
GTTTAATATTAACTTAACCTATTCTATTGTATTATAATGATGATTCTGTCAAAT
GAAAGGCTTGAAATACCTAGATGAAGTTTAGATTTTCTTCCTATTGTAAACTT
TTGAGTCTGGTTTCATTGTTTTAAATAAATTAAGGGGACACTAAAGTCCTATC
ATTCATTTCCTTCATTGCTGAACAGGCAAGATATAATATTACATGAATGATTA
CTATATTTTGTTCACACTAATAAAGCTTATGCTCAGAAATGCCATACACACAC
ACACACACACACACAAACACACACATTTATCATTTAATGCATAAATCAACACA
AAAGGTTTTCCCATTAATATGAAATATTACATATATATAAGTGCCATATTTAAA
ATAATTTGTCTAACAGTAGAACTGTGTCGGAGCACTCACTGAAGCTTGCATT
CCACTGAAAGAGTTATTTGTGTAAGTAGAGTATCCGGAGAAGGAAAAGAACT
TACGACCTTTCTTTATAACAGAAACTCAACTCTAAATTCAACAAGATGTGCAA
ACCGGACATGCAGGTGAATATTTTAATAGGTTACTATAAGGTTCTCAATTAAA
TTCTTTAATCTGTCCAGTCCCAGTTTCTCTTATTAATAAAACTTTGGAAATTGC
TTTAAACCATTTAAAGGAAATTTCTAGATATAGAAACTAAGGACTGTGACTAT
ACAGCTGTCACTCATTTGTAGTAAAACTTAAAAAGCAAAAACAAAAAACAAAA
AAGACCTTCCTGTGATACTTTATTTCCGAACTAATAAAAATCTATATGACTTTT
TATTATTGTGTGATAACCAAGTAAATGTTTTCTATTTTGCATATTTTCAGGCAT
GGTAACAGAAATTTACCTTTTAATAAATTAAAAAATCTAAATTTTAACCTACTT
GTATGTTCGGAGAGTGTTTTTGTACTATATTGACTACTTAAAATAGAGAATGA
GACTAAGAAGGGAACATTTCTGTTGATACATGTTTTTTAAAAGTAATTTTAAG
AGCATTATTAGGTTAATTAATCCAATTAATGACCCAAATGCCAAGGTAATTTT
AAATTTACATTTTTAATAAAAGCAACATGTTGAAACAAGAGAGGGTGAGATTA
ACCTTTTTGCTAAAGTAATTTACAAGTCAAAGACAGGAAGAGATCAGAGTGA
ATGTGCCTTCTTAACCAGAGCTACAGAATTTAGTGAATAATTAAAGTACAAAC
TGCTTTGACCTCCTTGAACTTTTCCAAGCAATTTCTCTGTACTTCTATATATG
AATGTCTTAGCCAATTTTCTGCTACTATAACAGAATACGACAGACTGGGTAA

TTTAAAAAGAAAAGAAATTTATTTTCTTCCTAGTTCTGGAGGCTGGGAAGGC
GAAGGGCATGGCACTGACATCTGCCTTGTAACTGATGAGAACCTTCTTACT
GCATGATAACAAAGCAGCAAGGCAAGCAAAAGCGTAAGATGAAGAGAGAGG
AAATGAAGCCAAACACATCCTTTCATCAGAAGCCCATTCCCTCTATAAGGCG
TTATTACATTTATGAGAATGGAGTCCTCATGACCTAATCGTGACCTTAAAGG
CCCCTCCCAACACTGTTACAATGGCAATTAAATTTCAACAAAGGTTCCAGAG
GTGACATTCGAATCAGCAATGAAATTTTCATAGTTAAATTTGGTATTCGTGGG
GGAAGAAATGACCATTTCCCTTGTATTTTTATAATTAAATCAGCAAAATATTG
TAATAAAGAAATCTTTCCTGTGAAGATACCATGACCCCAn

Selected Module Sets:
Left Module Count: 3
Spacer Nucleotide Count: 5,6,7
Right Module Count: 3
Ignore Asp Overlap: False

The results below are zinc finger arrays that can be constructed using CoDA. Note that other methods (including modular assembly and OPEN) can also potentially be used to target the input sequence of interest."

Sort By: Position **Hide intron splice sites**

ZFN-GI|224589816:C145061904-145030456_HOMO_SAPIENS_CHROMOSOME_4,_GRCH37.P2_PRIMARY_REFERENCE_ASSEMBLY-SP-7-1
266 tTTCCTCTTCCAGTCTGGTGGATGGAt 292
266 aAAGGAGAAGGTCAGACCACCTACCTa 292

FINGER	HELIX	TRIPLET	REFERENCE NUMBER	SOURCE
Left F1	QASNLLR	GAA	-	CoDA
Left F2	RQDNLGR	GAG	-	CoDA
Left F3	QRNNLGR	GAA	-	CoDA
Right F1	DKTKLRV	GGA	-	CoDA
Right F2	VRHNLTR	GAT	-	CoDA
Right F3	RRAALGP	GTG	-	CoDA

ZF DNA Sequence Bos taurus (cow) Build 3.1

Blast TTCCTCTTCNNNNNNNGTGGATGGA

ZFN-GI|224589816:C145061904-145030456_HOMO_SAPIENS_CHROMOSOME_4,_GRCH37.P2_PRIMARY_REFERENCE_ASSEMBLY-SP-7-2
7277 cTGCCTCAGCCTCCCAAGTAGCTGAGa 7303
7277 gACGGAGTCGGAGGGTTCATCGACTCt 7303

FINGER	HELIX	TRIPLET	REFERENCE NUMBER	SOURCE

Left F1	RRVDLLR	GCA	-	CoDA
Left F2	RQDNLGR	GAG	-	CoDA
Left F3	VSNTLTR	GCT	-	CoDA
Right F1	KHSNLTR	GAG	-	CoDA
Right F2	QRSDLTR	GCT	-	CoDA
Right F3	QSGTLTR	GTA	-	CoDA

ZF DNA Sequence Bos taurus (cow) Build 3.1

Blast TGCCTCAGCNNNNNNNGTAGCTGAG

ZFN-GI|224589816:C145061904-145030456_HOMO_SAPIENS_CHROMOSOME_4,_GRCH37.P2_PRIMARY_REFERENCE_ASSEMBLY-SP-6-1

7278 tGCCTCAGCCTCCCAAGTAGCTGAGa 7303

7278 aCGGAGTCGGAGGGTTCATCGACTCt 7303

FINGER	HELIX	TRIPLET	REFERENCE NUMBER	SOURCE
Left F1	APSKLKR	GGC	-	CoDA
Left F2	QREHLTT	TGA	-	CoDA
Left F3	ESGHLKR	GGC	-	CoDA
Right F1	KHSNLTR	GAG	-	CoDA
Right F2	QRSDLTR	GCT	-	CoDA
Right F3	QSGTLTR	GTA	-	CoDA

ZF DNA Sequence Bos taurus (cow) Build 3.1

Blast GCCTCAGCCNNNNNNNGTAGCTGAG

ZFN-GI|224589816:C145061904-145030456_HOMO_SAPIENS_CHROMOSOME_4,_GRCH37.P2_PRIMARY_REFERENCE_ASSEMBLY-SP-7-3

8186 cAGCTTCTGCCTCTCAAGTAGCTGAGa 8212

8186 gTCGAAGACGGAGAGTTCATCGACTCt 8212

FINGER	HELIX	TRIPLET	REFERENCE NUMBER	SOURCE
Left F1	QRQALDR	GCT	-	CoDA
Left F2	QQTNLTR	GAA	-	CoDA
Left F3	QGNTLTR	GCA	-	CoDA
Right F1	KHSNLTR	GAG	-	CoDA
Right F2	QRSDLTR	GCT	-	CoDA
Right F3	QSGTLTR	GTA	-	CoDA

ZF DNA Sequence Bos taurus (cow) Build 3.1

Blast AGCTTCTGCNNNNNNNGTAGCTGAG

ZFN-GI|224589816:C145061904-145030456_HOMO_SAPIENS_CHROMOSOME_4,_GRCH37.P2_PRIMARY_REFERENCE_ASSEMBLY-SP-6-2
8812 tCCTAGCTACTTGGGAGGCTGAGGCa 8837
8812 aGGATCGATGAACCCTCCGACTCCGt 8837

FINGER	HELIX	TRIPLET	REFERENCE NUMBER	SOURCE
Left F1	RSSHLKM	AGG	-	CoDA
Left F2	QRSDLTR	GCT	-	CoDA
Left F3	QSGTLTR	GTA	-	CoDA
Right F1	APSKLKR	GGC	-	CoDA
Right F2	QREHLTT	TGA	-	CoDA
Right F3	ESGHLKR	GGC	-	CoDA

ZF DNA Sequence Bos taurus (cow) Build 3.1

Blast CCTAGCTACNNNNNNGGCTGAGGC

ZFN-GI|224589816:C145061904-145030456_HOMO_SAPIENS_CHROMOSOME_4,_GRCH37.P2_PRIMARY_REFERENCE_ASSEMBLY-SP-7-4
8812 tCCTAGCTACTTGGGAGGCTGAGGCAg 8838
8812 aGGATCGATGAACCCTCCGACTCCGTc 8838

FINGER	HELIX	TRIPLET	REFERENCE NUMBER	SOURCE
Left F1	RSSHLKM	AGG	-	CoDA
Left F2	QRSDLTR	GCT	-	CoDA
Left F3	QSGTLTR	GTA	-	CoDA
Right F1	RRVDLLR	GCA	-	CoDA
Right F2	RQDNLGR	GAG	-	CoDA
Right F3	VSNTLTR	GCT	-	CoDA

ZF DNA Sequence Bos taurus (cow) Build 3.1

Blast CCTAGCTACNNNNNNNGCTGAGGCA

ZFN-GI|224589816:C145061904-145030456_HOMO_SAPIENS_CHROMOSOME_4,_GRCH37.P2_PRIMARY_REFERENCE_ASSEMBLY-SP-6-3
11886 tTTCAGCTGCAAGTCAGGAGCGAACc 11911
11886 aAAGTCGACGTTCAGTCCTCGCTTGg 11911

FINGER	HELIX	TRIPLET	REFERENCE NUMBER	SOURCE
Left F1	TTTNLRR	GAA	-	CoDA
Left F2	QRSDLTR	GCT	-	CoDA
Left F3	QGGTLRR	GCA	-	CoDA

Right F1	GHTALAL	AAC	-	CoDA
Right F2	RTDTLAR	GCG	-	CoDA
Right F3	QTTHLSR	GGA	-	CoDA

ZF DNA Sequence Bos taurus (cow) Build 3.1

Blast TTCAGCTGCNNNNNNGGAGCGAAC

ZFN-GI|224589816:C145061904-145030456_HOMO_SAPIENS_CHROMOSOME_4,_GRCH37.P2_PRIMARY_REFERENCE_ASSEMBLY-SP-7-5
12101 aGCATGCAGCAGAGAGCTAGGAGGCTg 12127
12101 tCGTACGTCGTCTCTCGATCCTCCGAc 12127

FINGER	HELIX	TRIPLET	REFERENCE NUMBER	SOURCE
Left F1	RNRNLVL	TGC	-	CoDA
Left F2	QSTTLKR	GCA	-	CoDA
Left F3	LKHDLRR	GCT	-	CoDA
Right F1	NKQALDR	GCT	-	CoDA
Right F2	RQDNLGR	GAG	-	CoDA
Right F3	RPESLRP	TAG	-	CoDA

ZF DNA Sequence Bos taurus (cow) Build 3.1

Blast GCATGCAGCNNNNNNNTAGGAGGCT

ZFN-GI|224589816:C145061904-145030456_HOMO_SAPIENS_CHROMOSOME_4,_GRCH37.P2_PRIMARY_REFERENCE_ASSEMBLY-SP-7-6
12104 aTGCAGCAGAGAGCTAGGAGGCTGGCt 12130
12104 tACGTCGTCTCTCGATCCTCCGACCGa 12130

FINGER	HELIX	TRIPLET	REFERENCE NUMBER	SOURCE
Left F1	HNGTLKR	GCA	-	CoDA
Left F2	QRSDLTR	GCT	-	CoDA
Left F3	QRNTLKG	TCT	-	CoDA
Right F1	APSKLAR	GGC	-	CoDA
Right F2	QRSDLTR	GCT	-	CoDA
Right F3	RQDNLQR	GAG	-	CoDA

ZF DNA Sequence Bos taurus (cow) Build 3.1

Blast TGCAGCAGANNNNNNNGAGGCTGGC

ZFN-GI|224589816:C145061904-145030456_HOMO_SAPIENS_CHROMOSOME_4,_GRCH37.P2_PRIMARY_REFERENCE_ASSEMBLY-SP-7-7

14164 tGCCTTCTTCTTACCACTAAGCATGCa 14190
14164 aCGGAAGAAGAATGGTGATTCGTACGt 14190

FINGER	HELIX	TRIPLET	REFERENCE NUMBER	SOURCE
Left F1	TRAKLHI	GGC	-	CoDA
Left F2	QQTNLTR	GAA	-	CoDA
Left F3	QTNNLNR	GAA	-	CoDA
Right F1	RNRNLVL	TGC	-	CoDA
Right F2	QSTTLKR	GCA	-	CoDA
Right F3	QGGNLAL	TAA	-	CoDA

ZF DNA Sequence Bos taurus (cow) Build 3.1

Blast GCCTTCTTCNNNNNNNTAAGCATGC

ZFN-GI|224589816:C145061904-145030456_HOMO_SAPIENS_CHROMOSOME_4,_GRCH37.P2_PRIMARY_REFERENCE_ASSEMBLY-SP-6-4
15408 cCTCTTCAGCCTCTCAGTTGCTGTTc 15433
15408 gGAGAAGTCGGAGAGTCAACGACAAg 15433

FINGER	HELIX	TRIPLET	REFERENCE NUMBER	SOURCE
Left F1	KHSNLTR	GAG	-	CoDA
Left F2	QQTNLTR	GAA	-	CoDA
Left F3	VGNSLTR	GCT	-	CoDA
Right F1	QATLLRR	GTT	-	CoDA
Right F2	QRSDLTR	GCT	-	CoDA
Right F3	IRTSLKR	GTT	-	CoDA

ZF DNA Sequence Bos taurus (cow) Build 3.1

Blast CTCTTCAGCNNNNNNGTTGCTGTT

ZFN-GI|224589816:C145061904-145030456_HOMO_SAPIENS_CHROMOSOME_4,_GRCH37.P2_PRIMARY_REFERENCE_ASSEMBLY-SP-6-5
17540 aGACAGCATCAAGGGGGATGATGTTa 17565
17540 tCTGTCGTAGTTCCCCCTACTACAAt 17565

FINGER	HELIX	TRIPLET	REFERENCE NUMBER	SOURCE
Left F1	TRAVLRR	GTC	-	CoDA
Left F2	QRSDLTR	GCT	-	CoDA
Left F3	LTHNLRR	GAT	-	CoDA
Right F1	TNQALGV	GTT	-	CoDA
Right F2	VRHNLTR	GAT	-	CoDA
Right F3	ISHNLAR	GAT	-	CoDA

ZF DNA Sequence Bos taurus (cow) Build 3.1

Blast GACAGCATCNNNNNNGATGATGTT

ZFN-GI|224589816:C145061904-145030456_HOMO_SAPIENS_CHROMOSOME_4,_GRCH37.P2_PRIMARY_REFERENCE_ASSEMBLY-SP-7-8

20865 gAGCCGCTGCTGGGAGGGATGTGGGAg 20891

20865 cTCGGCGACGACCCTCCCTACACCCTc 20891

FINGER	HELIX	TRIPLET	REFERENCE NUMBER	SOURCE
Left F1	KHQTLQR	GCT	-	CoDA
Left F2	RTDTLAR	GCG	-	CoDA
Left F3	QKGTLGR	GCA	-	CoDA
Right F1	THAHLTR	GGA	-	CoDA
Right F2	RREVLEN	GTG	-	CoDA
Right F3	ISHNLAR	GAT	-	CoDA

ZF DNA Sequence Bos taurus (cow) Build 3.1

Blast AGCCGCTGCNNNNNNNGATGTGGGA

ZFN-GI|224589816:C145061904-145030456_HOMO_SAPIENS_CHROMOSOME_4,_GRCH37.P2_PRIMARY_REFERENCE_ASSEMBLY-SP-7-9

24774 gTGCTTCAGCCTCCGCAGTAGTTGGGa 24800

24774 cACGAAGTCGGAGGCGTCATCAACCCt 24800

FINGER	HELIX	TRIPLET	REFERENCE NUMBER	SOURCE
Left F1	RGQELRR	GCA	-	CoDA
Left F2	QQTNLTR	GAA	-	CoDA
Left F3	VGNSLTR	GCT	-	CoDA
Right F1	KSNHLHV	GGG	-	CoDA
Right F2	HKSSLTR	GTT	-	CoDA
Right F3	QSTSLQR	GTA	-	CoDA

ZF DNA Sequence Bos taurus (cow) Build 3.1

Blast TGCTTCAGCNNNNNNNGTAGTTGGG

ZFN-GI|224589816:C145061904-145030456_HOMO_SAPIENS_CHROMOSOME_4,_GRCH37.P2_PRIMARY_REFERENCE_ASSEMBLY-SP-7-10

26726 tCTCCACTCCCAGGTGCTAAGCAGATa 26752

26726 aGAGGTGAGGGTCCACGATTCGTCTAt 26752

FINGER	HELIX	TRIPLET	REFERENCE NUMBER	SOURCE

Left F1	KHSNLTR	GAG	-	CoDA
Left F2	RREVLEN	GTG	-	CoDA
Left F3	QKPHLSR	GGA	-	CoDA
Right F1	SKQALAV	GAT	-	CoDA
Right F2	QSTTLKR	GCA	-	CoDA
Right F3	QGGNLAL	TAA	-	CoDA

ZF DNA Sequence   Bos taurus (cow) Build 3.1

Blast CTCCACTCCNNNNNNNTAAGCAGAT

ZFN-GI|224589816:C145061904-145030456_HOMO_SAPIENS_CHROMOSOME_4,_GRCH37.P2_PRIMARY_REFERENCE_ASSEMBLY-SP-5-1
27940 aGTCTTCGTCCTTTAGAAGCTGAGa 27964
27940 tCAGAAGCAGGAAATCTTCGACTCt 27964

FINGER	HELIX	TRIPLET	REFERENCE NUMBER	SOURCE
Left F1	DEANLRR	GAC	-	CoDA
Left F2	QQTNLTR	GAA	-	CoDA
Left F3	DMGNLGR	GAC	-	CoDA
Right F1	KHSNLTR	GAG	-	CoDA
Right F2	QRSDLTR	GCT	-	CoDA
Right F3	QRNNLGR	GAA	-	CoDA

ZF DNA Sequence   Bos taurus (cow) Build 3.1

Blast GTCTTCGTCNNNNNGAAGCTGAG

ZFN-GI|224589816:C145061904-145030456_HOMO_SAPIENS_CHROMOSOME_4,_GRCH37.P2_PRIMARY_REFERENCE_ASSEMBLY-SP-7-11
28378 gCACCTCACAGAAAACATAGGAAAACa 28404
28378 cGTGGAGTGTCTTTTGTATCCTTTTGt 28404

FINGER	HELIX	TRIPLET	REFERENCE NUMBER	SOURCE
Left F1	RRHILDR	GTG	-	CoDA
Left F2	RQDNLGR	GAG	-	CoDA
Left F3	QQHGLRH	TGT	-	CoDA
Right F1	GASALRQ	AAC	-	CoDA
Right F2	QQTNLTR	GAA	-	CoDA
Right F3	RRDHLSL	TAG	-	CoDA

ZF DNA Sequence   Bos taurus (cow) Build 3.1

Blast CACCTCACANNNNNNNTAGGAAAAC

ZFN-GI|224589816:C145061904-145030456_HOMO_SAPIENS_CHROMOSOME_4,_GRCH37.P2_PRIMARY_REFERENCE_ASSEMBLY-SP-6-6

31017 tTTCTTCCTAGTTCTGGAGGCTGGGa 31042

31017 aAAGAAGGATCAAGACCTCCGACCCt 31042
